# The *Sapria himalayana* genome provides new insights into the lifestyle of endoparasitic plants

**DOI:** 10.1186/s12915-023-01620-3

**Published:** 2023-06-06

**Authors:** Xuelian Guo, Xiaodi Hu, Jianwu Li, Bingyi Shao, Yajun Wang, Long Wang, Kui Li, Dongliang Lin, Hanchen Wang, Zhiyuan Gao, Yuannian Jiao, Yingying Wen, Hongyu Ji, Chongbo Ma, Song Ge, Wenkai Jiang, Xiaohua Jin

**Affiliations:** 1 State Key Laboratory of Systematic and Evolutionary Botany, Institute of Botany, Chinese Academy of Sciences (IBCAS), Beijing, 100093 China; 2 Novogene Bioinformatics Institute, Beijing, 100083 China; 3 Xishuangbanna Tropical Botanical Garden, Chinese Academy of Sciences, Menglun Township, Mengla County, Yunnan, 666303 China

**Keywords:** *Sapria himalayana*, Endophyte, Genome, Horizontal gene transfer, Flower development, fatty acid biosynthesis

## Abstract

**Background:**

*Sapria himalayana* (Rafflesiaceae) is an endoparasitic plant characterized by a greatly reduced vegetative body and giant flowers; however, the mechanisms underlying its special lifestyle and greatly altered plant form remain unknown. To illustrate the evolution and adaptation of *S. himalayasna*, we report its de novo assembled genome and key insights into the molecular basis of its floral development, flowering time, fatty acid biosynthesis, and defense responses.

**Results:**

The genome of *S. himalayana* is ~ 1.92 Gb with 13,670 protein-coding genes, indicating remarkable gene loss (~ 54%), especially genes involved in photosynthesis, plant body, nutrients, and defense response. Genes specifying floral organ identity and controlling organ size were identified in *S. himalayana* and *Rafflesia cantleyi*, and showed analogous spatiotemporal expression patterns in both plant species. Although the plastid genome had been lost, plastids likely biosynthesize essential fatty acids and amino acids (aromatic amino acids and lysine). A set of credible and functional horizontal gene transfer (HGT) events (involving genes and mRNAs) were identified in the nuclear and mitochondrial genomes of *S. himalayana*, most of which were under purifying selection. Convergent HGTs in *Cuscuta*, Orobanchaceae, and *S. himalayana* were mainly expressed at the parasite–host interface. Together, these results suggest that HGTs act as a bridge between the parasite and host, assisting the parasite in acquiring nutrients from the host.

**Conclusions:**

Our results provide new insights into the flower development process and endoparasitic lifestyle of Rafflesiaceae plants. The amount of gene loss in *S. himalayana* is consistent with the degree of reduction in its body plan. HGT events are common among endoparasites and play an important role in their lifestyle adaptation.

**Supplementary Information:**

The online version contains supplementary material available at 10.1186/s12915-023-01620-3.

## Background

Parasitism in angiosperms has independently evolved at least 12 times from their free-living ancestors [1]. Parasitic plants acquire some or all of their water, nutrients, nucleic acids (DNA, RNA), and proteins through a specialized organ called haustorium, which establishes a direct physical connection with the roots or stems of their hosts [2]. Endoparasitic plants spend most stages of their life, except flower emergence, within host tissues [3]. One of the endoparasitic species, *Rafflesia arnoldii,* even boasts the largest flower in the world, measuring up to 1 m in diameter and weighing up to 7 kg [4].

**Fig. 1:**
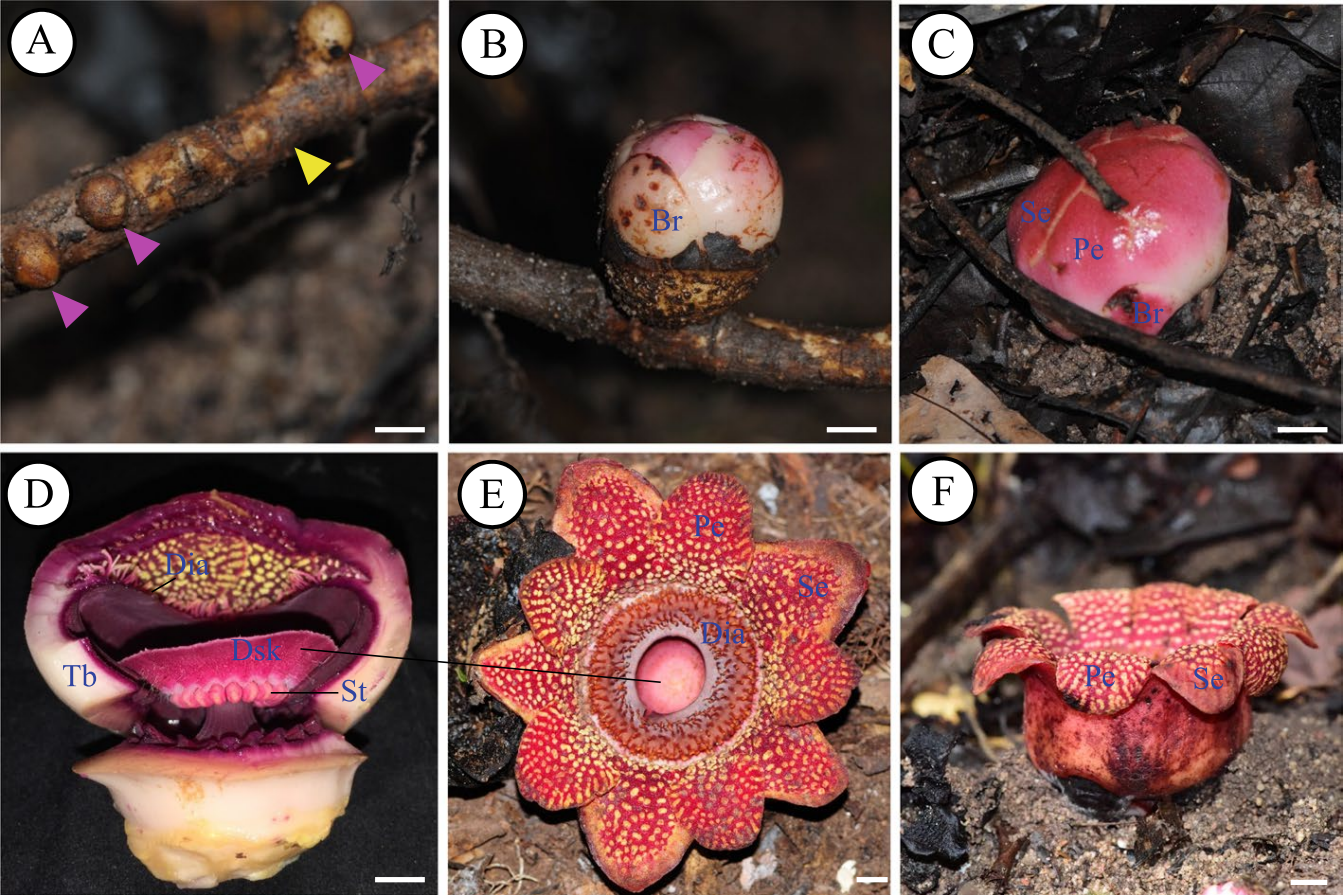
*Sapria himalayana* male flower. **A ***S. himalayana* (rose arrow) residing within the host roots (yellow arrow). **B**, **C** Flower bud. **D** Longitudinal section of the flower bud. **E**, **F** Top (**E**) and lateral (**F**) view of a flower. Scale bars, 1 cm. Br, bract; Dia, diaphragm; Dsk, disk; Pe, petal; Se, sepal; St, stamen; Tb, perianth tube. Tissues from Br, Pe, Se, and Dsk + St were used as materials for transncriptome, respectively

*Sapria himalayana* (Rafflesiaceae) is a dioecious endoparasitic plant characterized by its greatly reduced vegetative body and very large flowers [5] (Fig. 1). The vegetative body of *S. himalayana* is a mycelium-like structure that exists in the roots of the vine host, *Tetrastigma* spp. [3]. Plastid genome has been completely lost in Rafflesiaceae plants [6, 7]. This extremely specialized lifestyle of endoparasitic plants raises many important questions regarding, for example, the genomic characteristics of endoparasitic plants, the mechanism that underpins the formation of gigantic flowers, and the functioning of circadian clock in *S. himalayana*, especially with respect to the mechanisms affecting flowering time. For instance, dodder (*Cuscuta campestris*) synchronizes its flowering time with that of its host using the host-synthesized floral signal, FLOWERING LOCUS T (FT) [8]; however, the flowering time of *S. himalayana* (September to December) does not coincide with that of its host (April to June). The specialized lifestyle of *S. himalayana* also questions the mechanism of horizontal gene transfer (HGT) between itself and its intimately connected host, and the biosynthesis mechanism of fatty acids (FAs), aromatic amino acids (AAAs; phenylalanine, tyrosine, and tryptophan), and lysine, which are essential for plant growth and development and are usually de novo synthesized in the plastid [9–11].

Cai et al. [6] assembled the draft genome of *S. himalayana* (NCBI accession: PRJNA686196), with 55,179 predicted genes and 42,512 authentic genes, using long read-derived scaffolded contigs and Illumina short reads (10X Genomics) (Table 1). The draft genome was 1.28 Gb in size, accounting for ~ 40% of the estimated genome size [6] (Table 1), and provided new insights into genome evolution in endophytic plants and HGTs in *S. himalayana* [6]. However, because of the complex nature of the *S. himalayana* genome and the limited availability of transcriptomic, genomic, and annotation data, these questions concerning endoparasitic plants need to be investigated further.

**Table 1:** Comparison of genome features of two versions of *Sapria himalayana*

Feature	*S. himalayana* (in the study)	*S. himalayana* (Cai et al., 2021)
Technology	PacBio/10X/Illumina	Nanopore/Illumina
Final assemble genome size	1.92 Gb	1.28 Gb
Assembly completion (%)	70%	40%
Contig number and N50	26,955 (N50: 104.22 Kb)	216,625 (19.95 Kb)
Scaffold number and N50	18,718 (N50: 245.75 Kb)	128,027 (N50: 952.63 Kb)
NG50	112.61 Kb	29.62 Kb
Repeat motif (%)	86.41%	89.6%
GC content (%)	27.47%	24%
Gene number	13,670	55,179 (42,512 authentic)
BUSCO (1,440, Embryophytes)	47.9%	44.6%
Nuclear HGT number (phylogenomic analysis)	98	90

To improve the *S. himalayana* genome quality and to explore mechanisms underpinning its endoparasitic lifestyle, giant flower phenotype and HGTs, we de novo assembled the genome of *S. himalayana* using PacBio long reads, 10X Genomics data, and transcriptome data, eventually obtaining a well-assembled and annotated genome of 1.92 Gb. We unveil remarkable features of the nuclear genome of this endophytic species, and report key insights into the molecular basis of floral development, flowering time, and defense responses. We also illustrate features of the mitochondrial genome of *S. himalayana* for understanding mitogenome evolution in endophytes. Our observations will be of value to future developmental and ecological studies aimed at understanding the mechanisms and evolutionary basis of plant–plant interactions.

## Results

### Genome sequencing, assembly, and annotation

The genome size of *S. himalayana* was estimated as 2,822.01 Mb by *k*-mer analysis [12], with repeat and heterozygosity percentages of 86.41% and 0.58%, respectively (Table 1; Additional file 1: Fig. S1). Whole-genome sequencing using the PacBio Sequel platform and 10X Genomics yielded 353.49 Gb (125.26X coverage) and 301.75 Gb (106.93X coverage) data, respectively. PacBio long reads were assembled using FALCON [13], polished with Quiver, and further scaffolded by integrating with 10X Genomics. Moreover, 191.29 Gb (67.79X) of Illumina short reads were examined by Pilon [14] to determine the sequence error in final contigs. The assembly consisted of 18,718 scaffolds with sizes up to 1.92 Gb, featuring a scaffold N50 and contig N50 value of 245.75 and 104.22 kb, respectively (Table 1), and a maximum scaffold length of 14.30 Mb (Additional file 2: Table S1).

Benchmarking Universal Single Copy Orthologs (BUSCO) analysis [15] was performed to assess the genome completeness, and the results showed that 44.1%, 47.9%, and 95.7% of the genes were complete and conserved in eudicots, embryophytes, and eukaryotes, respectively, and were present in the assembly (Additional file 2: Table S2). Additionally, 98.76% of the Illumina short reads could be mapped back to the assembled contigs using the Burrows-Wheeler Aligner (BWA) software [16] (Additional file 2: Table S1). The transcriptome reads generated from various tissues (such as sepal, petal, disk with anthers, and four transverse layers of a flower bud) showed high mapping rates (Fig. 1; Additional file 1: Fig. S2; Additional file 2: Table S3). Specifically, total RNA isolated from the four above-mentioned transverse layer tissues and pooled in equimolar amounts was also subjected to isoform sequencing (Iso-seq), producing non-redundant 10,367 transcripts (Additional file 2: Table S3). Taken together, these results indicate that the assembly contains comprehensive genomic information.

Homology-based prediction, de novo gene structure prediction, and transcriptome-assisted prediction revealed a total of 13,670 protein-coding genes in the *S. himalayana* genome, with an average gene length of 18,296 bp and an average coding sequence (CDS) length of 1,029 bp (Table 1; Additional file 2: Table S4). In total, 13,408 genes, accounting for 98.10% of the predicted protein-coding genes, were annotated in at least one functional database (Additional file 2: Table S5). Among the four major types of repeats identified (DNA transposon, LINE, LTR, and unknown repeats), DNA transposons comprised the largest proportion of the genome (33.53%; total length, 691.1 Mb), followed by LTRs (23.08%; total length, 475.6 Mb), LINEs (12.81%), and unknown repeats (15.46%) (Additional file 2: Table S6). Additionally, a total of 4,815 non-coding RNAs (ncRNAs) were predicted in the genome, including 3,409 ribosomal RNAs (rRNAs; 904 28S, 2,279 18S, 198 5.8S, and 28 5S rRNAs), 724 microRNAs (miRNAs), 539 transfer RNAs (tRNAs), and 143 small nuclear RNAs (snRNAs) (Additional file 2: Table S7).

### Massive gene loss in the nuclear genome

**Fig. 2:**
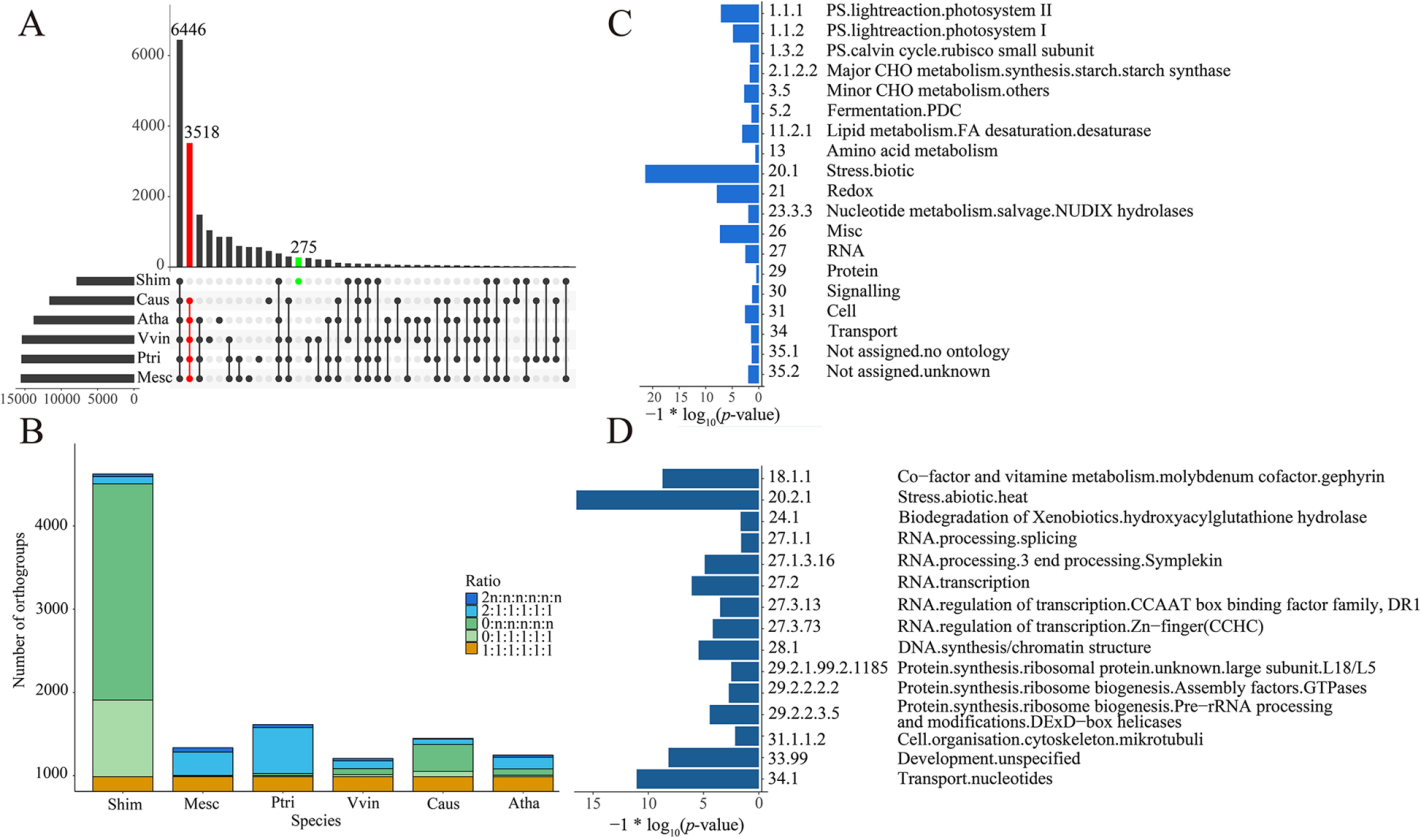
Gene loss and duplication in *Sapria himalayana* genome. **A** UpSet Venn diagram showing the overview of orthogroups from *Arabidopsis thaliana* (Atha), *Cuscuta australis* (Caus), *Manihot esculenta* (Mesc), *Populus trichocarpa* (Ptri), *S. himalayana* (Shim), and *Vitis vinifera* (Vvin). The bar diagram (left) shows the number of orthogroups found in each species; the bar diagram (upper panel) and lattice diagram (lower panel) together show the number of overlapping and exclusive orthogroups found in the six species. The lost and species-specific orthogroups ib *S. himalayana* are represented by red and green dots, respectively. **B** Bar diagram showing the gene content in orthogroups in *S. himalayana* and other representative species. Orthogroups with exactly one gene in each species are indicated in orange. Orthogroups missing in *S. himalayana* but are present in each species (exactly 0:1, named: other species, pale green; 0:n (*n* > 1), green). Duplicated orthogroups were defined as having exactly 2:1 genes per species (wathet) or as having a ratio of at least 2n:n(n > 1) genes in each species (blue). **C**, **D** Functional enrichment analysis of genes either lost (ratio 0:1 or more, **C**) or duplicated (at least 2n:n, **D**) in *S. himalayana*. MapMan categories of significantly enriched genes are displayed using the logarithmic values of their *P*-values

The *S. himalayana* genome contains the lowest number of gene orthologs compared with the genomes of five other plant species including *Arabidopsis thaliana* (phytozome v12), *Cuscuta australis* (http://groups.english.kib.cas.cn/epb/dgd/), *Manihot esculenta*, *Populus trichocarpa*, and *Vitis vinifera* (phytozome v12), as expected. Approximately 3,518 orthogroups (54.58%), which are conserved among the other five species, were lost in *S. himalayana* (Fig. 2A; Additional file 2: Table S8), consistent with the results of Cai et al. [6]. However, 275 orthogroups were unique to *S. himalayana* (Fig. 2A). The number of genes lost in *S. himalayana* was considerably higher than that duplicated (Fig. 2B). The MapMan categories enriched in the lost gene set included “stress. biotic” and “photosynthesis. lightreaction” (Fig. 2C; Additional file 2: Table S9). By contrast, categories including “stress. abiotic. heat”, “transport nucleotides”, and “co-factor and vitamin metabolism. molybdenum cofactor. gephyrin” were notably enriched in the duplicated gene set (Fig. 2D; Additional file 2: Table S10). The “RNA. regulation of transcription” and “protein. degradation” categories were enriched in the species-specific dataset (Additional file 1: Fig. S3; Additional file 2: Table S11). However, functions of the majority of species-specific genes in *S. himalayana* were unknown (Additional file 2: Table S11).

Considering the largely altered body plan of *S. himalayana*, we specifically inspected the genes responsible for its root and leaf development, and found that genes related to leaf initiation, root development, root gravity perception, polar auxin transport, and nitrogen, phosphate, and potassium uptake [17–22] were conspicuously missing in the *S. himalayana* genome (Additional file 2: Table S12, S13).

### Genes involved in flower development

**Fig. 3:**
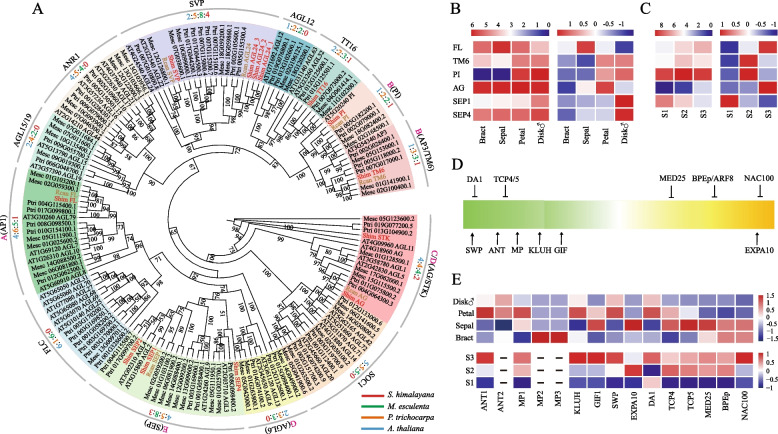
Underlying molecular developmental genetics of a highly giant flower in Sapria himalayana. **A** Phylogenetic tree of the MIKC-type MADS-box gene family. Genes from S. himalayana and Rafflesia cantleyi are highlighted in red and brown colors, respectively. The numbers of genes were also shown and coloured according to the species colour scheme. Species names are abbreviated as follows: Arabidopsis thaliana (Atha), Manihot esculenta (Mesc), Populus trichocarpa (Ptri), R. cantleyi (Rcan), and S. himalayana (Shim). BS > 50% are shown. **B**, **C** Heatmap showing the log2-tranformed (left) and z-score transformed (right) gene expression values in flower tissues in S. himalayana and R. cantleyi, respectively. Disk♂, Disk with stamens. S1, floral bud is made up of undifferentiated masses of cells; S2, floral bud contains moderately differentiated and visible internal organs; S3, floral bud consists of more developed internal organs. **D**, **E** Genes involved in the regulation of petal growth. Genes promoting/inhibiting cell proliferation and expansion in petal were summarized in A. thaliana. Arrows and T-ends represent promoting and inhibiting genetic interactions, respectively (**D**); Heatmap showing the z-score transformed gene expression data, upper section from flower tissues of S. himalayana, and lower section from three different flower development stages of R. cantleyi (**E**). ‘-’ represents absence

The flowers of *Sapria* species are distinct among angiosperms, and are characterized by 10 bright-red perianth lobes in two whorls. The ABCDE model of floral development usually provides a predictive framework for verifying the identities of floral organs. In total, 21 *MADS-box* genes were identified in *S. himalayana* genome, 13 of which belong to the MIKC-type subfamily (Fig. 3A; Additional file 1: Fig. S4). Phylogenetic analyses of these genes showed that the orthologs of ABCDE model prototype genes were all present in the *S. himalayana* genome, and only one homolog for each of five types of floral organ identity genes clustered with their corresponding ortholog in *R. cantleyi* (Fig. 3A). Although *euAP3* was not found in *S. himalayana* genome, the *paleoAP3*-type *TOMATO MADS-BOX GENE6* (*TM6*) gene, responsible for petal identity in core eudicots [23], was expressed in the floral organs of *S. himalayana* (Fig. 3A, B; Additional file 1: Fig. S5A). Noticeably, *TM6* showed the highest expression in the inner perianth whorl (Fig. 3B). We speculated that the inner perianth lobes are petals, and the function of *euAP3* is performed by *TM6*, which interacts with *PI* and specifies petal identity. In *S. himalayana*, the FRUITFUL-like (FL; A-type gene) homolog showed the highest expression level in the outer perianth whorl (Fig. 3B; Additional file 1: Fig. S5B), suggesting that the outer perianth lobes are probably sepals. All floral organ identity genes were also expressed at different developmental stages in *R. cantleyi* plants, which possess one perianth whorl (Fig. 3C).

**Fig. 4:**
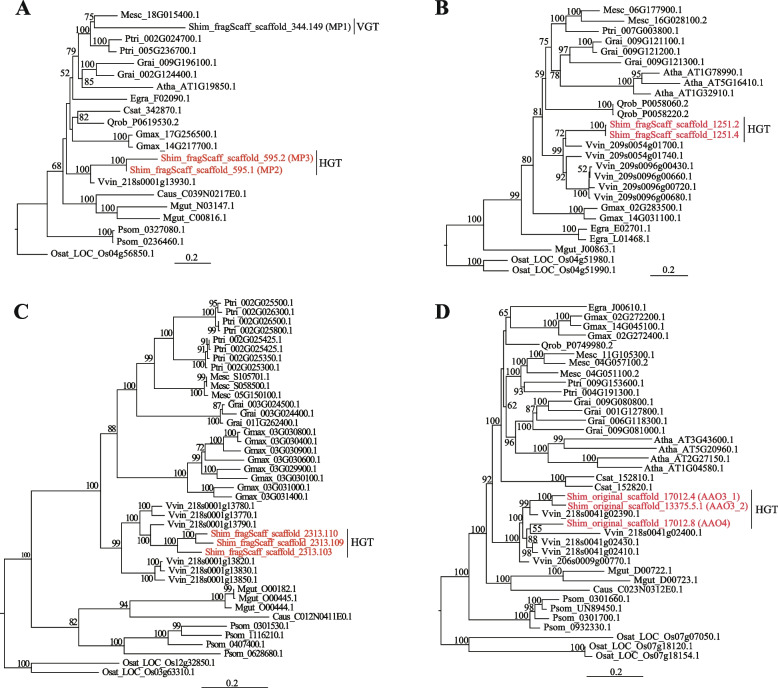
Horizontal gene transfer (HGT) events in the nuclear genome of Sapria himalayana. **A** MONOPTEROS/AUXIN RESPONSE FACTOR 5 (MP/ARF5). **B** HXXXD-type acyl-transferase family protein. **C** ALTERED TRYPTOPHAN REGULATION 4 (ATR4). **D** Abscisic-aldehyde oxidase 3/4 (AAO3/4). Species names are abbreviated as follows: Arabidopsis thaliana (Atha), Cucumis sativus (Csat), Cuscuta australis (Caus), Eucalyptus grandis (Egra), Glycine max (Gmax), Gossypium raimendii (Grai), Manihot esculenta (Mesc), Mimulus guttatus (Mgut), Oryza sativa (Osat), Papaver somniferum (Psom), Populus trichocarpa (Ptri), Quercus robur (Qrob), S. himalayana (Shim), and Vitis vinifera (Vvin). HGT events are highlighted in red. VGT, vertical gene transfer

Rafflesiaceae plants, possessing a massive flower with greatly reduced vegetative organs, are especially puzzling in the plant kingdom because their closest relatives possess minute flowers [4]. In *Arabidopsis*, petals grow via basipetal waves of cell division, followed by a phase of cell expansion [24]. Genes involved in petal cell proliferation and expansion were identified in *S. himalayana* (Fig. 3D), such as *MONOPTEROS*/*AUXIN RESPONSE FACTOR 5* (*MP*/*ARF5*), an auxin-responsive TF gene that promotes cell proliferation in the early phase of petal growth [24]. Three copies of *MP* were detected in the *S. himalayana* genome (Additional file 2: Table S15), two of which were HGTs from the host (Fig. 4A). *MP1* showed a high level of expression in petals and sepals (Fig. 3E). *AINTEGUMENTA* (*ANT*), another critical TF gene involved in proliferative flower growth and activated by *MP* or in parallel with *MP* [25, 26], was present as two copies in the *S. himalayana* genome and was expressed to high levels in petals and reproductive disk (disk with stamen) (Fig. 3E). The expression patterns of *GRF-INTERACTING FACTOR 1* (*GIF1*) and *STRUWWELPETER* (*SWP*), which promote cell proliferation and the formation of big petals [27–29], were similar to that of *MP1* in *S. himalayana* (Fig. 3E). *P450 KLUH*/*CYP78A5*, a stimulator of plant organ growth [30, 31], also showed high-level expression in petals and reproductive disk (Fig. 3E). *MEDIATOR COMPLEX SUBUNIT 25* (*MED25*) and *BIGPETALp* (*BPEp*), which negatively affect floral organ size by decreasing both cell number and cell size [29, 32, 33], showed the highest expression in bracts and relatively low expression in petals and sepals (Fig. 3E). We also searched for these genes in the floral bud transcriptome data of *R. cantleyi*, which produces one of the largest flowers in the world [34]. The transcriptome data represented three different developmental stages of flowers (from small to large flower bud). Expression levels of genes involved in promoting petal cell proliferation and expansion significantly increased with the continued differentiation of flower buds, and were considerably higher than those of genes involved in inhibiting these two processes (Fig. 3D, E).

**Fig. 5:**
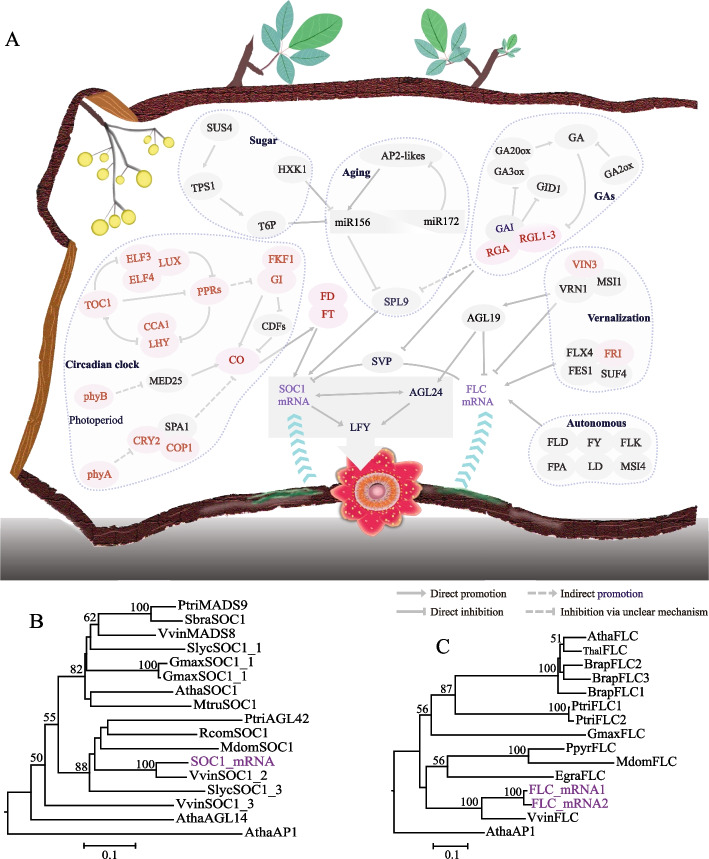
Genes involved in the regulation of flowering time in Sapria himalayana. **A** Simplified gene network regulating flowering time. Lost homologous genes in the S. himalayana genome are highlighted in red, and host-derived mRNAs, which expressed in bracts of S. himalayana, are indicated in purple. The gene network of regulating flowering time was summarized in Arabidopsis thaliana. The grey box represented the expression of any of the three genes (SOC1, AGL24, and LFY) could promote flowering. Blue stacked arrow represent the transfer path from host to S. himalayana. Yellow solid circles represent fruits of Tetrastigma, brown bark-like frame on the left and top side resembles the vine of Tetrastigma, and the below portion represents the roots of Tetrastigma, where S. himalayana parasites on it. **B** Gene tree of SOC1. **C** Gene tree of FLC. Species names are abbreviated as follows: A. thaliana (Atha), A. lyrata subsp. lyrata (Alyr), Brassica rapa subsp. pekinensis (Brap), Eucalyptus grandis (Egra), Glycine max (Gmax), Malus domestica (Mdom), Medicago truncatula (Mtru), Populus trichocarpa (Ptri), Pyrus pyrifolia (Ppyr), Ricinus communis (Rcom), Salix brachista (Sbra), Solanum lycopersicum (Slyc), Thellungiella halophila (Thal), Vitis vinifera (Vvin)

Upon sensing environmental changes, plants produce ‘florigen’, which moves through the phloem to the shoot apical meristem (SAM), where it induces flowering [35]. However, because *S. himalayana* is an endophyte with greatly reduced vegetative organs, the mechanism of flowering in *S. himalayana* is enshrouded in mystery. Homologous genes involved in multiple flowering pathways were comprehensively evaluated in the *S. himalayana* genome to illustrate the mechanism underlying flowering time (Additional file 2: Table S16). Our examination revealed that genes involved in circadian clock and photoperiod pathways had been completely lost (Fig. 5A; Additional file 2: Table S16). In addition, central integrators in the gibberellin (GA) pathway, including DELLA protein-encoding genes (*GAI* and *RGL1–3*), together with *CONSTANS* (*CO*), *FT*, *FLOWERING LOCUS C* (*FLC*), and *SUPPRESSOR OF OVEREXPRESSION OF CONSTANS 1* (*SOC1*), had also been lost (Fig. 5A; Additional file 2: Table S16). By contrast, the autonomous and sugar pathway genes were present (Fig. 5A; Additional file 2: Table S16). Interestingly, *FLC* and *SOC1* transcripts were detected in *S. himalayana* bracts (Additional file 1: Fig. S6). The *FLC* and *SOC1* genes of *S. himalayana* clustered with those of *Vitis* species but not with those of Malpighiales species (Fig. 5B, C). We speculate that *S. himalayana* perceives host mRNA signals to fulfill its reproductive process, which indicates adaptive evolution despite extensive gene loss.

### Plastid-based biosynthesis pathway

**Fig. 6:**
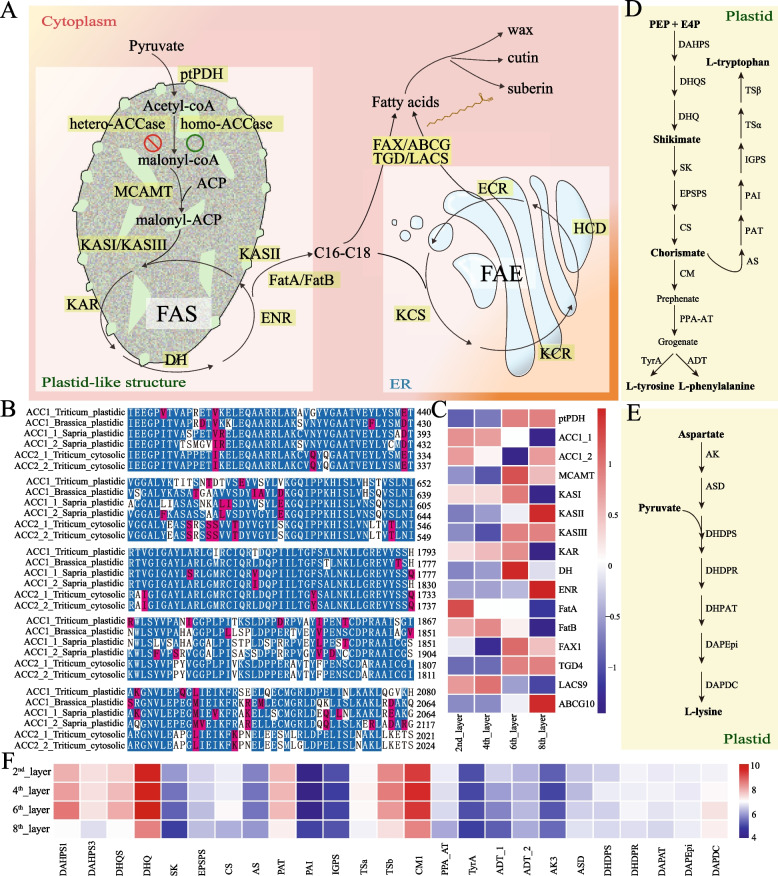
Overview of genes involved in the biosynthesis pathway of fatty acid and amino acid. **A** Simplified pathway of fatty acid biosynthesis, elongation and transport. **B** Alignment of the partial peptide sequences of homoACCases from Triticum aestivum, Brassica napus, and Sapria himalayana. Identical amino acids are underlayed with same color intensities. Genebank accession numbers of ACC1, ACC2_1, ACC2_2 of T. aestivum were T06161, Q41511, Q41525, and ACC1 accession number of B. napus was X77576. **C** Heatmap showing the z-score transformed gene expression data on fatty acid biosynthesis. **D**, **E** The biosynthesis pathway of aromatic amino acids and lysine in plant plastid, respectively. The key enzymes in each pathway are shown to the right of the arrows, which are all nuclear-encoded plastid-localized proteins. **F** Heatmap showing the log2-transformed gene expression data on amino acid biosynthesis. 2th, 4th, 6th, and 8th_layers indicate transverse layers of flower buds of S. himalayana. The full name of each gene was listed in Table S17

FAs, AAAs, and lysine, which are essential for growth and development, are de novo synthesized in the plastid [9–11]. Whether the loss of the plastid genome in *S. himalayana* [6, 7] affects the synthesis of FAs, AAAs, and lysine remains unknown. Acetyl-CoA carboxylase (ACCase, EC.6.4.1.2) catalyzes the first committed step in the FA biosynthesis pathway, converting acetyl-CoA to malonyl-CoA (Fig. 6A). Two distinct forms of ACCase, a multisubunit ACCase (heteroACCase) and a multifunctional ACCase (homoACCase), have been identified in plants [36]. HeteroACCase comprises four subunits, three of which (*accA*, *accB*, and *accC*) are encoded by nuclear genes, and the fourth (accD) is encoded by a plastid gene. HomoACCase is encoded by a single nuclear gene (*ACC*) [37]. In most plant species, heteroACCase localizes to plastids and homoACCase resides in the cytosol; however, in Gramineae and Brassicaceae, homoACCase is found in both compartments, and plastidic homoACCase replaces heteroACCase in the FA biosynthesis pathway in Gramineae [9, 36, 38]. The *accD* gene was not found in the nuclear or mitochondrial genome of *S. himalayana*, which lacks the plastid genome [6]. Three nuclear genes encoding heteromeric ACCase subunits were also absent in the *S. himalayana* genome; instead, two homoACCase-encoding genes were identified (*ACC1_1* and *ACC1_2*) (Additional file 1: Fig. S7). In *Triticum aestivum*, comparison of plastidic and cytosolic homoACCases revealed amino acid substitutions [9]. The amino acid sequence of *S. himalayana* homoACCase was similar to that of plastidic homoACCase of *T. aestivum* and *Brassica napus* (Fig. 6B), suggesting that *S. himalayana* homoACCases are plastidic types. Moreover, genes related to FA biosynthesis, elongation, and transport were expressed in flower buds (Fig. 6C). Genes involved in the biosynthesis pathway of AAAs and lysine were expressed in the flower bud of *S. himalayana* (Fig. 6D–F). Rafflesiaceae plants have lost the plastid genome, but plastid-like structures remain in *Rafflesia* species [7]. Taken together, we speculate that genome-less plastid-like structures continue to perform important metabolic activities, such as FA and amino acid biosynthesis, in *S. himalayana* (Fig. 6).

### HGT events

We identified at least 98 HGT events in the nuclear genome of *S. himalayana* that originated in its host *Tetrastigma* spp. Phylogenetic analysis revealed that all of these HGTs clustered with the genus *Vitis* (grouped with *Tetrastigma* in Vitaceae family), and not with Malpighiales clade, such as *M. esculenta* and *P. trichocarpa* (Fig. 4; Additional file 1: Fig. S8; Additional file 2: Table S18). Moreover, 72 HGTs contained 219 introns (≥ 100 bp), 34.3% of which showed the highest sequence similarity with *Vitis* intron sequences (Additional file 1: Fig. S9; Additional file 2: Table S19). After comparing the CDS structure of representative HGT orthogroups among donor, recipient, and relative species, we found that DNA- and mRNA-mediated HGT events co-existed in *S. himalayana* (Additional file 1: Fig. S10). A previous study deemed that DNA-mediated HGT events would more often result in functional transfers than mRNA-mediated HGTs [39]. However, we found no significant difference between the number of expressed HGTs without introns and HGTs with introns in *S. himalayana* flower buds (*P* = 0.55) (Additional file 2: Table S20). By characterizing the genomic structures of 98 HGTs, horizontally transferred genes in the donor(s) (here using *V. vinifera* as proxy), and their counterparts in *P. trichocarpa*, and vertically transmitted genes in *S. himalayana*, we found that protein-coding sequences in *S. himalayana*, especially those acquired via HGT, were compact; however, the introns in vertically inherited genes in *S. himalayana* were longer than those in other genes (Additional file 1: Fig. S11).

All HGTs were subjected to a functional enrichment analysis on the basis of MapMan categories [40], and the results displayed a high proportion of functional preference toward the categories “stress”, “secondary metabolism”, “RNA”, and “signaling” (Additional file 2: Table S21). Gene Ontology (GO) enrichment analysis [41, 42] indicated that HGT candidates enriched in the molecular function category were mainly involved in ADP binding and substance metabolism such as polysaccharide binding, nucleoside phosphate, oxidoreductase, chemokine receptor, chlorophyllase activity, and carbon–oxygen lyase activity (Additional file 1: Fig. S12), whereas those enriched in the biological process category were related to pigment catabolic process, multi-organism reproductive, organonitrogen compound catabolic process, and ossification (Additional file 1: Fig. S12). Specifically, genes encoding the regulators of cell wall development were identified as HGTs, including *HXXXD-type acyl-transferase family protein* (Fig. 4B), *FRUCTOKINASE3* (*FRK3*), and *CELLULOSE SYNTHASE LIKE E1* (*CSLE1*) (Additional file 1: Fig. S8 I, III). A gene encoding a peroxidase protein was identified as an HGT (Additional file 1: Fig. S8 XXI), which is exclusively expressed in the intrusive cells associated with host invasion in *Striga hermonthica* [43]. *ALTERED TRYPTOPHAN REGULATION 4* (*ATR4*), which is involved in defense response, underwent duplications after transfer from the host, resulting in three copies in *S. himalayana* (Fig. 4C). Some *S. himalayana* genes encoding receptor-like kinases (RLKs), receptor-like proteins (RLPs), and nucleotide-binding site–leucine-rich repeat (NBS–LRR) proteins, which participate in the plant immune system [44], were derived as HGTs from the host (Additional file 1: Fig. S8 II, XIV, XXXIV, XLII). Additionally, three aldehyde oxidase genes, *AAO3_1*, *AAO3_2*, and *AAO4*, which are required for the biosynthesis of the disease resistance hormone salicylic acid (SA) and that of many secondary metabolites (Additional file 2: Table S22), were derived as HGTs from the host (Fig. 4D).

Next, we investigated whether the HGTs were functional and expressed in different floral organs by evaluating the signatures of adaptive or purifying selection. A large number of HGT sequences (77 genes) was under purifying selection similar to the background (Additional file 2: Tables S23, S24). Two *S. himalayana* HGT lineages (seven genes) displayed positive selection (non-synonymous substitutions / synonymous substitutions [dN/dS] > 1) (Additional file 2: Table S23). The RELAX test indicated that a major proportion of HGT sequences and background genes (34 lineages) were under the same levels of selection (Additional file 2: Table S25), and some HGT sequences and background genes (15 lineages) were under intense selection (Additional file 2: Table S25). Out of 98 HGTs, 68 were expressed (fragments per kilobase per million mapped [FPKM] > 1) in at least one floral organ, suggesting that most HGT genes were actively transcribed and played vital roles in the development and defense response of *S. himalayana* plants (Additional file 2: Tables S23, S26).

### *S. himalayana* mitogenome

**Fig. 7:**
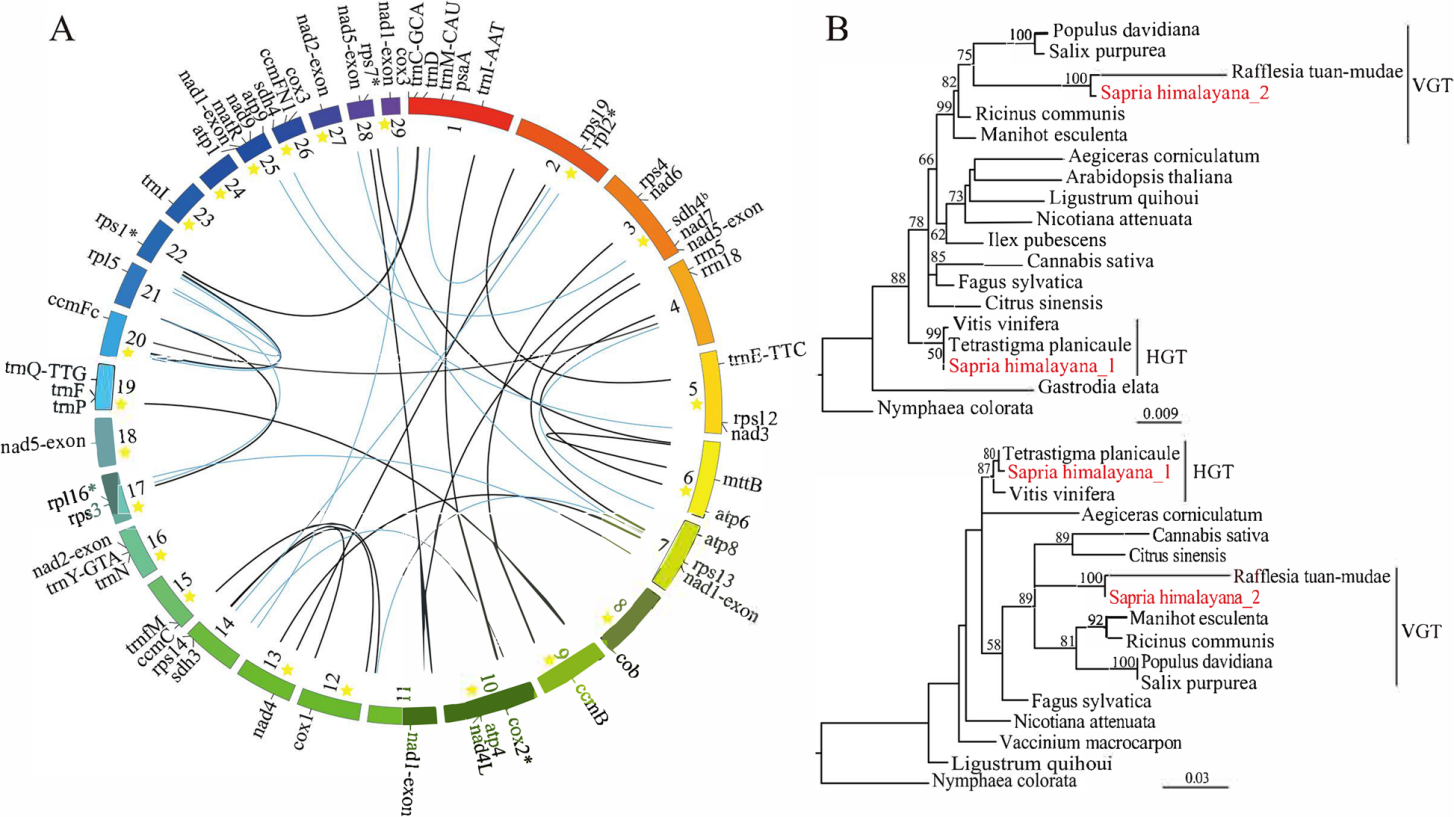
Mitochondrial genome draft and mitochondrial HGT in Sapria himalayana. **A** Draft of the S. himalayana mitochondrial genome. Twenty-nine contigs are manually displayed as a circle, including 21 circular contigs (ornamented with stars) and eight linear contigs. Suffixes ‘b’ and ‘*’ indicate duplicated genes and pseudogenes, respectively. Black lines in the middle represent repeat fragments of 200—500 bp in length, and blue lines represent repeat fragments longer than 500 bp. **B** Mitochondrial HGTs, cox3 (top) and sdh4 (bottom). HGT events are highlighted in red

The 645 kb mitogenome of *S. himalayana* was assembled as 21 circular and 8 linear contigs (Fig. 7A). The largest and shortest contigs were 42,542 and 7,340 bp in length, respectively. In total, 38 protein-coding genes were annotated (Additional file 2: Table S27), of which 13 showed clear phylogenetic clustering with Vitaceae family members (*T. planicaule* and *V. vinifera*) or with other distant related plant species instead of with the more closely related Malpighiales members (Fig. 7B; Additional file 1: Fig. S13). In total, one-third of the mitochondrial CDS were derived as HGTs, replacing their counterparts in *S. himalayana* (Additional file 1: Fig. S14). Two copies of *sdh4* were detected in *S. himalayana*, one of which clustered with *T. planicaule* and *V. vinifera* and the other with *R. tuan-mudae* (Fig. 7B). Ribosomal RNA genes *rpl10*, *rps2*, *rps10*, and *rps11* were absent in the mitochondrial genome (Additional file 1: Fig. S14), and were not detected in the nuclear genome. Five genes, including *cox2*, *rpl2*, *rpl16*, *rps1*, and *rps7*, were pseudogenes (Fig. 7A), among which *rps1* and r*ps7* were acquired by HGT (Additional file 1: Fig. S14).

### Host recognition and haustorium formation

Obligate parasitic plants germinate after sensing plants hormones, such as strigolactones (SLs) and karrikins (KARs), exuded by host roots. Five *KARRIKIN INSENSITIVE 2* (*KAI2*) genes, ancestral paralogs of *DWARF 14* (*D14*), were identified in *S. himalayana*. All *KAI2* genes clustered in the conserved *KAI2c* subclade (Additional file 1: Fig. S15). Compared with other species, *S. himalayana* harbored the most copies of *KAI2c* (Additional file 1: Fig. S15), which probably plays a vital role in host recognition. *KAI2c* genes possibly recognize an endogenous KAI2 ligand (KL) [45, 46]. This result could provide insights into the germination of Rafflesiaceae seeds.

Haustorium directly connects with the vascular system of the host to acquire nutrients and water. A previous study on *Striga* suggested that recruitment of the lateral root development (LRD) program in the parasitic plant and host induces the formation of the haustorium [43]. *AUX1*, *ARF19*, *LIKE AUX 3* (*LAX3*), *LOB DOMAIN-CONTAINING PROTEIN 18* (*LBD18*), and *SHORT HYPOCOTYL 2* (*SHY2*), which are master regulators of LRD [47], were present in the *S. himalayana* genome (Additional file 2: Table S12). *MP/ARF5* controls lateral root organogenesis and belongs to the LRD program [47]. It is worth mentioning that the base part of bracts in *S. himalayana* is fused to the host tissue, forming a chimeric structure known as a cupule, which is part of the haustorium.

## Discussion

### Improved genome assembly

The highly abundant repeat sequences in the *S. himalayana* genome posed a challenge to the acquisition of a contiguous, complete, and high-quality genome assembly. Using Illumina short reads (10X Genomics; 162.5 Gb, 64X) and Nanopore long reads (36.5 Gb, 14X), Cai et al. [6] assembled ~ 1.28 Gb of the *S. himalayana* genome, which accounted for approximately 40% of its estimated size (Table 1) [6]. In this study, we used PacBio long reads (353.5 Gb, 125X), 10X Genomics reads (301.8 Gb, 107X), and Illumina short reads (191.3 Gb, 68X) to improve the assembly quality. Transcriptome data generated from bracts, sepals, petals, and reproductive disk were used to annotate the genome (63.2 Gb). In total, 1.92 Gb of nuclear sequences (representing 70% of the estimated *S. himalayana* genome) were assembled (Table 1). Based on conserved embryophytic, eukaryotic, and eudicot datasets, the result of BUSCO analysis showed that the degree of completeness of genome assembly in our study was better than that reported by Cai et al. [6] (Additional file 2: Table S2). Besides, using the calN50 software, we also calculated the NG50 value of the two *S. himalayana* genomes, and found that the NG50 value determined in the current study was better than that determined by Cai et al. [6] (Table1). TE sequences were masked before gene annotation in most published genomes [48–50] and in the genome assembly reported in the current study, but this approach was not mentioned by Cai et al. [6]. Approximately 82.6% of validated genes (i.e., approximately 42,512 authentic genes) in Cai et al. [6] contained TE-like domains [6]. In this study, 13,670 genes were annotated with high confidence (Table 1). Based on the genome annotation files generated in this study and previously [6], we discovered 7,892 one-to-one homologs between the two genomes through synteny ortholog analysis using the JCVI [51]. The number of one-to-one homologs was roughly consistent with that of excluded genes (harboring TE-like domains) in Cai et al. [6]. Using the transcriptome data of bracts, sepals, petals, and reproductive disk generated in this study, we assessed the expression level of genes annotated in Cai et al. [6]. While only 20.1% of the genes predicted in Cai et al. [6] were expressed in floral organs, 75.2% of the genes annotated in our study were expressed in the corresponding organs (Additional file 1: Fig. S16). In addition, the characteristics of protein-coding genes identified in the *S. himalayana* genome in our study were similar to those of genes identified in related species (Additional file 2: Table S4). In comparison with five representative species, *S. himalayana* lacked 3,518 orthogroups (Fig. 2A), consistent with its extraordinary reduction in plant body size. Overall, this study provides a more complete, high-quality, and well-annotated genome of *S. himalayana* compared with previous studies.

### Large genes with long introns are functional

In this study, 53.1% of all *S. himalayana* genes carried long introns (≥ 1 kb) (Additional file 1: Fig. S17), and 23 of these genes contained introns longer than 100 kb. Among the top 1,000 genes with the highest expression levels in *S. himalayana* flower, 640 genes (64%) harbored long introns (Additional file 2: Table S28). Additionally, of the 122 genes required for floral development, flowering, haustorium formation, and FA, sugar, and amino acid biosynthesis and transport, 74 genes (60.7%) possessed long introns (Additional file 2: Table S29). This unique gene space morphology was also reported in *S. himalayana*, *Ceratopteris richardii*, and *Pinus tabuliformis* genomes [6, 52, 53], and a positive correlation between intron length and gene expression level was detected in *A. thaliana*, *Oryza sativa*, and *P. tabuliformis*, based on whole-genome analysis [53, 54]. Niu et al. [53] suggested that DNA methylation plays a key role in the accurate differentiation of small exons from super-long introns in conifers. Ultra-long introns were identified in 74 *MADS-box* genes of *P. tabuliformis*, 51 of which showed high expression levels [53]. Likely, long intron is benefical for increasing tolerance to gene mutations, and long-intron genes with high expression levels gain the tendency of evolving new exons (alternative splicing), and possess more regulatory elements. Thus, genes with long introns could be accurately identified and were transcribed in a large genome, and play important roles in plant development and adaptation.

### Floral organ development and flowering

The beauty and gigantism of Rafflesiaceae flowers have attracted considerable research interest for centuries. Floral gigantism helps trap prey, facilitates thermal regulation, and mimics rotting animal carcasses to attract carrion flies [55, 56]. However, the network that regulates the development of giant flowers remains to be illustrated. Genes involved in floral organ specification were identified in our study, and their expression patterns were almost consistent with those reported by Nikolov et al. [57]. *TM6* was expressed in several floral organs but showed especially strong expression in petals and reproductive disk, similar to the expression pattern of *CluTM6* in *Clutia* (Peraceae), a close relative of Rafflesiaceae [57]. The adaxial epidermis of petals in *S. himalayana* had papillate cells [58], which are often used as a marker of petal cell identity [59, 60]. Based on the expression patterns of *TM6* and *PI*, we speculate that the inner perianth lobes are indeed petals in *S. himalayana*. In *Rafflesia*, *RfPI* is strongly expressed in the diaphragm and perianth tube, which are derived from the petal whorl [57], and similar papillate cells were found in the adaxial epidermis of perigone lobes and diaphragm in *Rafflesia patma* [61]. Besides, *RfTM6* was expressed in all floral organs in *Rafflesia* [57]. Together, these findings suggest that perianth lobes of *Rafflesia* flowers might retain some features of petals.

Multiple genes contribute to flower size in *Arabidopsis* (Fig. 3D). Based on the flower bud transcriptome of *S. himalayana* and *R. cantleyi*, we speculate that *MP*, *ANT*, *GIF1*, *SWP*, and *KLUH* genes are important regulators of flower development and contribute to floral gigantism in Rafflesiaceae (Fig. 3D, E). Although *BPEp* negatively affects floral organ size [62], it was also expressed at the perigone lobe of *R. cantleyi*, which is suggestive of its involvement in flower development in Rafflesiaceae. TFs related to flower development, including stem cell activity, auxin response, flower or root hair development, anthocyanin accumulation, abaxial/adaxial conversion, secondary metabolism, and biotic stress response [25, 63–66], were also uncovered in the species-specific dataset of *S. himalayana* (Additional file 2: Table S11), similar to the results obtained in the transcriptome analysis of the perigone lobe of *R. cantleyi* [62].

Flowering is a vital event in the lifecycle of flowering plants, and its timing is accurately controlled by endogenous factors (hormones, sugar, autonomous, and age) and exogenous cues (photoperiod and vernalization) that converge toward a few floral integrator genes, including *FT*, *SOC1*, *FLC*, *CO*, and *AGL24*, as well as floral meristem identity genes, such as *LEAFY* (*LFY*), *APETALA 1* (*AP1*), and *AGAMOUS* (*AG*) [67–70]. Although *FT*, *FLC*, and *SOC1* are absent in the *S. himalayana* genome, host-derived *FLC* and *SOC1* transcripts were detected in bracts, suggesting that *S. himalayana* perceives host flowering signals to regulate its own flowering time. The flowering time of *C. australis* is well synchronized with that of its host, because *Cuscuta* species utilize the host FT to regulate their own flowering [8]. However, the flowering time of *S. himalayana* (September to December) does not coincide with that of its host (April to June). This may be because the flowering time of *S. himalayana* is determined by its physiological nature. When *S. himalayana* recognizes the flowering signal from the blooming host, it transforms from uniseriate strands to multiseriate strands, further proliferating and eventually emerging from the host [3]. This developmental process requires an abundance of resources. Thus, *S. himalayana* takes a long time to accumulate sufficient energy, which delays the onset of flowering in *S. himalayana* compared with that in its host. *AGL24* promotes early flowering in a dosage-dependent manner [71, 72], as indicated by premature flowering in transgenic *Arabidopsis* overexpressing *AGL24*, and is involved in flowering in *R. cantleyi* [73]. In this study, we identified three copies of *AGL24* in the genome of *S. himalayana* (Fig. 3A), which enable early flowering in *S. himalayana* under limited energy conditions.

### *S. himalayana* plastids devoid of genome but capable of performing essential nonphotosynthetic functions

Based on the attempt to assemble the plastid genome in this study and in previous studies [6, 7], it seems that plastid genome has been lost in *S. himalayana*. However, plastid is responsible for the biosynthesis of many molecules/compounds essential for plant survival, including FAs, amino acids, nucleotides, heme, and hormones. In plants, AAAs are not only essential for protein synthesis but also serve as precursors of a wide range of secondary metabolites, such as indoles and aromatic substances, which play important roles in plant growth, reproduction, development, and defense [74, 75]. In this study, we found that genes involved in the biosynthesis of FA, AAAs, and lysine were expressed in *S. himalayana* flower tissues (Fig. 6C, F). Plastid-based metabolic functions also depend on nuclear-encoded plastid-localized proteins. Hundreds of putative nuclear-encoded plastid-targeting proteins, which mainly participate in amino acid metabolism and transport, carbohydrate and nucleotide metabolism, and tetrapyrrole and isoprenoid biosynthesis, were present in *S. himalayana* (Additional file 1: Fig. S18). Tetrapyrole and isoprenoid biosynthesis pathways are related to the synthesis of heme and carotenoids, respectively [76, 77]. Ng et al. [78] reported that *R. cantleyi* retained nuclear-encoded plastid-targeting genes involved in some plastid-associated processes such as the biosynthesis of amino acids, lipids, and heme. Thus, we speculate that Rafflesiaceae plants represent a plastid-bearing lineage, without a plastid genome, that is capable of performing plastid-based metabolic functions, such as the biosynthesis of FAs, amino acids, heme, and plant hormones. Similar results were reported in *Polymella* spp*.*, which are free-living, nonphotosynthetic green algae [79].

### Convergent functional HGT

HGT not only promotes genome evolution but also increases plant adaptation [8, 80, 81]. Massive mitochondrial and nuclear HGTs have been reported in Rafflesiaceae [6, 82]; however, there were some differences in the HGT dataset between Cai et al. [6] and this study, which might be caused by differences in the sequencing technologies used. Cai et al. [6] detected 568 horizontally transferred nuclear genes and pseudogenes belonging to 81 orthogroups in *S. himalayana*, and found that approximately 10 shared HGTs in Rafflesiaceae encode proteins related to defense or stress response. We also found that HGTs encoded proteins related to host invasion, floral development, and defense response. Based on the annotation of HGT homologs in *S. himalayana*, *Cuscuta* [83], and Orobanchaceae [39], we found that some annotation categories were identical among the three species (Table 2), which suggests convergent HGT in parasitic plants. In addition, these HGTs were mainly expressed at the parasite–host interface, including prehaustoria and haustoria of *Cuscuta* and Orobanchaceae, respectively, and in the bracts of *S. himalayana*, which are intimately connected at the base with host root tissues (Fig. 1C; Table 2; Additional file 2: S30), suggesting that these genes are likely involved in the negotiation between parasite and host, and in regulating the transport of nutrients and genetic materials [43, 84]. Besides, host-derived mRNAs were also found in *S. himalayana* bracts (Additional file 1: Fig. S6), indicating that horizontal transfers of host genes and mRNAs are common in vegetative tissues and early-developmental stage floral organs. Thus, using host genetic codes not only keeps the endoparasite from being detected but also serves as an energy-efficient strategy for the endoparasite, improving its adaptation and chance of survival. We propose that *S. himalayana* borrows the host genes not only to complete its lifecycle but also to disguise itself from being rejected by the host.

**Table 2:** Shared HGT functions based on homologous annotation among *Sapria himalayana*, *Cuscuta*, and Orobanchaceae

Function category	*S. himalayana*	*Cuscuta*	Orobanchaceae
Cytochrome P450	√	√	√
LRR-containing protein	√	√	√
ABC transporter C family member	√	√	√
Nuclear pore complex protein	√	**-**	√
Protein trichome birefringence	√	√	**-**
HXXXD-type acyl-transferase family protein	√	√	**-**
G-type lectin S-receptor-like serine/threonine-protein kinase	√	√	**-**
Werner syndrome -like exonuclease	√	√	**-**
ATP-dependent DNA helicase	√	√	**-**
Auxin response factor	√	√	**-**

The annotation informations of HGTs in *Cuscuta* and Orobanchaceae were reported in Yang et al. [83] and Yang et al. [39], respectively

### Host–parasite interaction

Little is known about how Rafflesiaceae seeds germinate and how endophytic cells enter the host. Apart from multiple locals claiming to have successfully reached flowering in *Rafflesia* plants grown from seeds [85–87], there was no success in vitro [85], suggesting that *Rafflesia* plants require unknown factor(s) released from the host to flower. Seeds of Orobanchaceae species depend on host-derived SLs to germinate, and most receptors for SLs are encoded by *KAI2d*, which underwent expansion in *S. asiatica* and *Orobanche cumana* genomes [88]. *KAI2d* evolved from the KAR receptor gene *KAI2i* by duplication [45]. In this study, no *KAI2d* gene and only five *KAI2c* genes were identified in the *S. himalayana* genome (Additional file 1: Fig. S15). Moreover, *S. himalayana* could detect KL, indicating that *S. himalayana* seeds depend on stimulants probably different from those required by Orobanchaceae seeds to germinate. de Vega et al. [89] reported that mycorrhizae are likely associated with *Cytinus hypocistis* (Cytinaceae), an endoparasitic plant, and the host, forming a tripartite association, and speculated that *Cytinus* could recognize both host exudates and fungal molecules as germination cues. The molecular clues mined from the *S. himalayana* genome could provide insights for further exploration.

Cupule in Rafflesiaceae plants functions as a haustorium, providing nutrients and water [90, 91]. Several histoanatomical studies described the process of teardrop-shaped protocorm penetration into the host for flowering in endophytes, and found that the haustorium of Rafflesiaceae plants starts to form in the vascular cambium of the host [3, 61, 87, 92]; however, the molecular mechanism of haustorium formation is unknown. The process of haustorium formation has been well studied in Orobanchaceae, and is regulated by the LRD program genes [43, 88]. Master regulators of LRD genes were identified in *S. himalayana.* It appears that these parasitic species use the same mechanism of haustoria development to attach to and penetrate the host.

## Conclusions

In this study, we de novo assembled the genome of *S. himalayana*. Our results showed that despite its large genome, the proteome of *S. himalayana* is the smallest among all sequenced angiosperms. Comparative genomic analyses revealed remarkable gene loss in *S. himalayana*, consistent with its reduced vegetative body. HGT events are common among endoparasitic plants and play an important role in their lifestyle adaptation. Genes involved in floral development, flowering, and FA and AAA biosynthesis were identified in the *S. himalayana* genome, and these genes were likely responsible for its unique body plan and special lifestyle. Our results will be of great value to future studies on the development, lifestyle, ecological adaptation, and plant–plant interactions of endoparasites.

## Methods

### Plant materials, DNA extraction, and sequencing

Fresh male flower buds of *S. himalayana* were collected in the tropical ravine rainforest of Yunnan Province, China. High-quality genomic DNA was extracted from the flower buds using DNAsecure Plant Kit. The concentration and purity of the extracted DNA were assessed using Nanodrop 2000 spectrophotometer (IMPLEN, Westlake Village, CA, USA) and Qubit 2.0 Fluorometer, and the integrity of the extracted DNA was measured using pulsed-field electrophoresis on 1.0% agarose gel. To perform long-read sequencing, a 20 kb insert size library was prepared and sequenced on the PacBio Sequel platform, which generated 353.5 Gb raw data for the assembly. To construct the 10X Genomics library, approximately 1 ng of input DNA (average length = 50 kb) was used for the GEM reaction procedure during PCR, and 16-bp barcodes were introduced into droplets. The droplets were then fractured, and the DNA library was purified and sequenced on the Illumina NovaSeq 6000 platform. To perform short-read sequencing, a paired-end library (insert size =  ~ 350 bp) was constructed and sequenced using on the Illumina NovaSeq 6000 platform. All libraries were sequenced at Novogene Technologies Corporation, Beijing, China.

### Genome sequence, assembly, and annotation

The genome sequence of *S. himalayana* was assembled using three sequencing methods: single molecule real-time sequencing on the PacBio Sequel platform, 10X Genomics sequencing, and Illumina paired-end sequencing. Firstly, the PacBio SMRT long reads were de novo assembled using the FALCON assembler [8]. Before assembly, the PacBio reads were corrected using FALCON and then assembled into contigs using default parameters [48, 49]. The resulting contigs were polished using Quiver [93] by aligning the SMRT reads. Secondly, paired-end Illumina short reads were used to perform the second round of error correction with Pilon [9]. Thirdly, the BWA-MEM algorithm [94] was applied to align the 10X Genomics data to a scaffold using default settings. Additionally, FragScaff (v1.1) [95] was used to generate super-scaffolds using the barcoded sequence reads.

A combination of three methods, including homology-based prediction, ab initio prediction, and transcriptome-assisted prediction, was used to identify protein-coding genes. Homology-based predictions were performed using the amino acid sequences of protein-coding genes of eight plant species, including *A. thaliana*, *C. australis*, *Hevea beasiliensis*, *Linum usitatissimum*,* O. sativa*, *P. trichocarpa*, *Ricinus communis*, and *V. vinifera*. To identify homologs, a TBLASTN search was conducted against the *S. himalayana* genome assembly, with an *E*-value cutoff of 10^–5^. Genewise (v2.4.1) [96] was used to predict gene models using alignments of amino acid sequences of S. himalayana and the above-mentioned eight plant species. The ab initio prediction was performed using Augustus (v3.2.3) [97], GlimmerHMM (v3.04) [98], SNAP (v2013.11.29) [99], Geneid (v1.4) [100], and Genescan (v1.0) [101], with default parameters. Transcriptome-assisted prediction was performed using RNA extracted from four floral organs (bract, sepal, petal, and reproductive disk) and sequenced on the Illumina NovaSeq 6000 platform. Two methods were used to fulfill RNA-Seq-based predictions: (1) mapping the filtered RNA-Seq raw reads to the *S. himalayana* genome assembly generated in this study, and then assembling the transcripts using TopHat (v2.0.11) [102] and Cufflinks (v2.2.1) [103] with default parameters; and (2) assembling the RNA-Seq data using Trinity (v2.1.1) [104], and improving gene structures using Program to Assemble Spliced Alignments (PASA) [105]. Genes predicted by the above three methods were merged with EVidenceModeler (EVM; v1.1.1) [105] to obtain a non-redundant reference gene set, which was updated again using PASA [105].

Protein-coding genes were functionally annotated according to their best matches identified using BLASTP searches against Swiss-Prot [106] and National Center for Biotechnology Information (NCBI) non-redundant (Nr) protein databases (*E*-value ≤ 1e^−5^). Motifs and domains were annotated using InterProScan (v5.31) [107] by searching against InterPro databases, including Pfam, PRINTS, PROSITE, ProDom, and SMART.

The enriched GO functional categories in the HGT dataset were displayed using the web-based ReviGO software [42]. The MapMan categories of lost, duplicated, and species-specific genes in the genome of *S. himalayana* were determined using the Mercator web tool [40]. Fisher’s exact test in R (v3.6.2) was used to test for the enrichment of functional categories.

### Repeat sequence and ncRNA annotation

Two complementary methods (homology alignment and de novo annotation) were used to identify repeats in the *S. himalayana* genome sequence. The homology-based repeat library was generated from the Repbase database (http://www.girinst.org/repbase) using RepeatMasker (v4.0.5) [108]. The de novo annotation-based repeat library was generated using RepeatModeler (v1.0.3), RepeatScout (v1.0.5) [109], and LTR_FINDER (v1.0.7) [110], and further predicted by RepeatMasker (v4.0.5). The de novo prediction of tandem repeats was performed using the Tandem Repeats Finder (TRF) software (v4.09) [111]. Additionally, RNAs were predicted using the tRNAscan-SE software [112], whereas rRNAs, miRNAs, and snRNAs were identified through searches against the Rfam database (13.0) [113] using INFERNAL (v1.1.2) [114].

### RNA-Sequencing (RNA-Seq) and Isoform-Sequencing (Iso-Seq)

To capture as many transcripts as possible, two versions of the *Sapria* transcriptome were assembled: one using RNA-seq and another using Iso-seq and RNA-seq. To perform RNA-seq, four different floral tissues were used, including bracts, sepals, petals, and reproductive disk; however, given the limited availability of *S. himalayana* floral tissues, no biological replicates could be performed. The freshly harvested tissues were stored in RNAlater (Ambion, Life Technologies, Austin, TX, USA), flash frozen in liquid nitrogen, and then stored at -80 °C. Total RNA was extracted from each sample using RNA prep pure Plant RNA Purification Kit (Tiangen Technologies, CA, USA). The integrity of RNA was evaluated by electrophoresis on 1.0% agarose gel, followed by staining with ethidium bromide. Additionally, the quality and quantity of RNA were assessed using Agilent 2100 Bioanalyzer, Qubit 2.0 Fluorometer, and Nanodrop 2000 spectrophotometer (IMPLEN, Westlake Village, CA, USA). The cDNA library was constructed using the NEBNext Ultra RNA Library Prep Kit (NEB, Ipswich, MA, USA), following the manufacturer’s recommendations. Library preparations were sequenced on an Illumina NovaSeq 6000 platform, and 191.29 Gb of 150-bp paired-end reads were obtained by Beijing Novogene Bioinformatics Technology Co., Ltd. (Beijing, China) for gene annotations and gene expression analysis. Raw data in fastq format were first processed using in-house Perl scripts. The raw reads were filtered by removing adapter sequences, reads containing poly-N sequences, and low-quality sequences. Clean reads of each sample were separately mapped to the *S. himalayana* reference genome sequence using hisat2 (https://github.com/DaehwanKimLab/hisat2), and the mapped reads were counted with the Rsubread package of R to determine the number of FPKM [115]. Because the intimate physical connection between *S. himalayana* and its host increases the probability of horizontally transferred mRNAs, we assembled the reference transcriptome of *S. himalayana*. Clean reads were de novo assembled into transcripts using Trinity (v2.1.1) [104]. Then, redundant transcript sequences were removed using the CD-HIT tool [116], and protein-coding genes were predicted based on non-redundant transcript sequences using the ANGEL software (https://github.com/PacificBiosciences/ANGEL).

Another version of the transcriptome sequence was obtained by performing Iso-seq and RNA-seq on four transverse layers of flower buds (~ 1 cm in diameter) (Additional file 1: Fig. S2), each with three biological replicates. To perform Iso-Seq, equal quantities of total RNA samples corresponding to each layer were mixed to generate a pooled sample for library construction. The cDNA library was constructed using the SMARTer PCR cDNA Synthesis Kit, according to the manufacturer’s recommendations, and then subjected to Iso-seq on the PacBio Sequel II platform (Novogene). Raw sequence reads were trimmed using the SMRT Link 5.0 software. Then, circular consensus sequences (CCSs) were produced from the subread BAM file using the following parameters: min_length 200, max_drop_fraction 0.8, no_polish TRUE, min_zscore -9999, min_passes 1, min_predicted_accuracy 0.8, max_length 18,000. Subsequently, the CCSs were classified into two categories, full-length non-chimeric (FLNC) and non-full-length (nFL) transcript sequences, depending on whether they contained the forward and reverse primer sequences and poly(A) tail. The FL sequences were then subjected to isoform-level clustering (ICE), followed by final Arrow polishing. The polished consensus sequences were further corrected using Illumina RNA-seq reads (described below) with the LoRDEC software. Redundant sequences were removed using the CD-HIT tool. Finally, 10,367 protein-coding genes were predicted using the ANGEL software (https://github.com/PacificBiosciences/ANGEL). The same steps involved in RNA-seq, including RNA extraction, quality verification, and sequencing, raw read filtering, mapping, and FPKM calculation, were performed as described above (for the first transcriptome). The similarity among biological replicates was evaluated by performing principal component analysis (PCA) using the prcomp function in R. Because bud_2_3 and bud_4_3 libraries did not cluster with the other replicates (Additional file 1: Fig. S19A), both these libraries were discarded, and PCA was repeated, resulting in a strong correlation among the biological replicates (Additional file 1: Fig. S19B). To determine gene expression levels in each sample, the average FPKM values of biological replicates were calculated.

Because of the precious nature and limited availability of *S. himalayana* materials in the wild, this study was limited to the transcriptome of flower buds at different developmental stages. The floral bud transcriptome data of *R. cantleyi* representing three different developmental stages [34] were downloaded from NCBI (NCBI accession: PRJNA378435) to facilitate the investigation of genes relating to floral development in *S. himalayana*. Methods relating to transcriptomic assembly pipeline, CDS prediction, and gene expression quantification followed that of *S. himalayana.*

### Identification of orthogroups and HGT events

OrthoFinder (v2.5.2) [117] was used to group the orthogroups of *S. himalayana* and 13 plant species, including *A. thaliana*, *Cucumis sativus*, *C. australis*, *Eucalyptus grandis*, *Glycine max*, *Gossypium raimendii*, *M. esculenta*, *Mimulus guttatus*, *O. sativa*, *Papaver somniferum* (NCBI accession: PRJNA435796), *P. trichocarpa*, *Quercus robur*, and *V. vinifera* (phytozome v12) (Additional file 2: Tables S31, S32). Prior to identifying the orthogroups, we first obtained proteins corresponding to primary transcripts in each of the above-mentioned plant species. HGTs were identified based on the following criteria: (1) length of protein sequence of the candidate HGT > 150 aa; (2) candidate HGT phylogenetically clustered with *Vitis* (grouped with *Tetrastigma* in the family Vitaceae), other than the Malpighiales species, such as *M. esculenta* and *P. trichocarpa*. Additionally, a candidate HGT showing expression in any flower tissue was recognized as a functional HGT.

### Identification of homologous genes

*S. himalayana* homologs of *A. thaliana* genes involved in the regulation of floral identity, floral development, floral size, flowering time, and FA, AAA, and lysine biosynthesis were identified through an all-by-all BLASTP, with *E*-value cutoff of 10^–5^. To resolve ambiguous results, phylogenetic analysis was performed as described below. The network of genes regulating petal size, flowering time, and FA, AAA, and lysine biosynthesis [9–11, 24, 68, 118] was obtained from *A. thaliana*. A number of *A. thaliana* orthologs involved in leaf and root organogenesis, nutrient uptake and transport, plant immunity, and symbiosis have been reported in previous studies [118, 119]. Likewise, corresponding homologous genes in *S. himalayana* were identified. Besides, all presumed nuclear-encoded plastid-targeting genes were identified in *Polytomella parva* [79]. Then, homologs of these genes were identified in *S. himalayana* through an all-by-all BLASTP.

### Phylogenetic analysis

Amino acid sequences corresponding to HGTs and the identified homologous genes were aligned using MAFFT (v7.471) [120], with default parameters. PAL2NAL [121] was then used to generate the corresponding CDS alignment, and trimAL [122] was used to remove ambiguous sites in the alignment with the parameter ‘-gt 0.6’. Finally, gene trees were inferred by IQ-TREE [123] with ultrafast bootstrap testing (1,000 replicates).

### Phylogenetic analysis of *MADS-box* genes

Information regarding the *MADS-box* gene family in *Arabidopsis* was retrieved form The Arabidopsis Information Resource (TAIR) database (https://www.arabidopsis.org/browse/genefamily/MADS). The SRF-TF domain (MADS-box, PF00319) model was downloaded from PFAM (http://pfam.xfam.org/), and used to screen MADS-box protein sequences in *A. thaliana*, *M. esculenta*, *P. trichocarpa*, *R. cantleyi*, and *S. himalayana*, using the hmmsearch program in the HMMER package (v3.2.1), with an *E*-value cutoff of 10^–5^. Amino acid sequences of all MADS-box proteins in the five above-mentioned plant species were aligned using the hmmalign program, combined with the SRF-TF domain model. Then, the amino acid sequence alignment was forced onto the CDS alignment using PAL2NAL. The remaining steps involved in the construction of the phylogenetic tree were performed as described above.

### Assembly and annotation of mitochondrial and plastid genomes of *S. himalayana*

PacBio reads (5 Gb) were used to de novo assemble the mitochondrial genome of *S. himalayana.* Prior to filtering and correcting the PacBio reads, the genomics reads were mapped on the mitochondrial genome of *R. lagascae* (NCBI accession: SRX434531) [7] using minimap2 (v2.11-r797) [124]. All mapped reads were extracted and further processed using SAMtools (v1.4) [125]. Next, Canu (v1.7) [126] (useGrid = false contigFilter = ‘2 1000 1.0 1.0 2’ corOutCoverage = 999 stopOnLowCoverage = 50 –p assembly –d assembly genomeSize = 1 m –pacbio-raw all.sam.filter.fasta) was used to generate the initial assembly. Contigs generated from the initial assembly were used as reference sequences for further iterative mapping and extension processes. Finally, the assembled contigs were calibrated against Illumina reads using Geneious (v 2020.2.2) (Biomatters, Inc., Auckland, New Zealand). In the process of genome assembly, contigs that assembled into loop repeats in a helical pattern using CANU were confirmed by Illumina reads using Geneious and then considered as a circular chromosome. Linear contigs were extended by Illumina reads using Geneious. When repeats appeared at both ends of the contig, extension continued, leading to an overlap of the two ends; such contigs were considered as circular chromosomes, while contigs with no terminal overlap were considered as linear chromosomes. The assembled contigs were annotated using the mitochondrial annotation database containing the gene annotations of 66 plant species (in-house collection). Then, the boundaries of each gene were confirmed manually. The loss of mitochondrial gene was confirmed based on whether the Illumina reads obtained from *S. himalayana* could be mapped to homologous sequences in other species. The sequencing depths of intact mitochondrial protein-coding genes were also calculated (Additional file 1: Fig. S20).

The plastid genome sequence of *S. himalayana* was assembled using VELVET [127], with *k*-mer values ranging from 37 to 45. In total, 5 Gb Illumina reads were used to de novo assemble the plastome of *S. himalayana.* The plastome sequences of *P. wilsonii* (NCBI accession: NC_037223.1) and *V. palmata* (NCBI accession: NC_039791) were used as references. However, because of very few plastid genome-specific Illumina short reads, the plastid genome could not be assembled.

### Identification of HGTs in the mitochondrial genome of *S. himalayana*

To identify HGTs in *S. himalayana* mitochondrial genome, the CDS of each mitochondrial gene was extracted from 19 representative species, including *Aegiceras corniculatum*, *A. thaliana*, *Cannabis sativa*, *Citrus sinensis*, *Fagus sylvatica*, *G. elata*, *Ilex pubescens*, *Ligustrum quihoui*, *M. esculenta*, *Nicotiana attenuate*, *Nymphaea colorata*, *P. davidiana*, *R. tuan-mudae*, *R. communis*, *Salix purpurea*, *S. himalayana*, *T. planicaule*, *Vaccinium macrocarpon*, and *V. vinifera*. The mitogenomes of all species, except *S. himalayana* and *T. planicaule* (Additional file 2: Table S33), were downloaded from the NCBI website (http://www.ncbi.nlm.nih.gov). The Illumina reads of *T. planicaule* were generated using the Illumina NovaSeq 6000 platform. Based on the mitochondrial genome of *V. vinifera*, the CDS set of *T. planicaule* was extracted using Geneious (Biomatters, Inc., Auckland, New Zealand). The identification of mitochondrial HGTs was analogous to that of nuclear HGTs.

### Selective constraint analyses

Branch-model tests in PAML [128] and the RELAX tool on the Datamonkey Adaptive Evolution Server (http://test.datamonkey.org/relax/) were employed to identify signatures of positive or purifying selection. In each HGT-based phylogenetic tree, the horizontally transferred genes were denoted on foreground branches, while all other genes were indicated on background branches. Using the branch-model tests in PAML, the value of dN/dS ratio (ω) was calculated, where ω < indicates purifying selection; ω = 1 indicates neutral evolution; and ω > 1 indicates positive selection. The RELAX test was used to determine whether selection was intensified or relaxed in the foreground branch compared with the background branch. A likelihood ratio test was conducted to evaluate whether selection varied between the foreground and background branches. A significant result (*P*-value < 0.05) indicates intensified selection if K > 1 and relaxed selection if K < 1; other conditions imply no difference between the foreground and background branches.

### Intron analyses

Intron number, total intron length, CDS length, and gene length were compared between *S. himalayana*, *V. vinifera*, and *P. trichocarpa*. Custom shell scripts were used to extract the number, length, and positions of introns from the genome annotation gtf files, and then BEDTools (v2.29.2) [129] was used to extract intron sequences from the genome sequence. Intron sequences starting with GT and ending with AG were suggestive of accurate splicing. Genes harboring at least one intron greater than 1 kb in length were designated as long-intron genes. To compare intron positions, the deduced amino acid sequences were aligned using MAFFT (v7.471), and then corresponding to the CDS alignment using PAL2NAL. To analyze sequence similarity between two introns, global alignment of introns with analogous positions was performed on the NCBI website. BLASTN searches of intron sequences (≥ 100 bp) were performed against the nucleotide (Nt) database of NCBI to retrieve the most similar sequences. Chisq.test was used to assess the significance of HGTs with and without introns.

## Supplementary Information

**Additional file 1: Fig. S1.** Evaluation of *Sapria himalayana* genome by k-mer analysis. X-axis shows the k-mer depth, and Y-axis depicts the k-mer frequency. The genome size of *S. himalayana* was estimated as 2,822.01 Mb. **Fig. S2.** Schematic diagram on stratified sampling from flower bud of *Sapria himalayana*. Tissues from the second, fourth, sixth, and eighth layers were used for RNA-seq and Iso-seq. The outermost host bark was removed prior to sampling. **Fig. S3.** Functional enrichment analysis of species-specific genes in *Sapria himalayana*. MapMan categories of significantly enriched genes are displayed using the logarithmic values of their *P*-values. **Fig. S4.** Phylogenetic tree of MADS-box gene family. Species names are abbreviated as follows: *Arabidopsis thaliana*, *Manihot esculenta*, *Populus trichocarpa*, *Rafflesia cantleyi*, and *Sapria himalayana*. Genes from *S. himalayana* and *R. cantleyi* are highlighted in red and brown colors, respectively. BS > 50% are shown. **Fig. S5.** Phylogenetic inference and sequence similarity of *TOMATO MADS-BOX GENE6*and *FRUITFUL-like*.Neighbor-joining tree of the newly isolated *euAP3* and *TM6*homologs.Neighbor-joining tree of *FRUITFUL-like*homologs in represent species. Information on gene sequences is provided in Table S14. **Fig. S6.** Heatmap showing the log_2_-tranformed expression profile of genes regulating flowering time. Host-derived mRNAs are highlighted in purple. **Fig. S7.** Phylogenetic tree of *acetyl-CoA carboxylase *. Species names are abbreviated as follows: *Arabidopsis thaliana *, *Cucumis sativus *, *Eucalyptus grandis *, *Glycine max *, *Gossypium raimendii *, *Manihot esculenta *, *Mimulus guttatus *, *Oryza sativa *, *Papaver somniferum *, *Populus trichocarpa *, *Quercus robur *,* Sapria himalayana *, and *Vitis vinifera *. **Fig. S8.** Horizontal gene transferevents in the nuclear genome of *Sapria himalayana*. Species names are abbreviated as follows: *Arabidopsis thaliana*, *Cucumis sativus*,* Cuscuta australis *, *Eucalyptus grandis*, *Glycine max*, *Gossypium raimendii*, *Manihot esculenta*, *Mimulus guttatus*, *Oryza sativa*, *Papaver somniferum*,* Populus trichocarpa *, *Quercus robur*, *S. himalayana*and *Vitis vinifera.* HGT events are highlighted in red. **Fig. S9.** Overview of intron sequences in HGTs.72 HGTs with at least one intron and 26 HGTs without intron.The average value of identity of intron sequence searched on nucleotidedatabase at NCBI website using BLASTN. 79introns from Vitaceae, with average identity was 84.67%, 16introns from other species, with average identity was 84.62%, and origins of other introns was uncertain. **Fig. S10.** Gene structure of selected introns in four sequences. Yellow and green bars represent coding sequences; the vertical dashed lines represent the intron position, the boxes represent introns; The value of sequence identity between introns enclosed in square bracket displayed on line. The total number of introns per coding sequence showed at the right of the bar box. D, genes in donor, substituted by *Vitis vinifera*; H, HGT genes in *Sapria himalayana*; V, vertical genes in *S. himalayana*; VR, related sequences of the vertical gene in *Populus trichocarpa*.The intron positions of three selected introns were highly conserved in D, H, VR sequences, but the V sequence was intronless.The intron positions of two selected introns were highly conserved in D, H, V, VR sequences, but the first intron position of H sequence had changed.the V sequence has been lost, and the H sequences was intronless. **Fig. S11.** Comparisons of characteristics among foreign genesin *Sapria himalayana *, Vertical genes in *S. himalayana *, genes in donor species, and genes in relative species.Average number of introns per gene.Average of total intron length per gene.Average CDS length per gene.Average gene length. **Fig. S12.** Scatterplot of enriched GO terms in the nuclear horizontal gene transferdataset of *Sapria himalayana*. MF, molecular function; BP, biological process. Bubble color indicates the *P*-value of the GO term; bubble size indicates the frequency of the GO term in the GOA database. **Fig. S13.** HGT eventsin the mitochondrial genome of *Sapria himalayana*. **Fig. S14.** Comparison of mitochondrial protein-coding gene content between *Sapria himalayana *and representative angiosperms. Colors indicate native, foreign, and absentgenes. The presence of both native and foreign copies of a gene is depicted by a subdivided rectangle. The *nad5* gene of *Lophophyphytum mirabile* is chimeric []. The tree depicts the best estimate of relationships among the plant species. Only intact genes are shown; pseudogenes were not included. **Fig. S15.** Evolution of *KARRIKIN INSENSITIVE2 *genes in *Sapria himalayana*. The phylogenetic tree indicates the relationship among the *KAI2 *genes in represent species. Conserved, intermediate, and divergentclades are shown in rose, indigo, and purple, respectively. Species names are abbreviated as follows: *Arabidopsis thaliana *, *Cuscuta australis *, *Cuscutacampestris *, *Coffea canephora *, *Cucumis sativus *, *Ipomoea nil *, *Jatropha curcas*, *Lindenbergia luchunensis *, *Linum usitatissimum *, *Mimulus guttatus *, *Manihotesculenta *, *Mimulus guttatus *, *Orobanche cumana *, *Phelipanche aegyptiaca *, *Papaver somniferum *, *Populus trichocarpa *, *Ricinus communis *, *Striga asiatica *, *S. himalayana *, *Sesamum indicum *, *Solanum lycopersicum *, and *Vitis vinifera *. Genes in *S. himalayana *are highlighted in red. **Figure S16.** Overview of gene expression. The transcriptome data of bract, sepal, petal, and reproductive diskproduced in our study, were used to quantify the expression level of gene in our study and Cai et al. [], respectively.The overview of gene expression in our study. Gene with expression and without expression was highlighted in red and blue, respectively.The overview of gene expression in Cai et al. []. Gene with expression and without expression was highlighted in red and green, respectively. **Fig. S17.** Intron length disparity. Intron length distribution of genesand HGTsin *Sapria himalayana* genome. 7256genes had at least one long intron. **Fig. S18.** Number of nuclear-encoded plastid-targeting genes in different categories from *Sapria himalayana *genome. **Fig. S19.** Principal component analysis among samples from unopened flower bud of *Sapria himalayana*.All samples for transcriptome.Removal of abnormal samples for transcriptome. **Fig. S20.** Sequencing depths of intact mitochondrial protein-coding genes in *Sapria himalayana*. *Sdh4_N *represents the vertical copy of *sdh4*, and *sdh4-F *represents the foreign copy.

**Additional file 2: Table S1.** Statistics and characteristics of the *Sapria himalayana *genome. **Table S2.** Validation of genome assembly using BUSCO. Table S3. The statistics and characteristics of RNA-seq and Iso-seq. **Table S4.** Statistics of predicted protein-coding genes in related species. **Table S5.** Functional annotation of *Sapria himalayana* protein-coding genes. **Table S6.** Statistics of different kinds of repeat types. **Table S7.** Annotation of conserved non-coding RNA genes in the *Sapria himalayana* genome. **Table S8.** Orthogroups comprising genes from *Arabidopsis thaliana*, *Cuscuta australis*, *Manihot esculenta*, *Populus trichocarpa*, *Sapria himalayana* and *Vitis vinifera*. Table S9. MapMan categories of lost genes in *Sapria himalayana*. **Table S10.** MapMan categories of duplicated genes in *Sapria himalayana*. **Table S11.** MapMan categories of species-specific genes in *Sapria himalayana*. **Table S12.** Presence and absence of genes involved in leaf and root development. **Table S13.** Presence and absence of genes involved in nutrient uptake and transport. **Table S14.** Genes and corresponding GeneBank accession numbers. **Table S15.** Number of genes related to cell proliferation and cell expansion in petals. **Table S16.** Presence and absence of genes involved in the regulation of flowering time. **Table S17.** The full name of genes related to fatty acid and amino acid. **Table S18.** HGT candidates in *Sapria himalayana*. **Table S19**. Overview of intron sequence in HGT sequences in *Sapria himalayana*. **Table S20.** Transcript levels of HGTs with- or without introns in flower buds of *Sapria himalayana*. **Table S21.** MapMan categories of horizontal gene transfersin the nuclear genome of *Sapria himalayana*. **Table S22.** Presence and absence of genes involved in salicylic acid biosynthesis. **Table S23.** Information of some HGT orthogroups including tree_id, HGT genes, corresponding native genes, intron, expression, selective constraint, and annotation. **Table S24.** PAML analyses with the branch test of HGT orthogroups, testing mode of selection in HGT sequencescompared to the background. **Table S25.** RELAX analyses of HGT orthogroups testing intensified selection or relaxed constraint in HGT sequences compared to the background. **Table S26.** The transcript levels of HGTs in the flower tissues of *Sapria himalayana*. Table S27. Positions, sizes and strand orientation of mitochondrial genes of *Sapria himalayana*. **Table S28.** Intron length of genes with the highest expression level in the top 1000 in *Sapria himalayana* flower buds. **Table S29.** Intron length of genes were responsible for floral development, hormones, flowering, haustorium, and fatty acid, sugar and amino acid biosynthesis and transport in *Sapria himalayana*. **Table S30.** Heatmap showing the log_2_-tranformed expression profile of HGT genes shared HGT functions among *Sapria himalayana*, *Cuscuta* and Orobanchaceae. **Table S31.** Summary of the selected genomes used in this study. **Table S32.** Orthogroup classification for the identification of HGTs among 14 representative species. **Table S33.** NCBI accession number of mitogenomes. **Table S34.** NCBI accession number of sequencing, assembly, and annotation data.

## Data Availability

All raw sequencing data used for de novo whole-genome assembly and annotations of *S. himalayana* have been deposited to NCBI under BioProject ID PRJNA797720 [130], and transcriptomic data were under BioProject ID PRJNA943542 [131]. And all of the accession numbers of Sequence Read Archive (SRA) were listed in Additional file 2: Table S34.

## Abbreviations

HGT: Horizontal gene transfer
FT: Flowering locus T
FA: Fatty acid
AAA: Aromatic amino acid
BUSCO: Benchmarking Universal Single Copy Orthologs
BWA: Burrows-Wheeler Aligner
TE: Transposable element
RNA-Seq: RNA-Sequencing
Iso-seq: Isoform sequencing
CDS: Coding sequence
ncRNA: Non-coding RNA
rRNA: Ribosomal RNA
miRNA: MicroRNA
tRNA: Transfer RNA
snRNA: Small nuclear RNA
TM6: Tomato MADS-box gene 6
FL: Fruitful-like
MP/ARF5: Monopteros/Auxin response factor 5
ANT: AINTEGUMENTA
GIF1: GRF-Interacting factor 1
SWP: STRUWWELPETER
MED25: Mediator complex subunit 25
BPEp: BIGPETALp
SAM: Shoot apical meristem
GA: Gibberellin
CO: CONSTANS
FLC: Flowering locus C
SOC1: Suppressor of overexpression of constans 1
ACCase: Acetyl-CoA carboxylase
GO: Gene ontology
FRK: FRUCTOKINASE
CSLE1: Cellulose synthase like E1
ATR4: Altered tryptophan regulation 4
RLK: Receptor-like kinase
RLP: Receptor-like protein
NBS–LRR: Nucleotide-binding site–leucine-rich repeat
AAO: Aldehyde oxidase
SA: Salicylic acid
FPKM: Fragments per kilobase per million mapped
SL: Strigolactone
KAR: Karrikin
KAI2: Karrikin insensitive 2
D14: DWARF 14
KL: KAI2 ligand
LRD: Lateral root development
LAX3: LIKE AUX 3
LBD18: LOB domain-containing protein 18
SHY2: Short hypocotyl 2
LFY: LEAFY
AP1: APETALA 1
AG: AGAMOUS
EVM: EVidenceModeler
SMRT: Single molecule real-time
PASA: Program to Assemble Spliced Alignments
Nr: NCBI non-redundant
CCS: Circular consensus sequence
FLNC: Full-length non-chimeric
nFL: Non-full-length
ICE: Isoform-level clustering
PCA: Principal component analysis
NCBI: National Center for Biotechnology Information
TAIR: The Arabidopsis Information Resource
dN: Non-synonymous substitution
dS: Synonymous substitution
Nt: Nucleotide
SRA: Sequence read archive
