# Supplementary material for: The Sapria himalayana genome provides new insights into the lifestyle of endoparasitic plants
Source: BMC Biol. 2023 Jun 6;21:134. doi: 10.1186/s12915-023-01620-3 (PMC10246380; doi:10.1186/s12915-023-01620-3)
Supplement: Supplementary file 1 — Additional file 1: Fig. S1. Evaluation of Sapria himalayana genome by k-mer analysis. X-axis shows the k-mer depth, and Y-axis depicts the k-mer frequency. The genome size of S. himalayana was estimated as 2,822.01 Mb. Fig. S2. Schematic diagram on stratified sampling from flower bud of Sapria himalayana. Tissues from the second, fourth, sixth, and eighth layers were used for RNA-seq and Iso-seq. The outermost host bark was removed prior to sampling. Fig. S3. Functional enrichment analysis of species-specific genes in Sapria himalayana. MapMan categories of significantly enriched genes are displayed using the logarithmic values of their P-values. Fig. S4. Phylogenetic tree of MADS-box gene family. Species names are abbreviated as follows: Arabidopsis thaliana, Manihot esculenta, Populus trichocarpa, Rafflesia cantleyi, and Sapria himalayana. Genes from S. himalayana and R. cantleyi are highlighted in red and brown colors, respectively. BS > 50% are shown. Fig. S5. Phylogenetic inference and sequence similarity of TOMATO MADS-BOX GENE6and FRUITFUL-like.Neighbor-joining tree of the newly isolated euAP3 and TM6homologs.Neighbor-joining tree of FRUITFUL-likehomologs in represent species. Information on gene sequences is provided in Table S14. Fig. S6. Heatmap showing the log2-tranformed expression profile of genes regulating flowering time. Host-derived mRNAs are highlighted in purple. Fig. S7. Phylogenetic tree of acetyl-CoA carboxylase . Species names are abbreviated as follows: Arabidopsis thaliana , Cucumis sativus , Eucalyptus grandis , Glycine max , Gossypium raimendii , Manihot esculenta , Mimulus guttatus , Oryza sativa , Papaver somniferum , Populus trichocarpa , Quercus robur , Sapria himalayana , and Vitis vinifera . Fig. S8. Horizontal gene transferevents in the nuclear genome of Sapria himalayana. Species names are abbreviated as follows: Arabidopsis thaliana, Cucumis sativus, Cuscuta australis , Eucalyptus grandis, Glycine max, Gossypium raimendii, Manihot [file 12915_2023_1620_MOESM1_ESM.pdf]

The *Sapria himalayana* genome provides new insights into the lifestyle of endoparasitic plants

Xuelian Guo[1,#], Xiaodi Hu[2,#], Jianwu Li[3], Bingyi Shao[1], Yajun Wang[1],Long Wang[2], Kui Li[2], Dongliang Lin[1], Hanchen Wang[1], Zhiyuan Gao[1], Yuannian Jiao[1], Yingying Wen[1], Hongyu Ji[1], Chongbo Ma[1], Song Ge[1], Wenkai Jiang[2, *], Xiaohua Jin[1,*]

#, these authors contributed equally.
*, corresponding author. Email: xiaohuajin@ibcas.ac.cn;
jiangwenkai@novogene.com.

This PDF file includes:

Supplementary Figures 1 to 20.

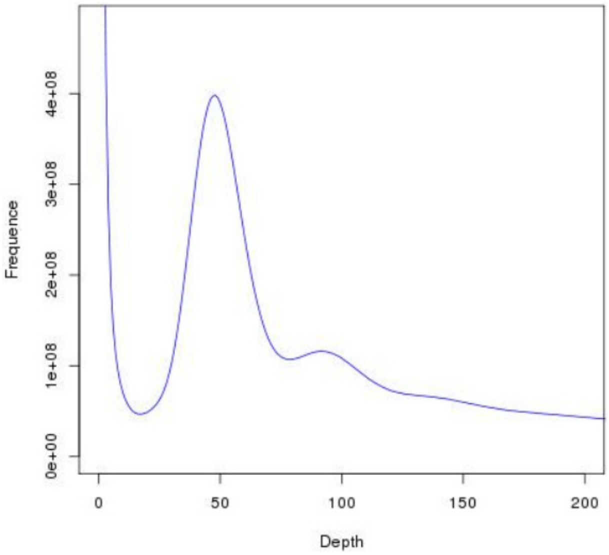

Figure S1. **Evaluation of *Sapria himalayana* genome by k-mer analysis**. X-axis shows the k-mer depth, and Y-axis depicts the k-mer frequency. The genome size of *S. himalayana* was estimated as 2,822.01 Mb.

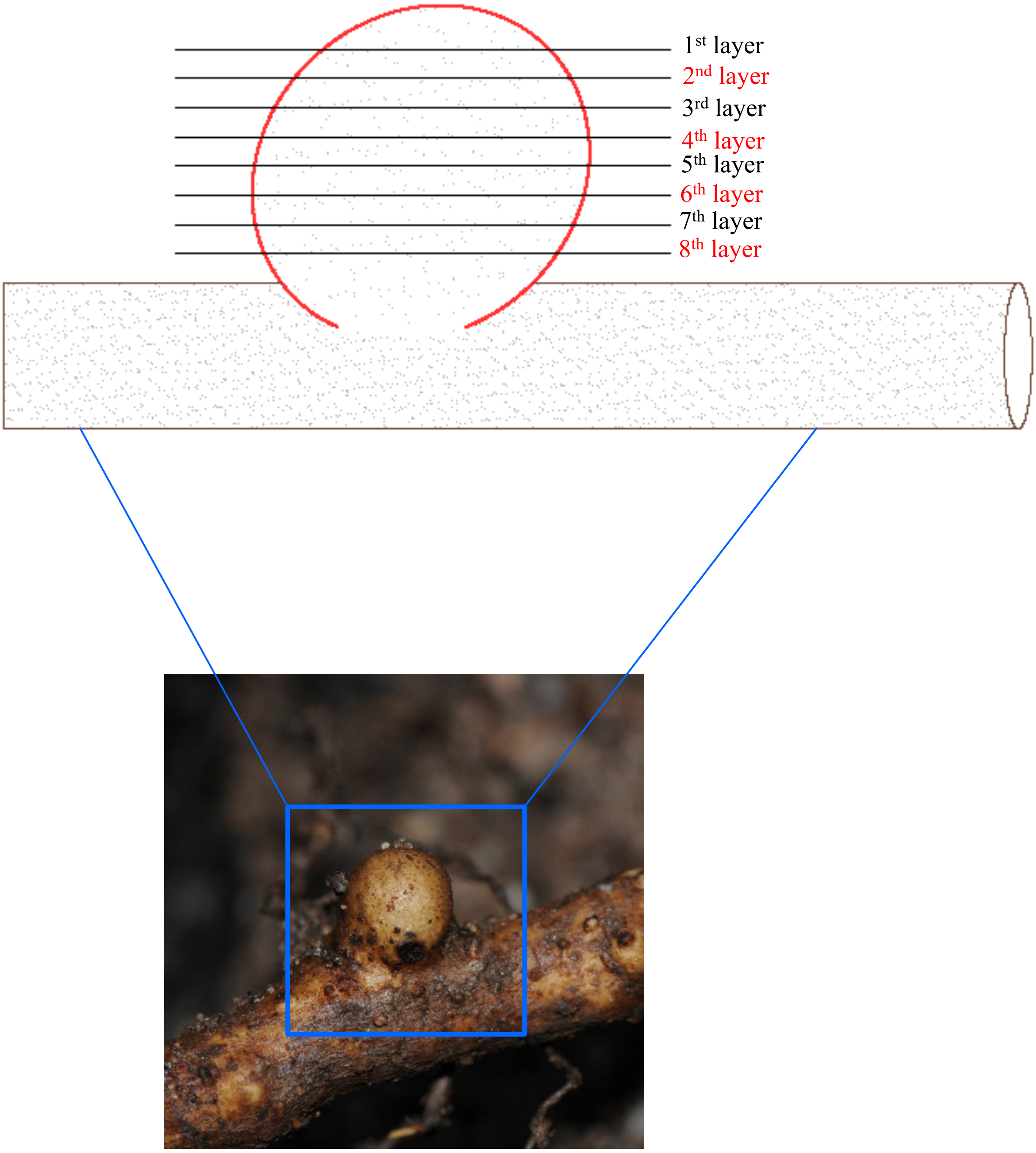

Figure S2. Schematic diagram on stratified sampling from flower bud of *Sapria himalayana*. Tissues from the second, fourth, sixth, and eighth layers were used for RNA-seq and Iso-seq. The outermost host bark was removed prior to sampling.

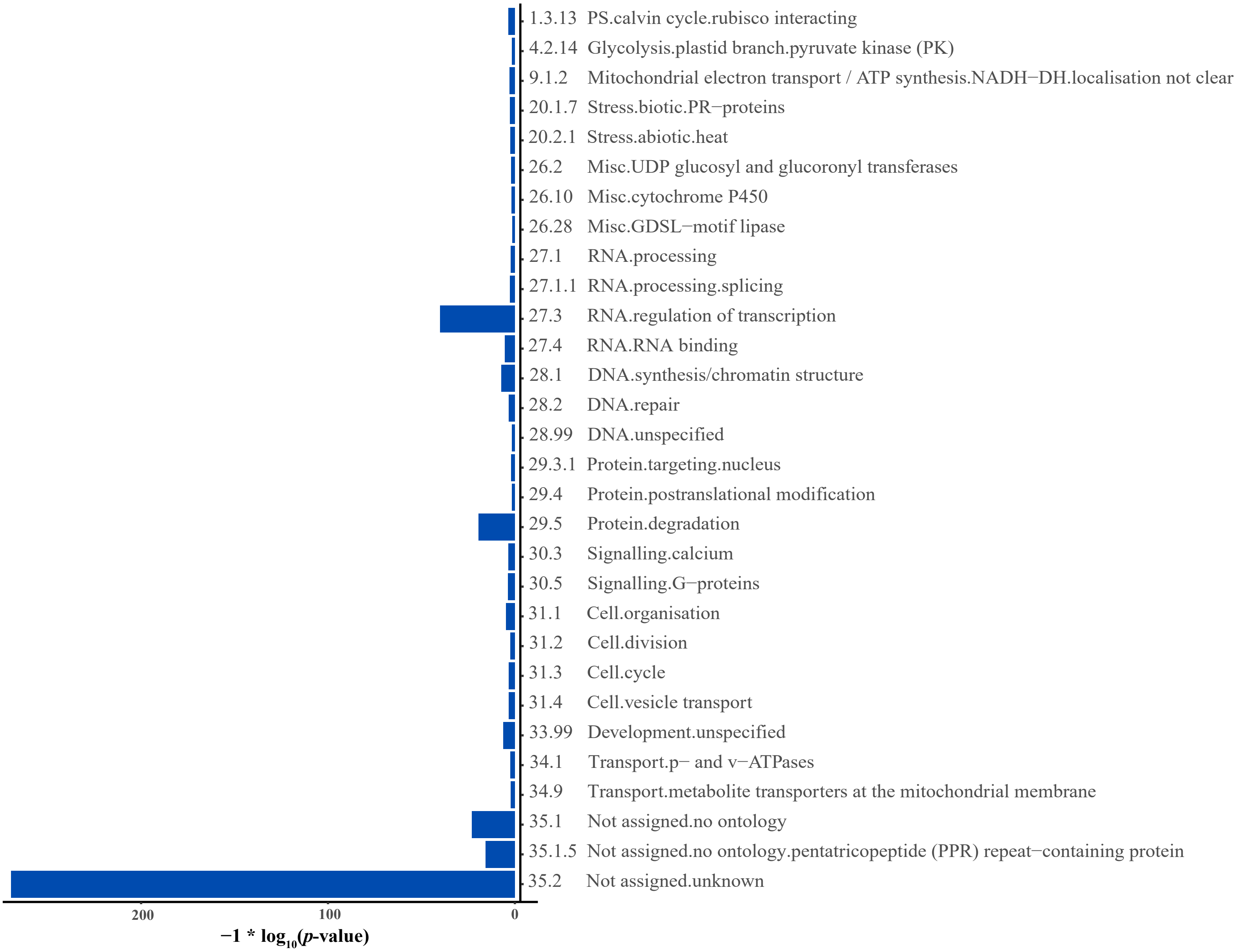

Figure S3. **Functional enrichment analysis of species-specific genes in *Sapria himalayana***. MapMan categories of significantly enriched genes are displayed using the logarithmic values of their *P*-values.

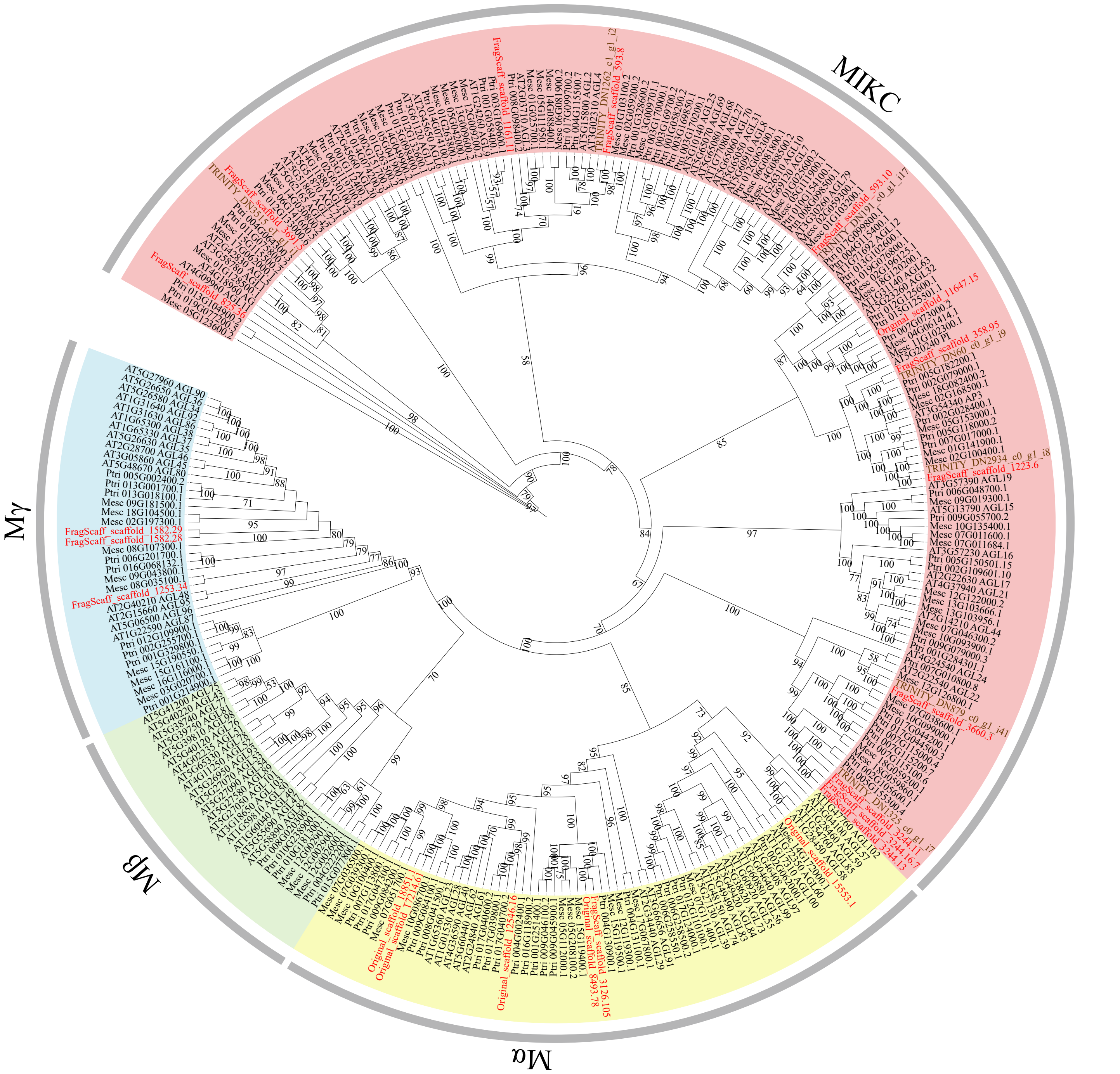

Figure S4. **Phylogenetic tree of MADS-box gene family**. Species names are abbreviated as follows: *Arabidopsis thaliana* (Atha), *Manihot esculenta* (Mesc), *Populus trichocarpa* (Ptri), *Rafflesia cantleyi* (Rcan), and *Sapria himalayana* (Shim). Genes from *S. himalayana* and *R. cantleyi* are highlighted in red and brown colors, respectively. BS > 50% are shown.

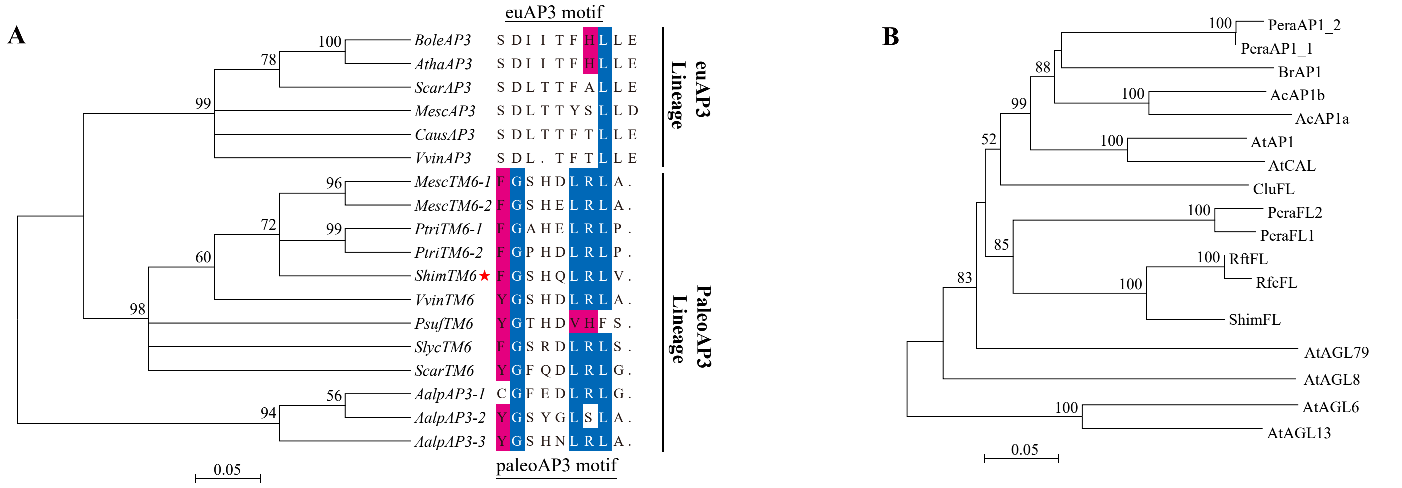

Figure S5. **Phylogenetic inference and sequence similarity of *TOMATO MADS-BOX GENE6* (*TM6*) and *FRUITFUL-like* (*FL*).** (A) Neighbor-joining tree of the newly isolated *euAP3* and *TM6* (*paleoAP3*) homologs. (B) Neighbor-joining tree of *FRUITFUL-like* (*FL*) homologs in represent species. Information on gene sequences is provided in Table S14.

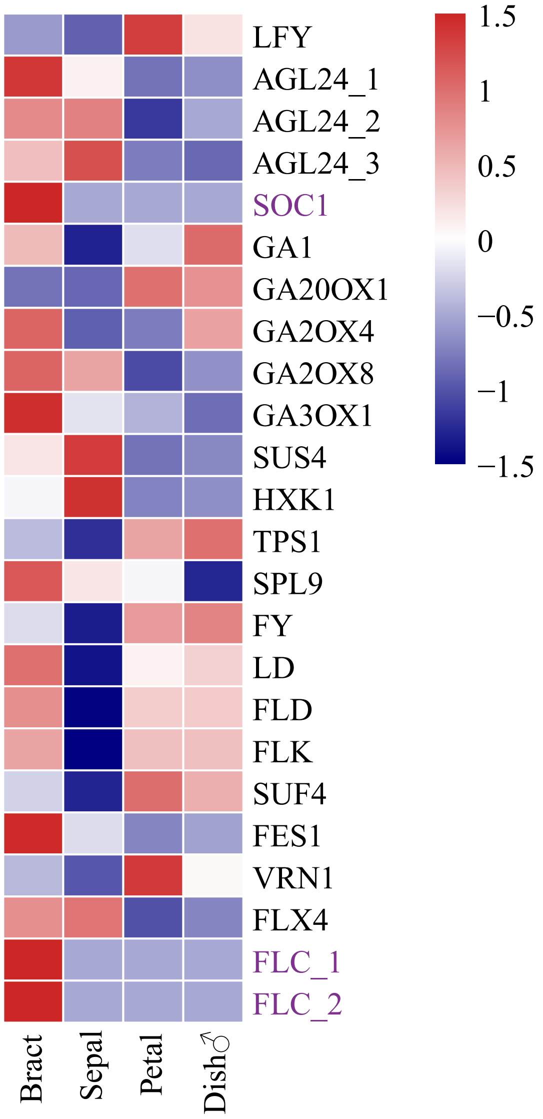

Figure S6. Heatmap showing the z-score tranformed expression profile of genes regulating flowering time. Host-derived mRNAs are highlighted in purple. Disk♂, disk with stamens.

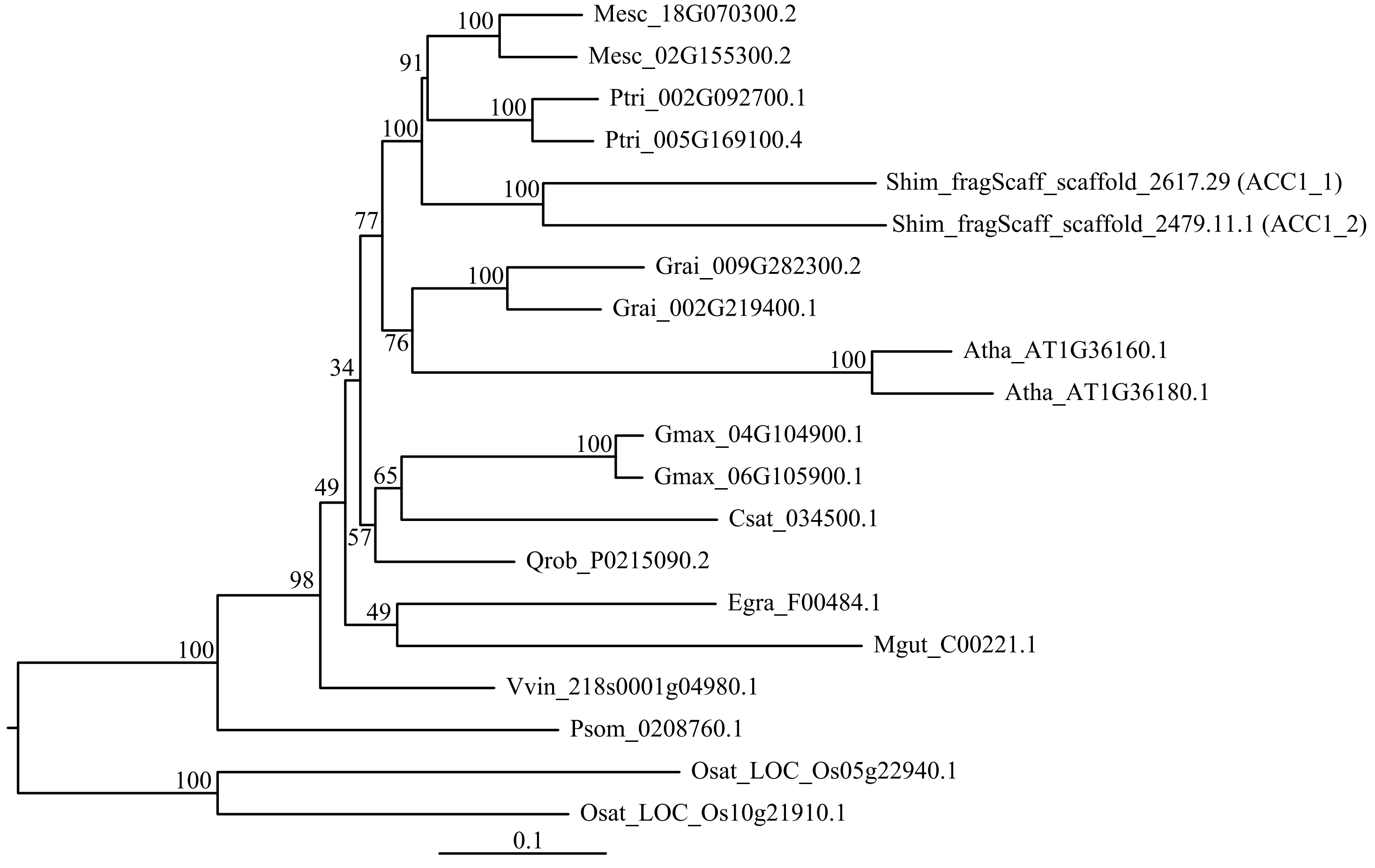

Figure S7. **Phylogenetic tree of *acetyl-CoA carboxylase* (*ACC*)**. Species names are abbreviated as follows: *Arabidopsis thaliana* (Atha), *Cucumis sativus* (Csat), *Eucalyptus grandis* (Egra), *Glycine max* (Gmax), *Gossypium raimendii* (Grai), *Manihot esculenta* (Mesc), *Mimulus guttatus* (Mgut), *Oryza sativa* (Osat), *Papaver somniferum* (Psom), *Populus trichocarpa* (Ptri), *Quercus robur* (Qrob), *Sapria himalayana* (Shim), and *Vitis vinifera* (Vvin).

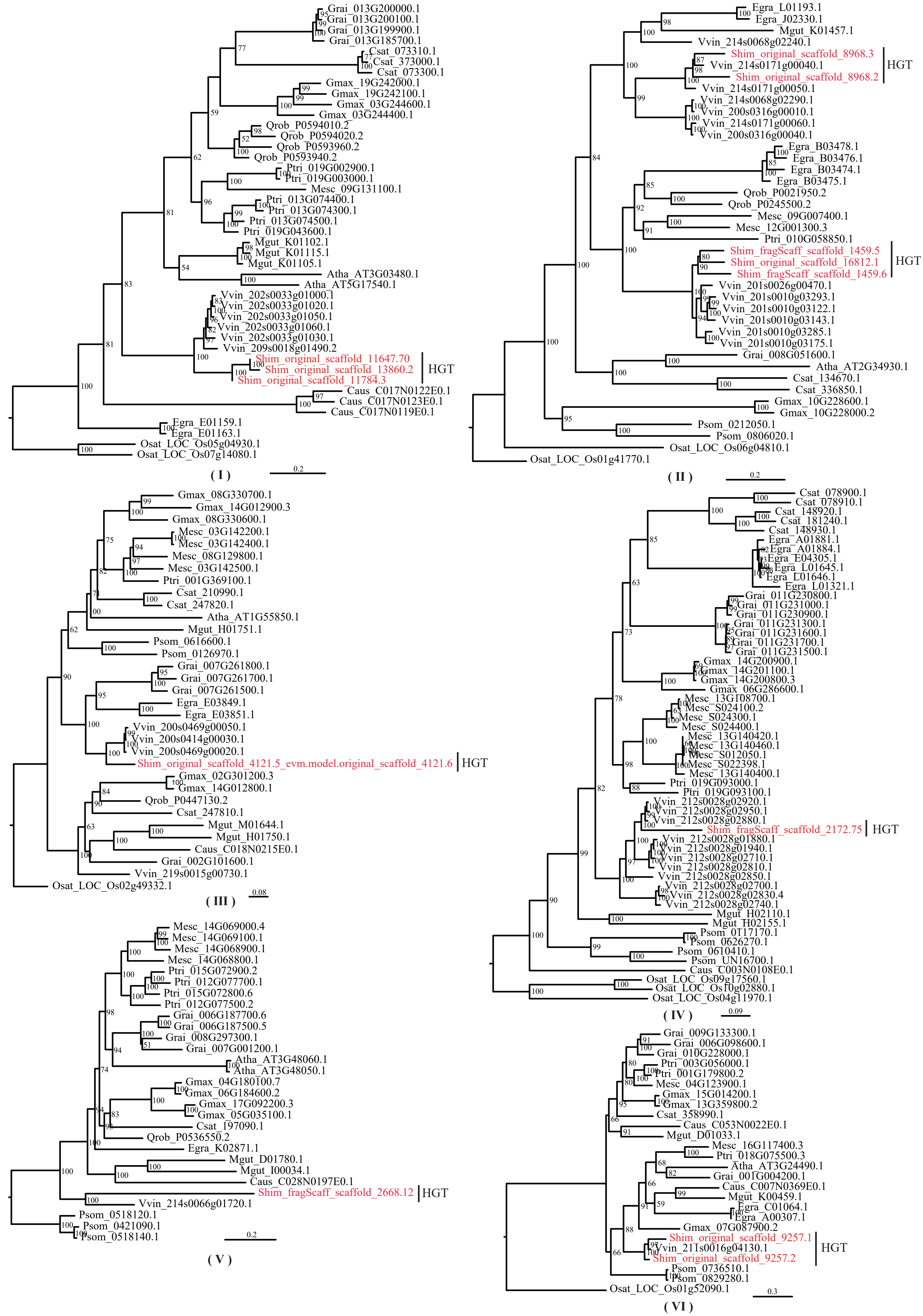
Grai_013G200000.1
Grai_013G200100.1
Grai_013G199900.1
Grai_013G185700.1
Csat_073310.1
Csat_373000.1
Csat_073300.1
Gmax_19G242000.1
Gmax_19G242100.1
Gmax_03G244600.1
Gmax_03G244400.1
Qrob_P0594010.2
Qrob_P0594020.2
Qrob_P0593960.2
Qrob_P0593940.2
Ptri_019G002900.1
Ptri_019G003000.1
Mesc_09G131100.1
Ptri_013G074400.1
Ptri_013G074300.1
Ptri_013G074500.1
Ptri_019G043600.1
Mgut_K01102.1
Mgut_K01115.1
Mgut_K01105.1
Atha_AT3G03480.1
Atha_AT5G17540.1
Vvin_202s0033g01000.1
Vvin_202s0033g01020.1
Vvin_202s0033g01050.1
Vvin_202s0033g01060.1
Vvin_202s0033g01030.1
Vvin_209s0018g01490.2
Shim_original_scaffold_11647.70
Shim_original_scaffold_13860.2
Shim_original_scaffold_11784.3
HGT
Caus_C017N0122E0.1
Caus_C017N0123E0.1
Caus_C017N0119E0.1
Egra_E01159.1
Egra_E01163.1
Osat_LOC_Os05g04930.1
Osat_LOC_Os07g14080.1
0.2
(I)
Egra_L01193.1
Egra_J02330.1
Mgut_K01457.1
Vvin_214s0068g02240.1
Shim_original_scaffold_8968.3
Vvin_214s0171g00040.1
Shim_original_scaffold_8968.2
HGT
Vvin_214s0171g00050.1
Vvin_214s0068g02290.1
Vvin_200s0316g00010.1
Vvin_214s0171g00060.1
Vvin_200s0316g00040.1
Egra_B03478.1
Egra_B03476.1
Egra_B03474.1
Egra_B03475.1
Qrob_P0021950.2
Qrob_P0245500.2
Mesc_09G007400.1
Mesc_12G001300.3
Ptri_010G058850.1
Shim_fragScaff_scaffold_1459.5
Shim_original_scaffold_16812.1
Shim_fragScaff_scaffold_1459.6
HGT
Vvin_201s0026g00470.1
Vvin_201s0010g03293.1
Vvin_201s0010g03122.1
Vvin_201s0010g03143.1
Vvin_201s0010g03285.1
Vvin_201s0010g03175.1
Grai_008G051600.1
Atha_AT2G34930.1
Csat_134670.1
Csat_336850.1
Gmax_10G228600.1
Gmax_10G228000.2
Psom_0212050.1
Psom_0806020.1
Osat_LOC_Os06g04810.1
Osat_LOC_Os01g41770.1
0.2
(II)
Gmax_08G330700.1
Gmax_14G012900.3
Gmax_08G330600.1
Mesc_03G142200.1
Mesc_03G142400.1
Mesc_08G129800.1
Mesc_03G142500.1
Ptri_001G369100.1
Csat_210990.1
Csat_247820.1
Atha_AT1G55850.1
Mgut_H01751.1
Psom_0616600.1
Psom_0126970.1
Grai_007G261800.1
Grai_007G261700.1
Grai_007G261500.1
Egra_E03849.1
Egra_E03851.1
Vvin_200s0469g00050.1
Vvin_200s0414g00030.1
Vvin_200s0469g00020.1
Shim_original_scaffold_4121.5_evm.model.original_scaffold_4121.6
HGT
Gmax_02G301200.3
Gmax_14G012800.1
Qrob_P0447130.2
Csat_247810.1
Mgut_M01644.1
Mgut_H01750.1
Caus_C018N0215E0.1
Grai_002G101600.1
Vvin_219s0015g00730.1
Osat_LOC_Os02g49332.1
0.08
(III)
Csat_078900.1
Csat_078910.1
Csat_148920.1
Csat_181240.1
Csat_148930.1
Egra_A01881.1
Egra_A01884.1
Egra_E04305.1
Egra_L01645.1
Egra_L01646.1
Egra_L01321.1
Grai_011G230800.1
Grai_011G231000.1
Grai_011G230900.1
Grai_011G231300.1
Grai_011G231600.1
Grai_011G231700.1
Grai_011G231500.1
Gmax_14G200900.1
Gmax_14G201100.1
Gmax_14G200800.3
Gmax_06G286600.1
Mesc_13G108700.1
Mesc_S024100.2
Mesc_S024300.1
Mesc_S024400.1
Mesc_13G140420.1
Mesc_13G140460.1
Mesc_S012050.1
Mesc_S022398.1
Mesc_13G140400.1
Ptri_019G093000.1
Ptri_019G093100.1
Vvin_212s0028g02920.1
Vvin_212s0028g02950.1
Vvin_212s0028g02880.1
Shim_fragScaff_scaffold_2172.75
HGT
Vvin_212s0028g01880.1
Vvin_212s0028g01940.1
Vvin_212s0028g02710.1
Vvin_212s0028g02810.1
Vvin_212s0028g02850.1
Vvin_212s0028g02700.1
Vvin_212s0028g02830.4
Vvin_212s0028g02740.1
Mgut_H02110.1
Mgut_H02155.1
Psom_0117170.1
Psom_0626270.1
Psom_0610410.1
Psom_UN16700.1
Caus_C003N0108E0.1
Osat_LOC_Os09g17560.1
Osat_LOC_Os10g02880.1
Osat_LOC_Os04g11970.1
(IV)
0.09
Mesc_14G069000.4
Mesc_14G069100.1
Mesc_14G068900.1
Mesc_14G068800.1
Ptri_015G072900.2
Ptri_012G077700.1
Ptri_015G072800.6
Ptri_012G077500.2
Grai_006G187700.6
Grai_006G187500.5
Grai_008G297300.1
Grai_007G001200.1
Atha_AT3G48060.1
Atha_AT3G48050.1
Gmax_04G180100.7
Gmax_06G184600.2
Gmax_17G092200.3
Gmax_05G035100.1
Csat_197090.1
Qrob_P0536550.2
Egra_K02871.1
Mgut_D01780.1
Mgut_I00034.1
Caus_C028N0197E0.1
Shim_fragScaff_scaffold_2668.12
HGT
Vvin_214s0066g01720.1
Psom_0518120.1
Psom_0421090.1
Psom_0518140.1
0.2
(V)
Grai_009G133300.1
Grai_006G098600.1
Grai_010G228000.1
Ptri_003G056000.1
Ptri_001G179800.2
Mesc_04G123900.1
Gmax_15G014200.1
Gmax_13G359800.2
Csat_358990.1
Caus_C053N0022E0.1
Mgut_D01033.1
Mesc_16G117400.3
Ptri_018G075500.3
Atha_AT3G24490.1
Grai_001G004200.1
Caus_C007N0369E0.1
Mgut_K00459.1
Egra_C01064.1
Egra_A00307.1
Gmax_07G087900.2
Shim_original_scaffold_9257.1
Vvin_211s0016g04130.1
Shim_original_scaffold_9257.2
HGT
Psom_0736510.1
Psom_0829280.1
Osat_LOC_Os01g52090.1
0.3
(VI)

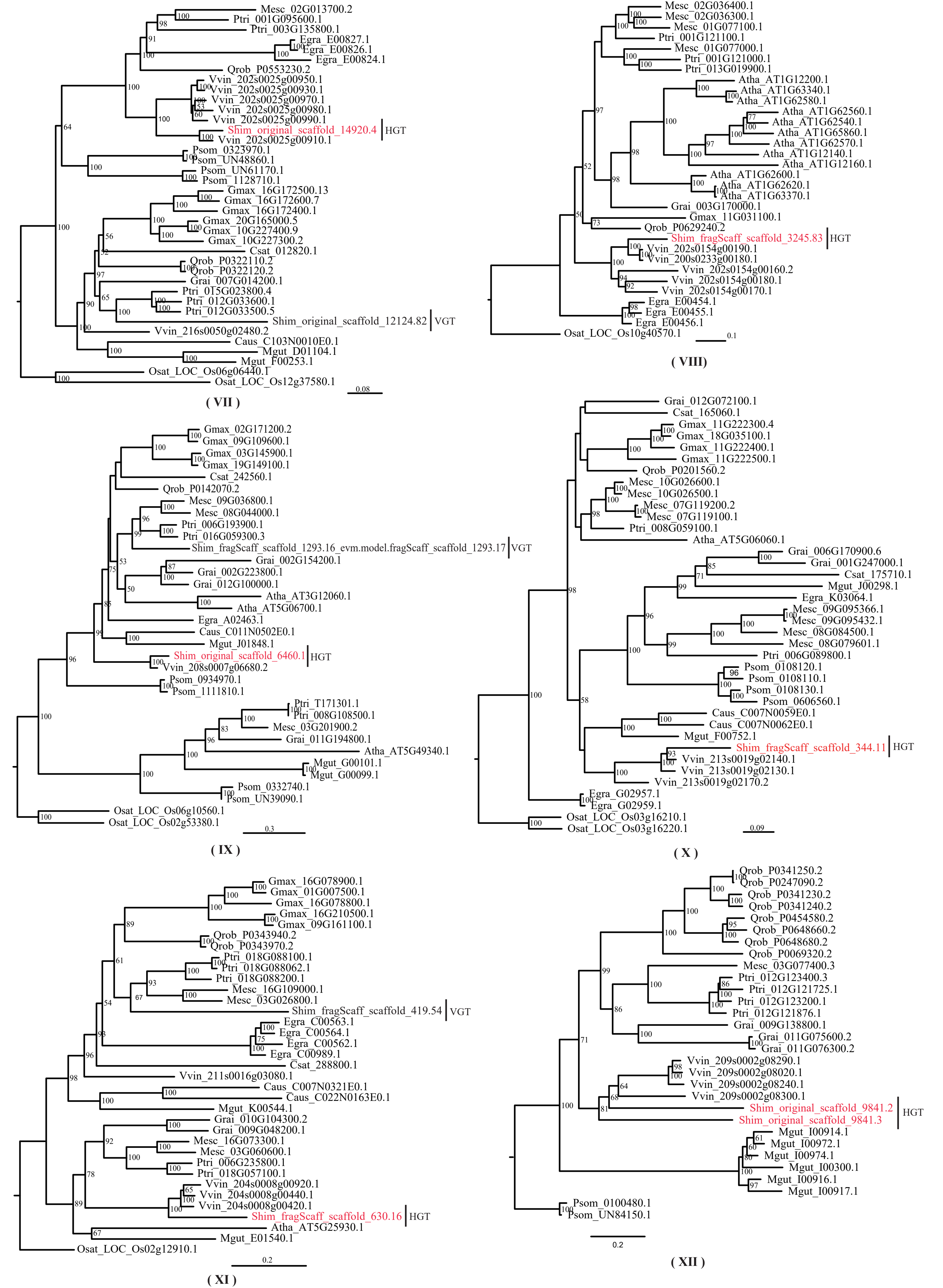
Mesc_02G013700.2
Ptri_001G095600.1
Ptri_003G135800.1
Egra_E00827.1
Egra_E00826.1
Egra_E00824.1
Qrob_P0553230.2
Vvin_202s0025g00950.1
Vvin_202s0025g00930.1
Vvin_202s0025g00970.1
Vvin_202s0025g00980.1
Vvin_202s0025g00990.1
Shim_original_scaffold_14920.4
HGT
Vvin_202s0025g00910.1
Psom_0323970.1
Psom_UN48860.1
Psom_UN61170.1
Psom_1128710.1
Gmax_16G172500.13
Gmax_16G172600.7
Gmax_16G172400.1
Gmax_20G165000.5
Gmax_10G227400.9
Gmax_10G227300.2
Csat_012820.1
Qrob_P0322110.2
Qrob_P0322120.2
Grai_007G014200.1
Ptri_015G023800.4
Ptri_012G033600.1
Ptri_012G033500.5
Shim_original_scaffold_12124.82
VGT
Vvin_216s0050g02480.2
Caus_C103N0010E0.1
Mgut_D01104.1
Mgut_F00253.1
Osat_LOC_Os06g06440.1
Osat_LOC_Os12g37580.1
0.08
(VII)
Mesc_02G036400.1
Mesc_02G036300.1
Mesc_01G077100.1
Ptri_001G121100.1
Mesc_01G077000.1
Ptri_001G121000.1
Ptri_013G019900.1
Atha_AT1G12200.1
Atha_AT1G63340.1
Atha_AT1G62580.1
Atha_AT1G62560.1
Atha_AT1G62540.1
Atha_AT1G65860.1
Atha_AT1G62570.1
Atha_AT1G12140.1
Atha_AT1G12160.1
Atha_AT1G62600.1
Atha_AT1G62620.1
Atha_AT1G63370.1
Grai_003G170000.1
Gmax_11G031100.1
Qrob_P0629240.2
Shim_fragScaff_scaffold_3245.83
HGT
Vvin_202s0154g00190.1
Vvin_200s0233g00180.1
Vvin_202s0154g00160.2
Vvin_202s0154g00180.1
Vvin_202s0154g00170.1
Egra_E00454.1
Egra_E00455.1
Egra_E00456.1
Osat_LOC_Os10g40570.1
0.1
(VIII)
Gmax_02G171200.2
Gmax_09G109600.1
Gmax_03G145900.1
Gmax_19G149100.1
Csat_242560.1
Qrob_P0142070.2
Mesc_09G036800.1
Mesc_08G044000.1
Ptri_006G193900.1
Ptri_016G059300.3
Shim_fragScaff_scaffold_1293.16_evm.model.fragScaff_scaffold_1293.17
VGT
Grai_002G154200.1
Grai_002G223800.1
Grai_012G100000.1
Atha_AT3G12060.1
Atha_AT5G06700.1
Egra_A02463.1
Caus_C011N0502E0.1
Mgut_J01848.1
Shim_original_scaffold_6460.1
HGT
Vvin_208s0007g06680.2
Psom_0934970.1
Psom_1111810.1
Ptri_T171301.1
Ptri_008G108500.1
Mesc_03G201900.2
Grai_011G194800.1
Atha_AT5G49340.1
Mgut_G00101.1
Mgut_G00099.1
Psom_0332740.1
Psom_UN39090.1
Osat_LOC_Os06g10560.1
Osat_LOC_Os02g53380.1
0.3
(IX)
Grai_012G072100.1
Csat_165060.1
Gmax_11G222300.4
Gmax_18G035100.1
Gmax_11G222400.1
Gmax_11G222500.1
Qrob_P0201560.2
Mesc_10G026600.1
Mesc_10G026500.1
Mesc_07G119200.2
Mesc_07G119100.1
Ptri_008G059100.1
Atha_AT5G06060.1
Grai_006G170900.6
Grai_001G247000.1
Csat_175710.1
Mgut_J00298.1
Egra_K03064.1
Mesc_09G095366.1
Mesc_09G095432.1
Mesc_08G084500.1
Mesc_08G079601.1
Ptri_006G089800.1
Psom_0108120.1
Psom_0108110.1
Psom_0108130.1
Psom_0606560.1
Caus_C007N0059E0.1
Caus_C007N0062E0.1
Mgut_F00752.1
Shim_fragScaff_scaffold_344.11
HGT
Vvin_213s0019g02140.1
Vvin_213s0019g02130.1
Vvin_213s0019g02170.2
Egra_G02957.1
Egra_G02959.1
Osat_LOC_Os03g16210.1
Osat_LOC_Os03g16220.1
0.09
(X)
Gmax_16G078900.1
Gmax_01G007500.1
Gmax_16G078800.1
Gmax_16G210500.1
Gmax_09G161100.1
Qrob_P0343940.2
Qrob_P0343970.2
Ptri_018G088100.1
Ptri_018G088062.1
Ptri_018G088200.1
Mesc_16G109000.1
Mesc_03G026800.1
Shim_fragScaff_scaffold_419.54
VGT
Egra_C00563.1
Egra_C00564.1
Egra_C00562.1
Egra_C00989.1
Csat_288800.1
Vvin_211s0016g03080.1
Caus_C007N0321E0.1
Caus_C022N0163E0.1
Mgut_K00544.1
Grai_010G104300.2
Grai_009G048200.1
Mesc_16G073300.1
Mesc_03G060600.1
Ptri_006G235800.1
Ptri_018G057100.1
Vvin_204s0008g00920.1
Vvin_204s0008g00440.1
Vvin_204s0008g00420.1
Shim_fragScaff_scaffold_630.16
HGT
Atha_AT5G25930.1
Mgut_E01540.1
Osat_LOC_Os02g12910.1
0.2
(XI)
Qrob_P0341250.2
Qrob_P0247090.2
Qrob_P0341230.2
Qrob_P0341240.2
Qrob_P0454580.2
Qrob_P0648660.2
Qrob_P0648680.2
Qrob_P0069320.2
Mesc_03G077400.3
Ptri_012G123400.3
Ptri_012G121725.1
Ptri_012G123200.1
Ptri_012G121876.1
Grai_009G138800.1
Grai_011G075600.2
Grai_011G076300.2
Vvin_209s0002g08290.1
Vvin_209s0002g08020.1
Vvin_209s0002g08240.1
Vvin_209s0002g08300.1
Shim_original_scaffold_9841.2
Shim_original_scaffold_9841.3
HGT
Mgut_I00914.1
Mgut_I00972.1
Mgut_I00974.1
Mgut_I00300.1
Mgut_I00916.1
Mgut_I00917.1
Psom_0100480.1
Psom_UN84150.1
0.2
(XII)

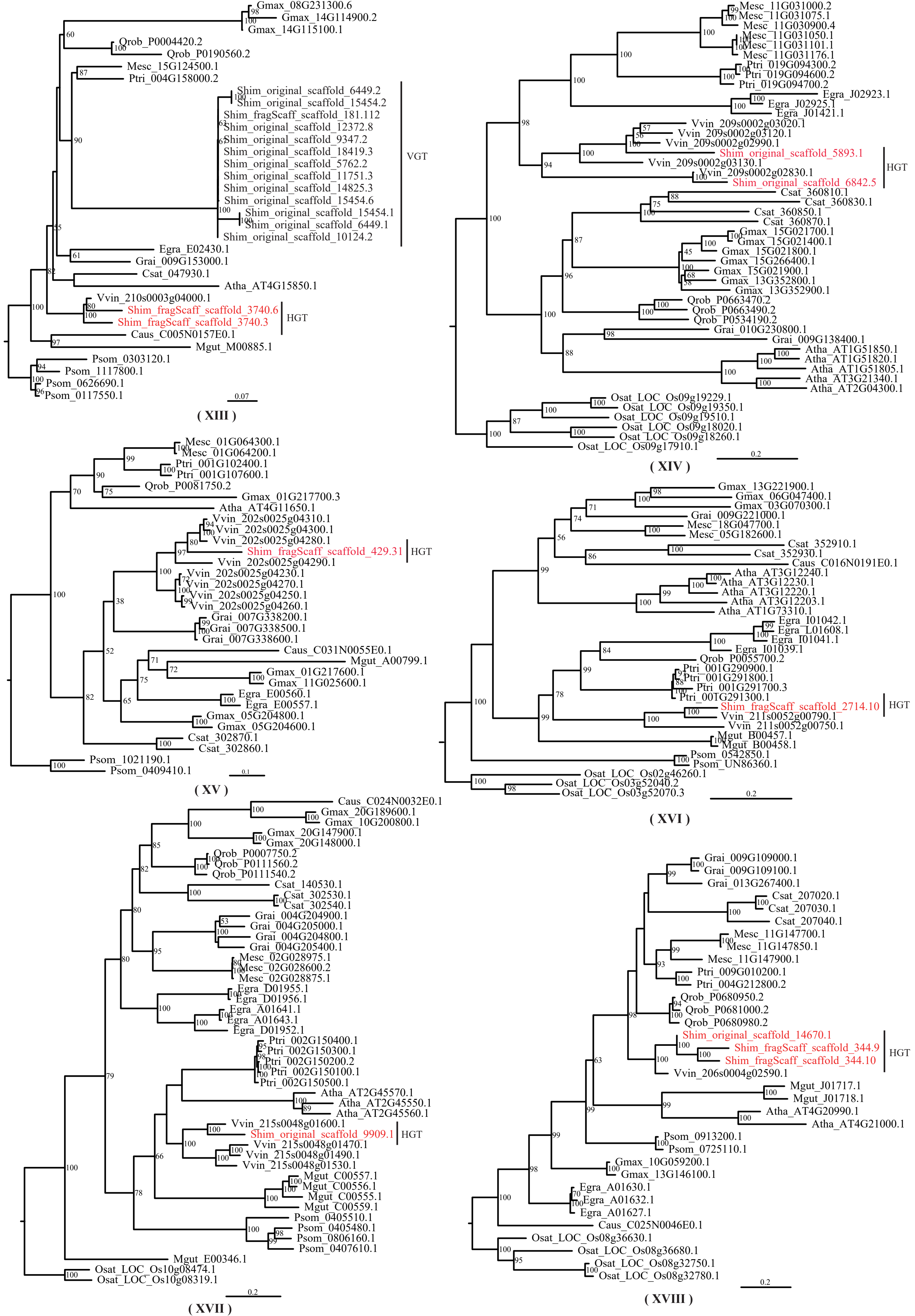
Gmax_08G231300.6
Gmax_14G114900.2
Gmax_14G115100.1
Qrob_P0004420.2
Qrob_P0190560.2
Mesc_15G124500.1
Ptri_004G158000.2
Shim_original_scaffold_6449.2
Shim_original_scaffold_15454.2
Shim_fragScaff_scaffold_181.112
Shim_original_scaffold_12372.8
Shim_original_scaffold_9347.2
Shim_original_scaffold_18419.3
Shim_original_scaffold_5762.2
Shim_original_scaffold_11751.3
Shim_original_scaffold_14825.3
Shim_original_scaffold_15454.6
Shim_original_scaffold_15454.1
Shim_original_scaffold_6449.1
Shim_original_scaffold_10124.2
VGT
Egra_E02430.1
Grai_009G153000.1
Csat_047930.1
Atha_AT4G15850.1
Vvin_210s0003g04000.1
Shim_fragScaff_scaffold_3740.6
Shim_fragScaff_scaffold_3740.3
HGT
Caus_C005N0157E0.1
Mgut_M00885.1
Psom_0303120.1
Psom_1117800.1
Psom_0626690.1
Psom_0117550.1
0.07
(XIII)
Mesc_11G031000.2
Mesc_11G031075.1
Mesc_11G030900.4
Mesc_11G031050.1
Mesc_11G031101.1
Mesc_11G031176.1
Ptri_019G094300.2
Ptri_019G094600.2
Ptri_019G094700.2
Egra_J02923.1
Egra_J02925.1
Egra_J01421.1
Vvin_209s0002g03020.1
Vvin_209s0002g03120.1
Vvin_209s0002g02990.1
Shim_original_scaffold_5893.1
Vvin_209s0002g03130.1
Vvin_209s0002g02830.1
Shim_original_scaffold_6842.5
HGT
Csat_360810.1
Csat_360830.1
Csat_360850.1
Csat_360870.1
Gmax_15G021700.1
Gmax_15G021400.1
Gmax_15G021800.1
Gmax_15G266400.1
Gmax_15G021900.1
Gmax_13G352800.1
Gmax_13G352900.1
Qrob_P0663470.2
Qrob_P0663490.2
Qrob_P0534190.2
Grai_010G230800.1
Grai_009G138400.1
Atha_AT1G51850.1
Atha_AT1G51820.1
Atha_AT1G51805.1
Atha_AT3G21340.1
Atha_AT2G04300.1
Osat_LOC_Os09g19229.1
Osat_LOC_Os09g19350.1
Osat_LOC_Os09g19510.1
Osat_LOC_Os09g18020.1
Osat_LOC_Os09g18260.1
Osat_LOC_Os09g17910.1
0.2
(XIV)
Mesc_01G064300.1
Mesc_01G064200.1
Ptri_001G102400.1
Ptri_001G107600.1
Qrob_P0081750.2
Gmax_01G217700.3
Atha_AT4G11650.1
Vvin_202s0025g04310.1
Vvin_202s0025g04300.1
Vvin_202s0025g04280.1
Shim_fragScaff_scaffold_429.31
HGT
Vvin_202s0025g04290.1
Vvin_202s0025g04230.1
Vvin_202s0025g04270.1
Vvin_202s0025g04250.1
Vvin_202s0025g04260.1
Grai_007G338200.1
Grai_007G338500.1
Grai_007G338600.1
Caus_C031N0055E0.1
Mgut_A00799.1
Gmax_01G217600.1
Gmax_11G025600.1
Egra_E00560.1
Egra_E00557.1
Gmax_05G204800.1
Gmax_05G204600.1
Csat_302870.1
Csat_302860.1
Psom_1021190.1
Psom_0409410.1
0.1
(XV)
Gmax_13G221900.1
Gmax_06G047400.1
Gmax_03G070300.1
Grai_009G221000.1
Mesc_18G047700.1
Mesc_05G182600.1
Csat_352910.1
Csat_352930.1
Caus_C016N0191E0.1
Atha_AT3G12240.1
Atha_AT3G12230.1
Atha_AT3G12220.1
Atha_AT3G12203.1
Atha_AT1G73310.1
Egra_I01042.1
Egra_L01608.1
Egra_I01041.1
Egra_I01039.1
Qrob_P0055700.2
Ptri_001G290900.1
Ptri_001G291800.1
Ptri_001G291700.3
Ptri_001G291300.1
Shim_fragScaff_scaffold_2714.10
HGT
Vvin_211s0052g00790.1
Vvin_211s0052g00750.1
Mgut_B00457.1
Mgut_B00458.1
Psom_0542850.1
Psom_UN86360.1
Osat_LOC_Os02g46260.1
Osat_LOC_Os03g52040.2
Osat_LOC_Os03g52070.3
0.2
(XVI)
Caus_C024N0032E0.1
Gmax_20G189600.1
Gmax_10G200800.1
Gmax_20G147900.1
Gmax_20G148000.1
Qrob_P0007750.2
Qrob_P0111560.2
Qrob_P0111540.2
Csat_140530.1
Csat_302530.1
Csat_302540.1
Grai_004G204900.1
Grai_004G205000.1
Grai_004G204800.1
Grai_004G205400.1
Mesc_02G028975.1
Mesc_02G028600.2
Mesc_02G028875.1
Egra_D01955.1
Egra_D01956.1
Egra_A01641.1
Egra_A01643.1
Egra_D01952.1
Ptri_002G150400.1
Ptri_002G150300.1
Ptri_002G150200.2
Ptri_002G150100.1
Ptri_002G150500.1
Atha_AT2G45570.1
Atha_AT2G45550.1
Atha_AT2G45560.1
Vvin_215s0048g01600.1
Shim_original_scaffold_9909.1
HGT
Vvin_215s0048g01470.1
Vvin_215s0048g01490.1
Vvin_215s0048g01530.1
Mgut_C00557.1
Mgut_C00556.1
Mgut_C00555.1
Mgut_C00559.1
Psom_0405510.1
Psom_0405480.1
Psom_0806160.1
Psom_0407610.1
Mgut_E00346.1
Osat_LOC_Os10g08474.1
Osat_LOC_Os10g08319.1
0.2
(XVII)
Grai_009G109000.1
Grai_009G109100.1
Grai_013G267400.1
Csat_207020.1
Csat_207030.1
Csat_207040.1
Mesc_11G147700.1
Mesc_11G147850.1
Mesc_11G147900.1
Ptri_009G010200.1
Ptri_004G212800.2
Qrob_P0680950.2
Qrob_P0681000.2
Qrob_P0680980.2
Shim_original_scaffold_14670.1
Shim_fragScaff_scaffold_344.9
Shim_fragScaff_scaffold_344.10
HGT
Vvin_206s0004g02590.1
Mgut_J01717.1
Mgut_J01718.1
Atha_AT4G20990.1
Atha_AT4G21000.1
Psom_0913200.1
Psom_0725110.1
Gmax_10G059200.1
Gmax_13G146100.1
Egra_A01630.1
Egra_A01632.1
Egra_A01627.1
Caus_C025N0046E0.1
Osat_LOC_Os08g36630.1
Osat_LOC_Os08g36680.1
Osat_LOC_Os08g32750.1
Osat_LOC_Os08g32780.1
0.2
(XVIII)

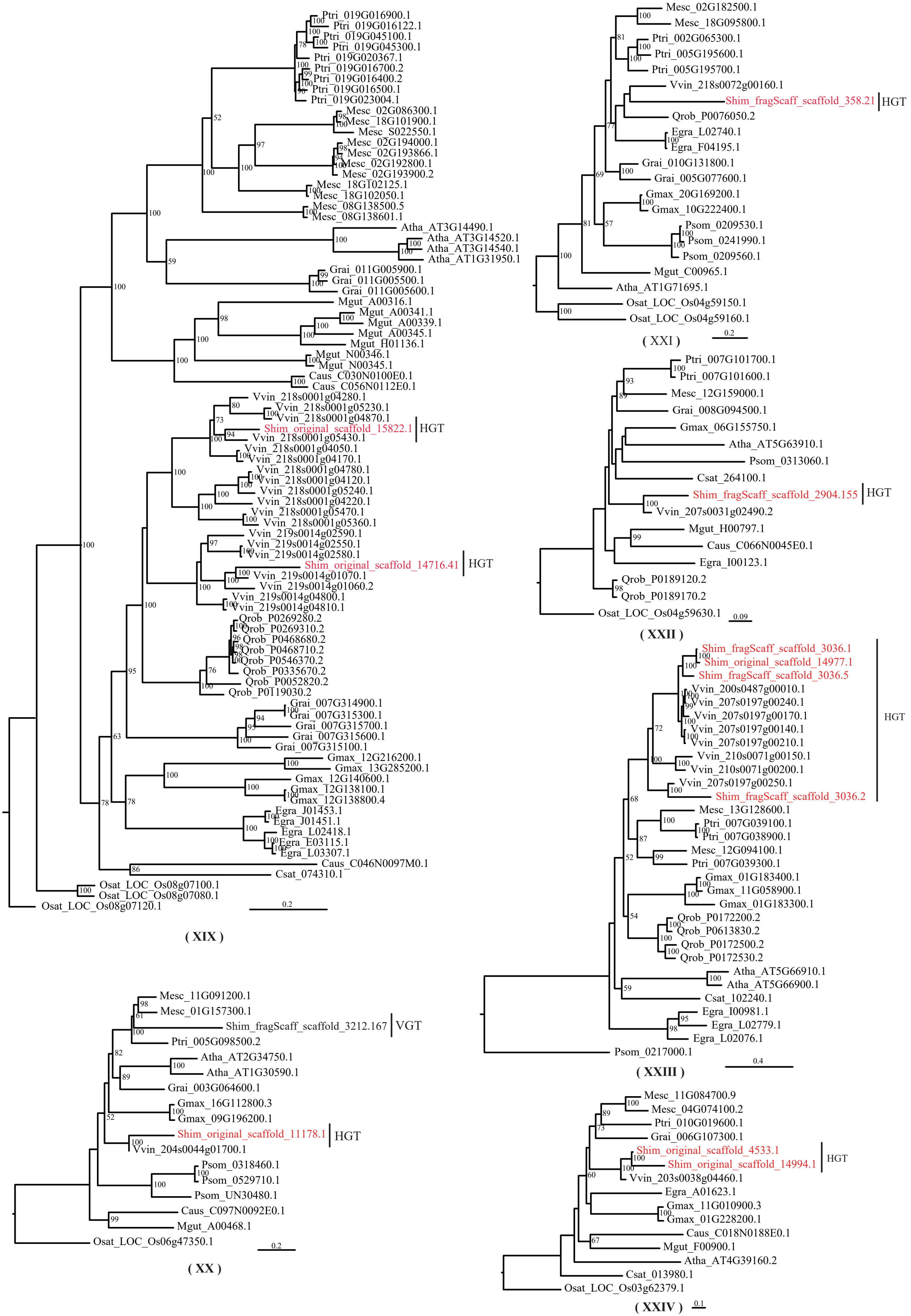

Ptri_019G016900.1
Ptri_019G016122.1
Ptri_019G045100.1
Ptri_019G045300.1
Ptri_019G020367.1
Ptri_019G016700.2
Ptri_019G016400.2
Ptri_019G016500.1
Ptri_019G023004.1
Mesc_02G086300.1
Mesc_18G101900.1
Mesc_S022550.1
Mesc_02G194000.1
Mesc_02G193866.1
Mesc_02G192800.1
Mesc_02G193900.2
Mesc_18G102125.1
Mesc_18G102050.1
Mesc_08G138500.5
Mesc_08G138601.1
Atha_AT3G14490.1
Atha_AT3G14520.1
Atha_AT3G14540.1
Atha_AT1G31950.1
Grai_011G005900.1
Grai_011G005500.1
Grai_011G005600.1
Mgut_A00316.1
Mgut_A00341.1
Mgut_A00339.1
Mgut_A00345.1
Mgut_H01136.1
Mgut_N00346.1
Mgut_N00345.1
Caus_C030N0100E0.1
Caus_C056N0112E0.1
Vvin_218s0001g04280.1
Vvin_218s0001g05230.1
Vvin_218s0001g04870.1
Shim_original_scaffold_15822.1
HGT
Vvin_218s0001g05430.1
Vvin_218s0001g04050.1
Vvin_218s0001g04170.1
Vvin_218s0001g04780.1
Vvin_218s0001g04120.1
Vvin_218s0001g05240.1
Vvin_218s0001g04220.1
Vvin_218s0001g05470.1
Vvin_218s0001g05360.1
Vvin_219s0014g02590.1
Vvin_219s0014g02550.1
Vvin_219s0014g02580.1
Shim_original_scaffold_14716.41
HGT
Vvin_219s0014g01070.1
Vvin_219s0014g01060.2
Vvin_219s0014g04800.1
Vvin_219s0014g04810.1
Qrob_P0269280.2
Qrob_P0269310.2
Qrob_P0468680.2
Qrob_P0468710.2
Qrob_P0546370.2
Qrob_P0335670.2
Qrob_P0052820.2
Qrob_P0119030.2
Grai_007G314900.1
Grai_007G315300.1
Grai_007G315700.1
Grai_007G315600.1
Grai_007G315100.1
Gmax_12G216200.1
Gmax_13G285200.1
Gmax_12G140600.1
Gmax_12G138100.1
Gmax_12G138800.4
Egra_J01453.1
Egra_J01451.1
Egra_L02418.1
Egra_E03115.1
Egra_L03307.1
Caus_C046N0097M0.1
Csat_074310.1
Osat_LOC_Os08g07100.1
Osat_LOC_Os08g07080.1
Osat_LOC_Os08g07120.1
0.2
(XIX)
Mesc_11G091200.1
Mesc_01G157300.1
Shim_fragScaff_scaffold_3212.167
VGT
Ptri_005G098500.2
Atha_AT2G34750.1
Atha_AT1G30590.1
Grai_003G064600.1
Gmax_16G112800.3
Gmax_09G196200.1
Shim_original_scaffold_11178.1
HGT
Vvin_204s0044g01700.1
Psom_0318460.1
Psom_0529710.1
Psom_UN30480.1
Caus_C097N0092E0.1
Mgut_A00468.1
Osat_LOC_Os06g47350.1
0.2
(XX)
Mesc_02G182500.1
Mesc_18G095800.1
Ptri_002G065300.1
Ptri_005G195600.1
Ptri_005G195700.1
Vvin_218s0072g00160.1
Shim_fragScaff_scaffold_358.21
HGT
Qrob_P0076050.2
Egra_L02740.1
Egra_F04195.1
Grai_010G131800.1
Grai_005G077600.1
Gmax_20G169200.1
Gmax_10G222400.1
Psom_0209530.1
Psom_0241990.1
Psom_0209560.1
Mgut_C00965.1
Atha_AT1G71695.1
Osat_LOC_Os04g59150.1
Osat_LOC_Os04g59160.1
0.2
(XXI)
Ptri_007G101700.1
Ptri_007G101600.1
Mesc_12G159000.1
Grai_008G094500.1
Gmax_06G155750.1
Atha_AT5G63910.1
Psom_0313060.1
Csat_264100.1
Shim_fragScaff_scaffold_2904.155
HGT
Vvin_207s0031g02490.2
Mgut_H00797.1
Caus_C066N0045E0.1
Egra_I00123.1
Qrob_P0189120.2
Qrob_P0189170.2
Osat_LOC_Os04g59630.1
0.09
(XXII)
Shim_fragScaff_scaffold_3036.1
Shim_original_scaffold_14977.1
Shim_fragScaff_scaffold_3036.5
Vvin_200s0487g00010.1
Vvin_207s0197g00240.1
Vvin_207s0197g00170.1
Vvin_207s0197g00140.1
Vvin_207s0197g00210.1
Vvin_210s0071g00150.1
Vvin_210s0071g00200.1
Vvin_207s0197g00250.1
Shim_fragScaff_scaffold_3036.2
HGT
Mesc_13G128600.1
Ptri_007G039100.1
Ptri_007G038900.1
Mesc_12G094100.1
Ptri_007G039300.1
Gmax_01G183400.1
Gmax_11G058900.1
Gmax_01G183300.1
Qrob_P0172200.2
Qrob_P0613830.2
Qrob_P0172500.2
Qrob_P0172530.2
Atha_AT5G66910.1
Atha_AT5G66900.1
Csat_102240.1
Egra_I00981.1
Egra_L02779.1
Egra_L02076.1
Psom_0217000.1
0.4
(XXIII)
Mesc_11G084700.9
Mesc_04G074100.2
Ptri_010G019600.1
Grai_006G107300.1
Shim_original_scaffold_4533.1
Shim_original_scaffold_14994.1
HGT
Vvin_203s0038g04460.1
Egra_A01623.1
Gmax_11G010900.3
Gmax_01G228200.1
Caus_C018N0188E0.1
Mgut_F00900.1
Atha_AT4G39160.2
Csat_013980.1
Osat_LOC_Os03g62379.1
0.1
(XXIV)

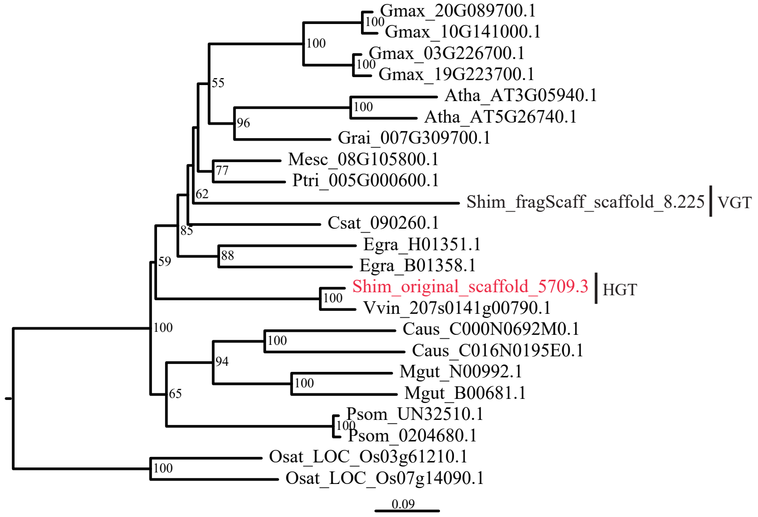

(XXXII)

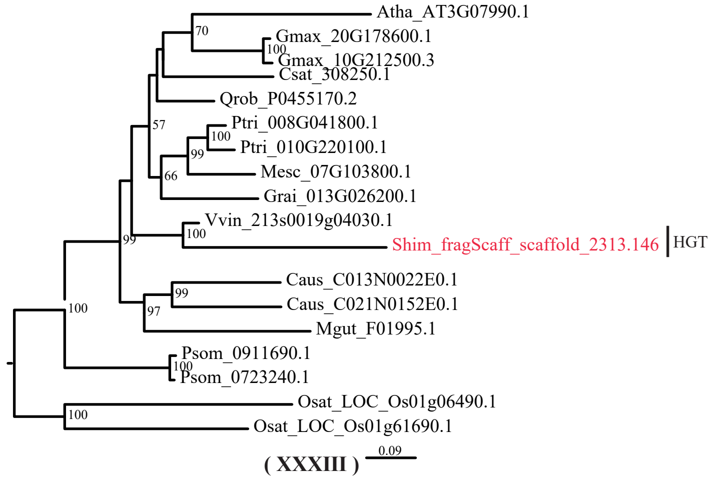

(XXXIII)

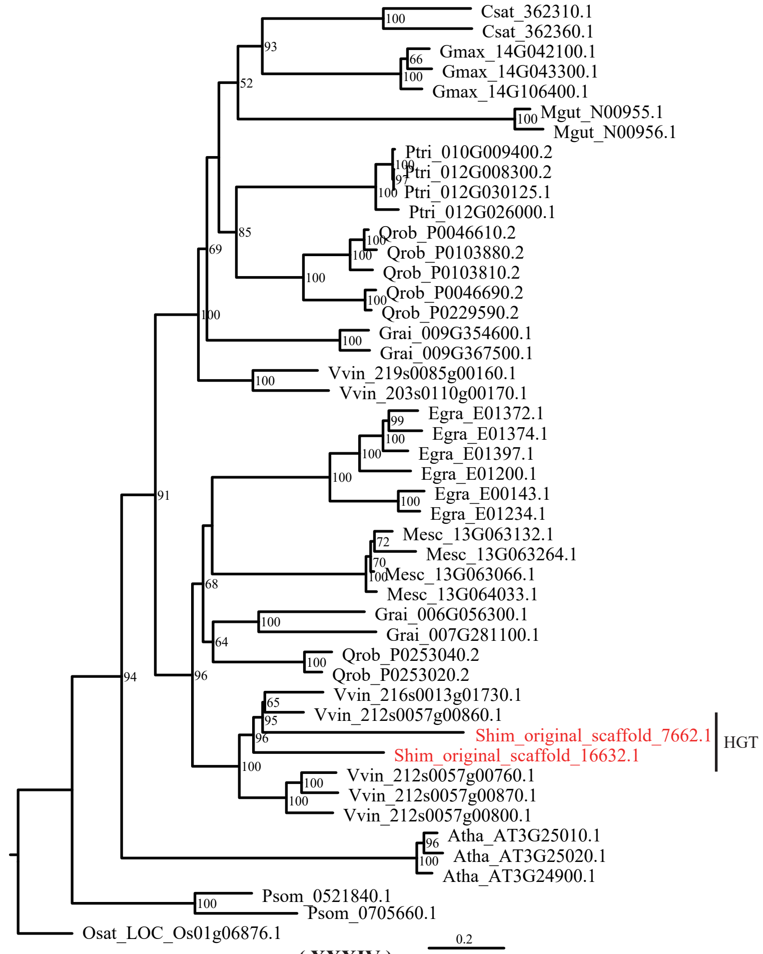

(XXXIV)

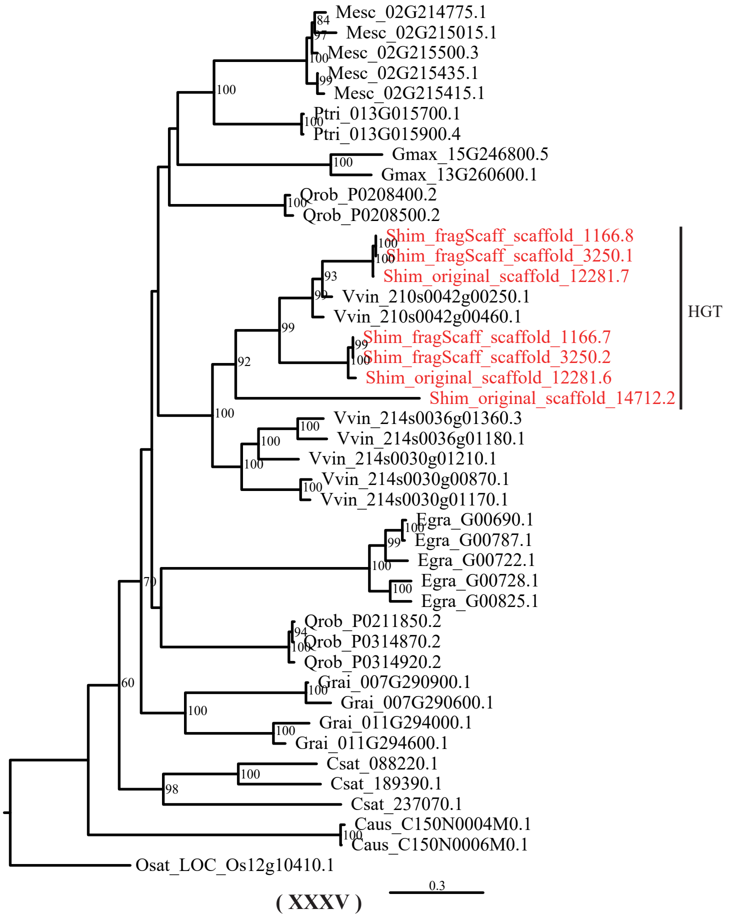

(XXXV)

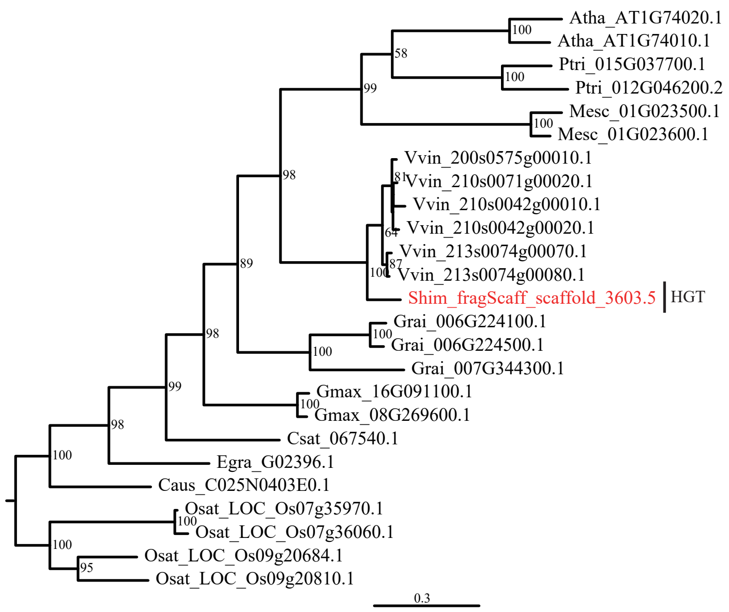

(XXXVI)

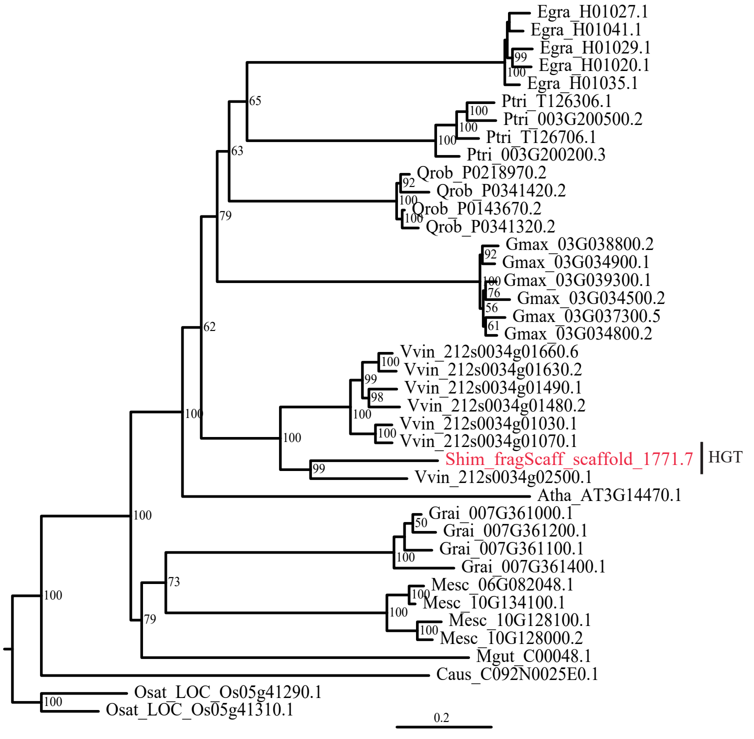

(XXXVII)

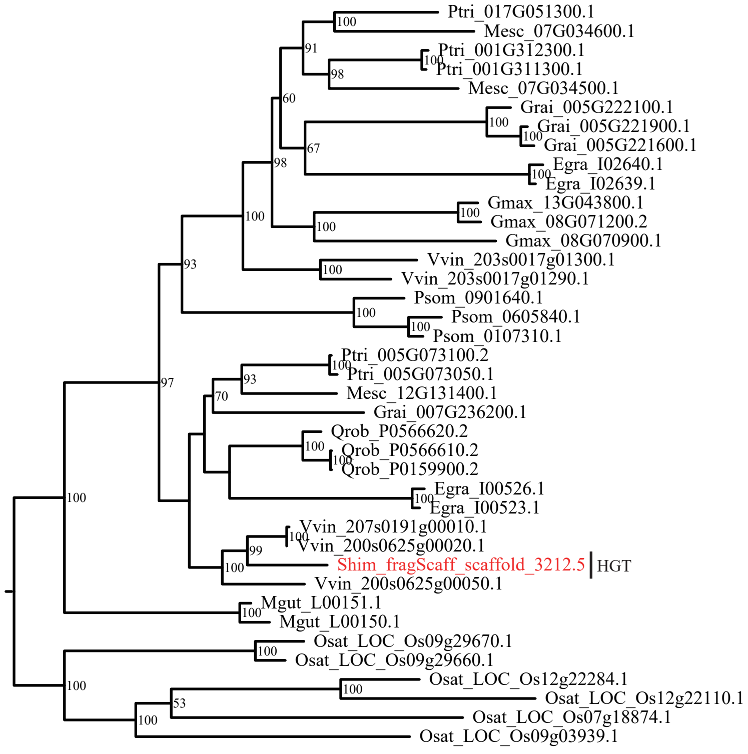

(XXV)

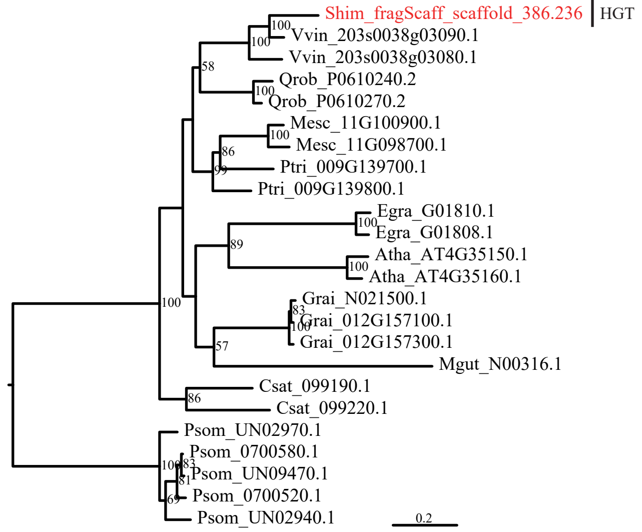

(XXVI)

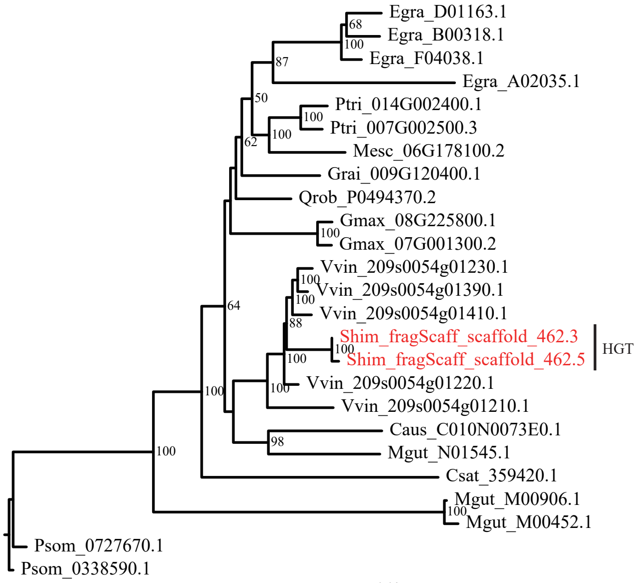

(XXVII)

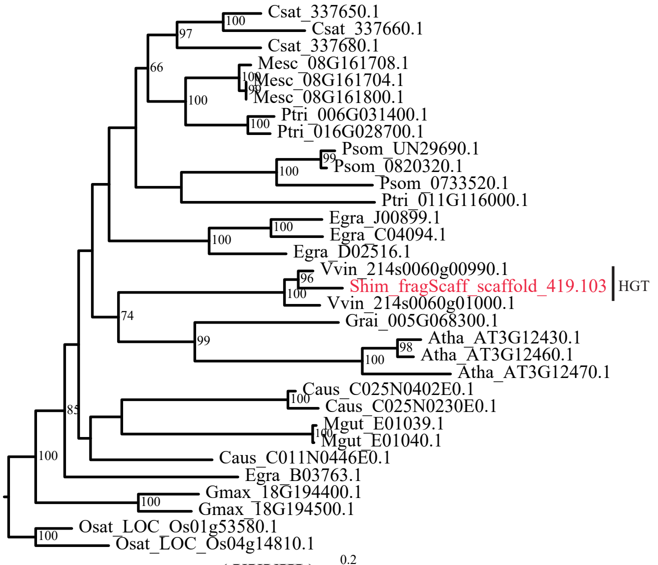

(XXVIII)

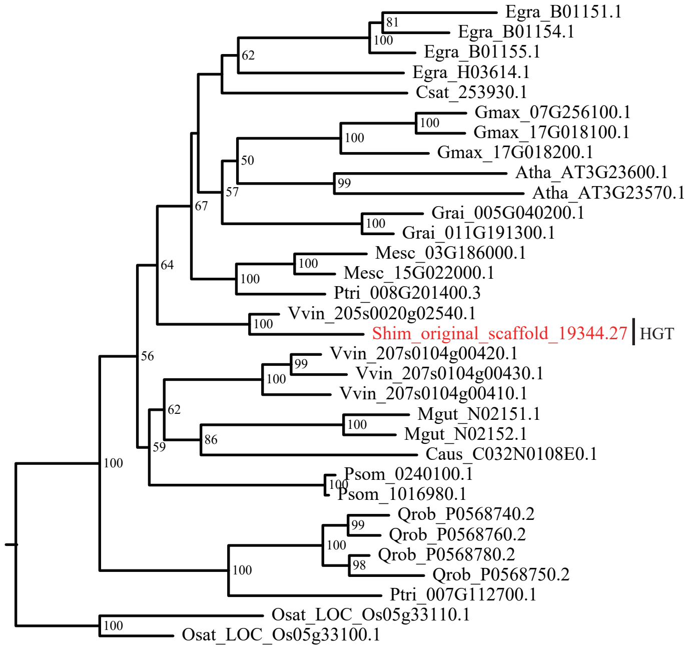

(XXIX)

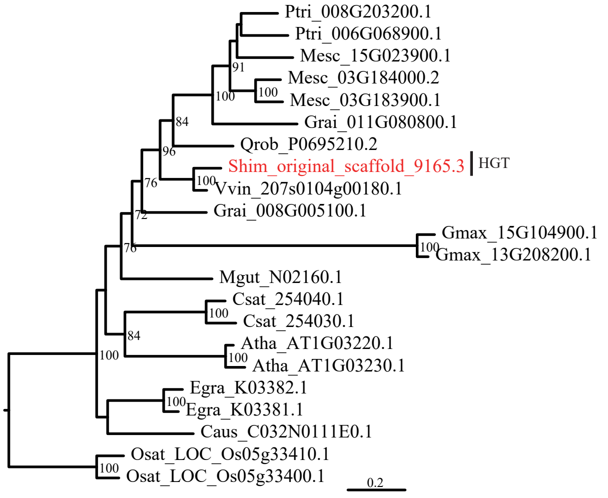

(XXX)

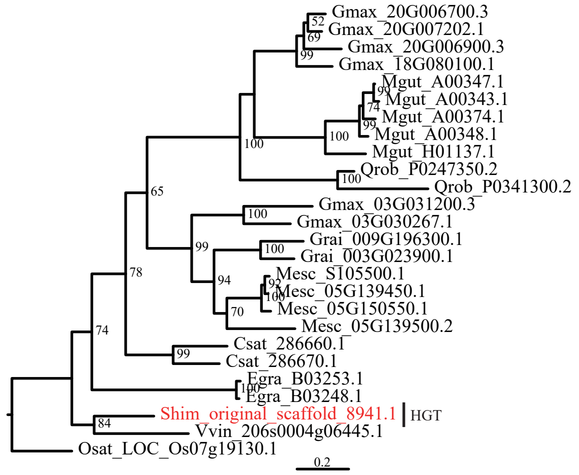

(XXXI)

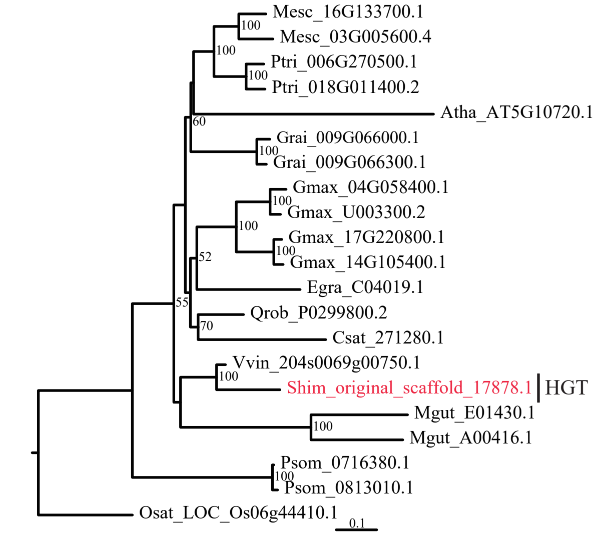

(XXXVIII)

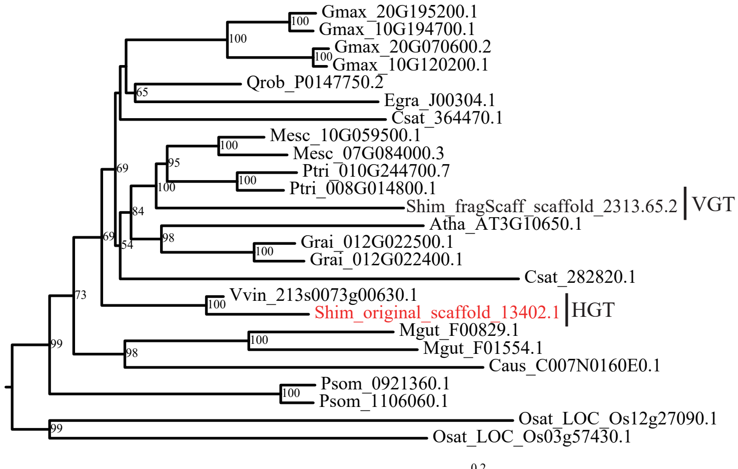

(XXXIX)

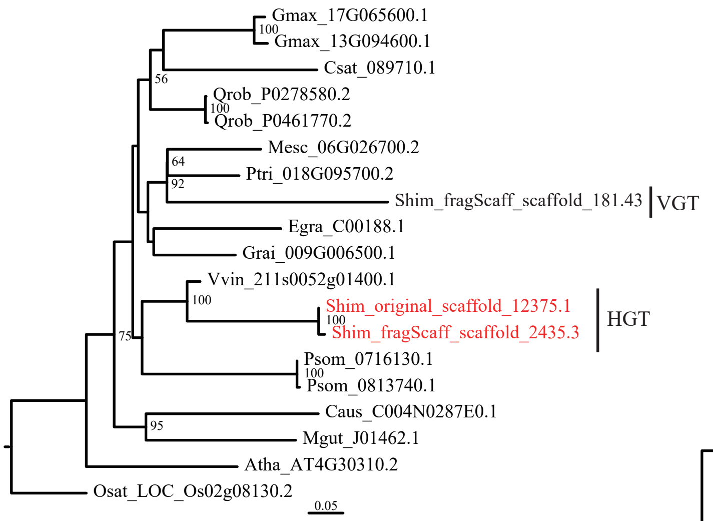

(XL)

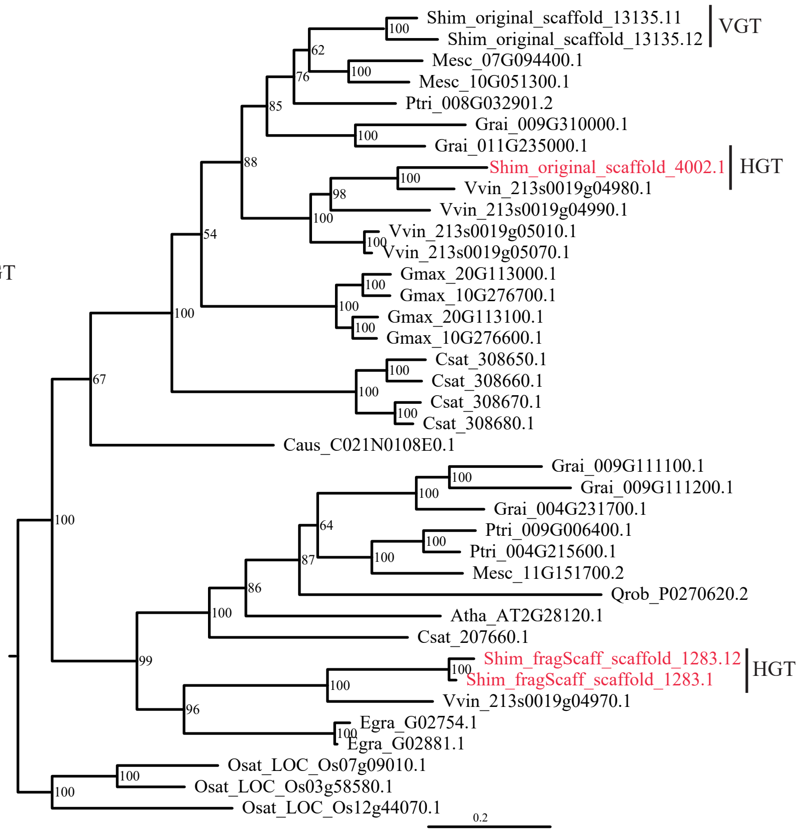

(XLI)

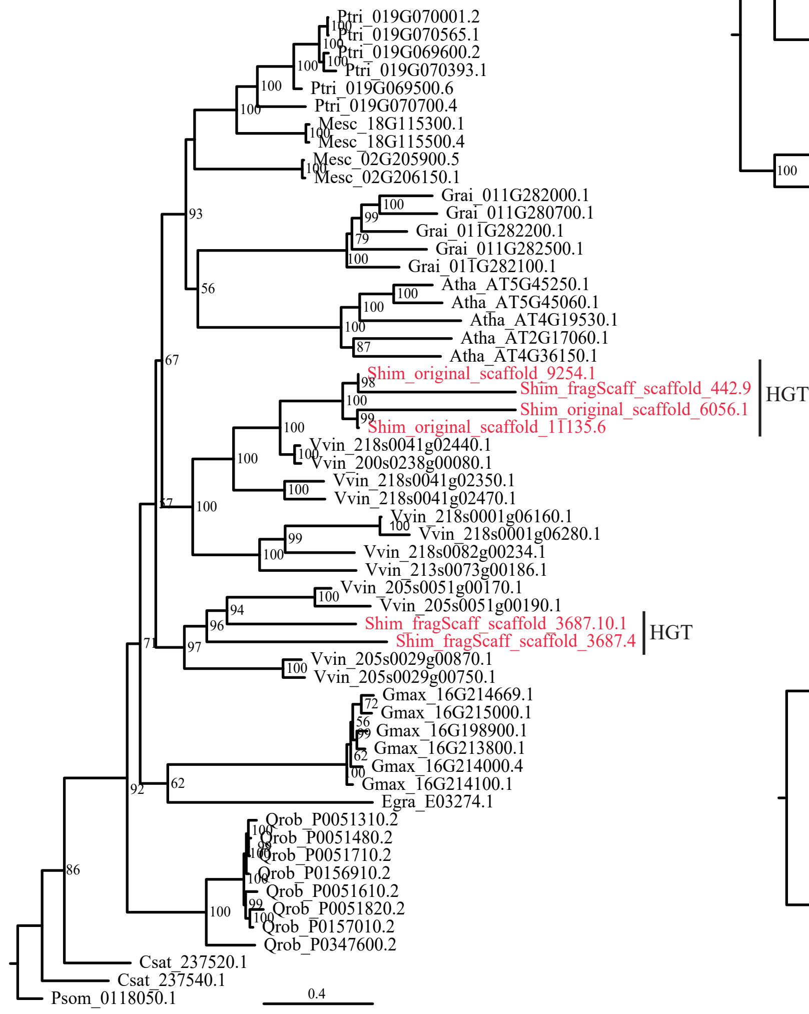

(XLII)

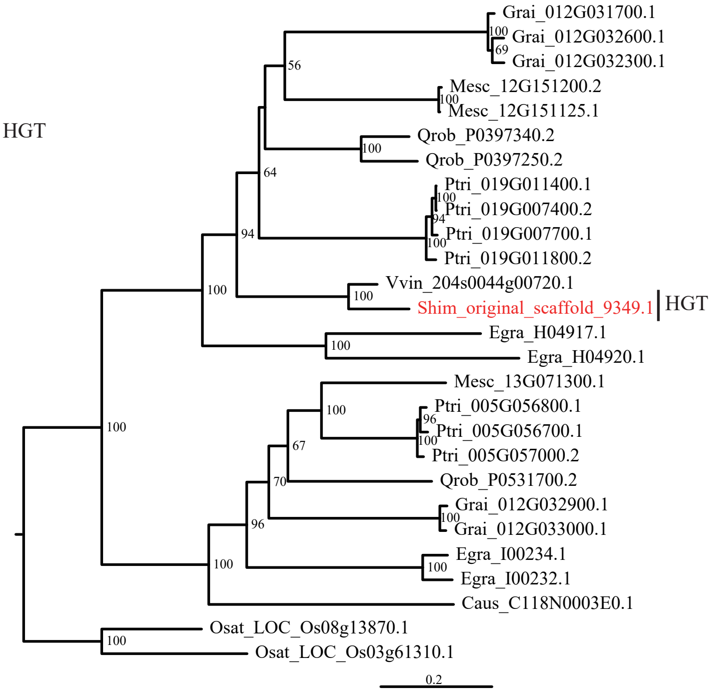

(XLIII)

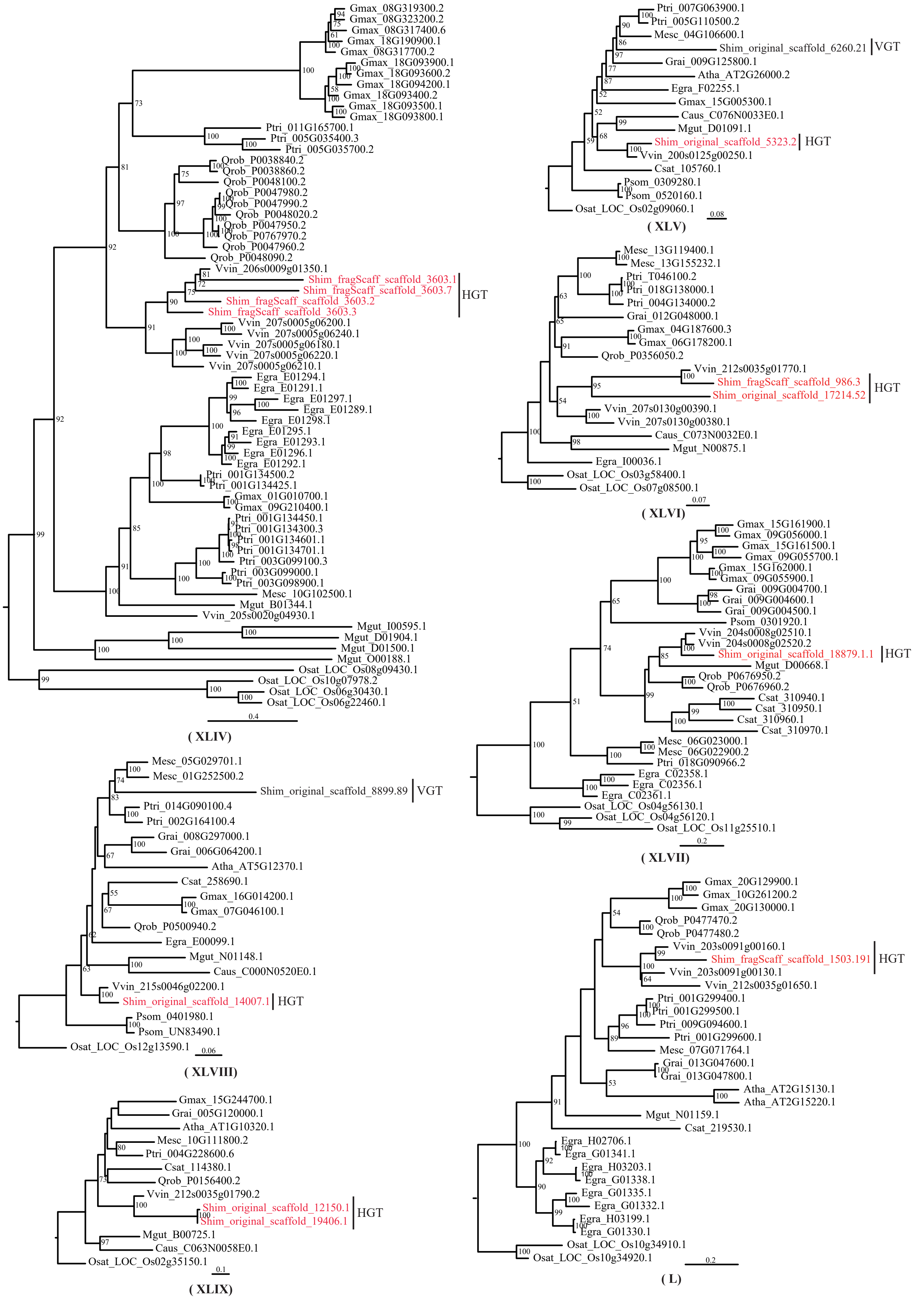

Figure S8. Horizontal gene transfer (HGT) events in the nuclear genome of *Sapria himalayana*. Species name are abbreviated as follows: *Arabidopsis thaliana* (Atha), *Cucumis sativus* (Csat), *Cuscuta australis* (Caus), *Eucalyptus grandis* (Egra), *Glycine max* (Gmax), *Gossypium raimendii* (Grai), *Manihot esculenta* (Mesc), *Mimulus guttatus* (Mgut), *Oryza sativa* (Osat), *Papaver somniferum* (Psom), *Populus trichocarpa* (Ptri), *Quercus robur* (Qrob), *S. himalayana* (Shim) and *Vitis vinifera* (Vvin). HGT events are highlighted in red.

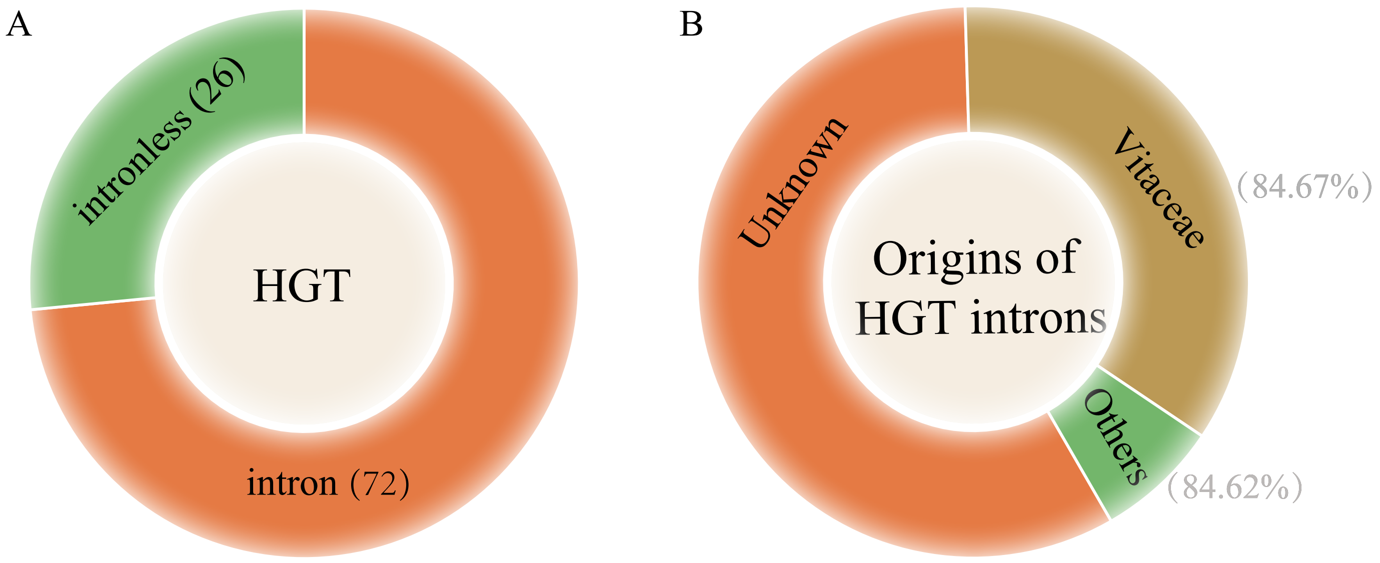

Figure S9. **Overview of intron sequences in HGTs**. (A) 72 HGTs with at least one intron and 26 HGTs without intron; (B) The average value of identity of intron sequence searched on nucleotide (Nt) database at NCBI website using BLASTN. 79 (34.25%) introns from Vitaceae, with average identity was 84.67%, 16 (7.31%) introns from other species, with average identity was 84.62%, and origins of other introns was uncertain.

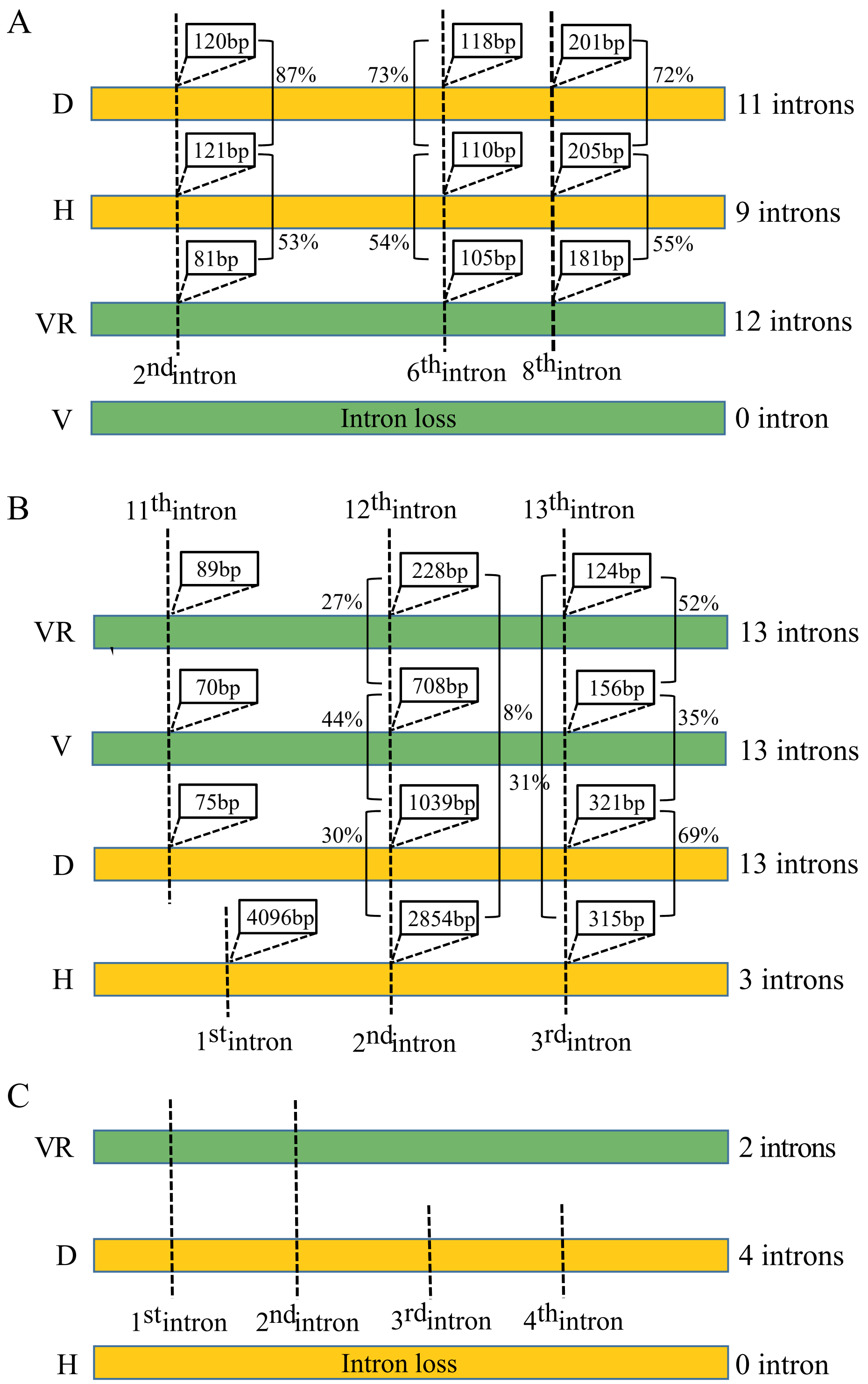

Figure S10. **Gene structure of selected introns in four sequences (D, H, V, and VR).** Yellow and green bars represent coding sequences; the vertical dashed lines represent the intron positions, the boxes represent introns; The value of sequence identity between introns enclosed in square bracket displayed on line. The total number of introns per coding sequence showed a t the right of the bar box. D, genes in donor, substituted by *Vitis vinifera*; H, HGT genes in *Sapria himalayana*; V, vertical genes in *S. himalayana*; VR, related sequences of the vertical gene in *Populus trichocarpa*. (A) The intron positions of three selected introns were highly conserved in D, H, VR sequences, but the V sequence was intronless. (B) The intron positions of two selected introns were highly conserved in D, H, V, VR sequences, but the first intron position of H sequence had changed. (C) the V sequence has been lost, and the H sequences was intronless.

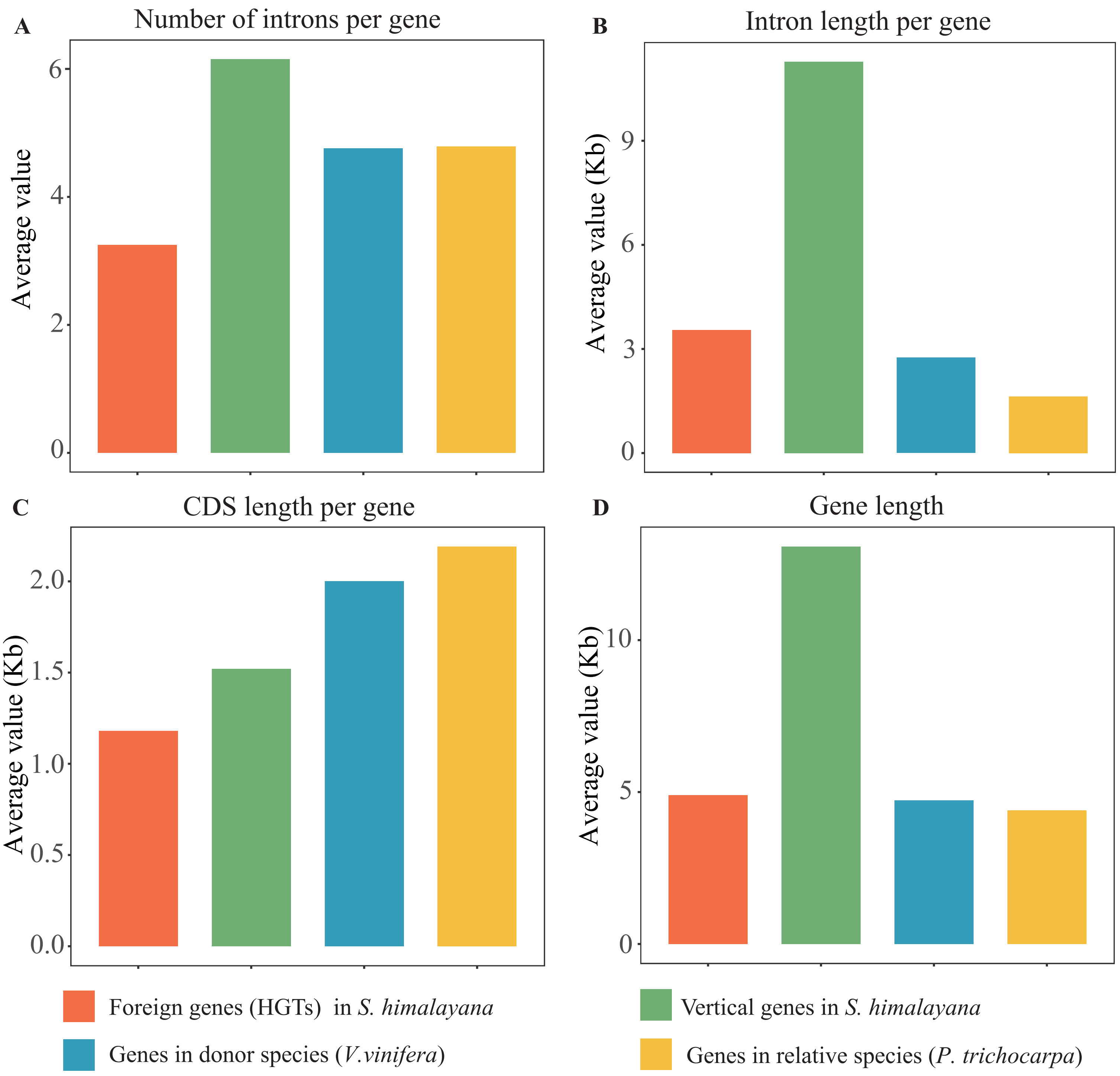

Figure S11. **Comparisons of characteristics among foreign genes (HGTs) in *Sapria himalayana* (red), Vertical genes in *S. himalayana* (green), genes in donor species (blue), and genes in relative species (yellow)**. (A) Average number of introns per gene. (B) Average of total intron length per gene (Kb). (C) Average CDS length per gene (Kb). (D) Average gene length (Kb).

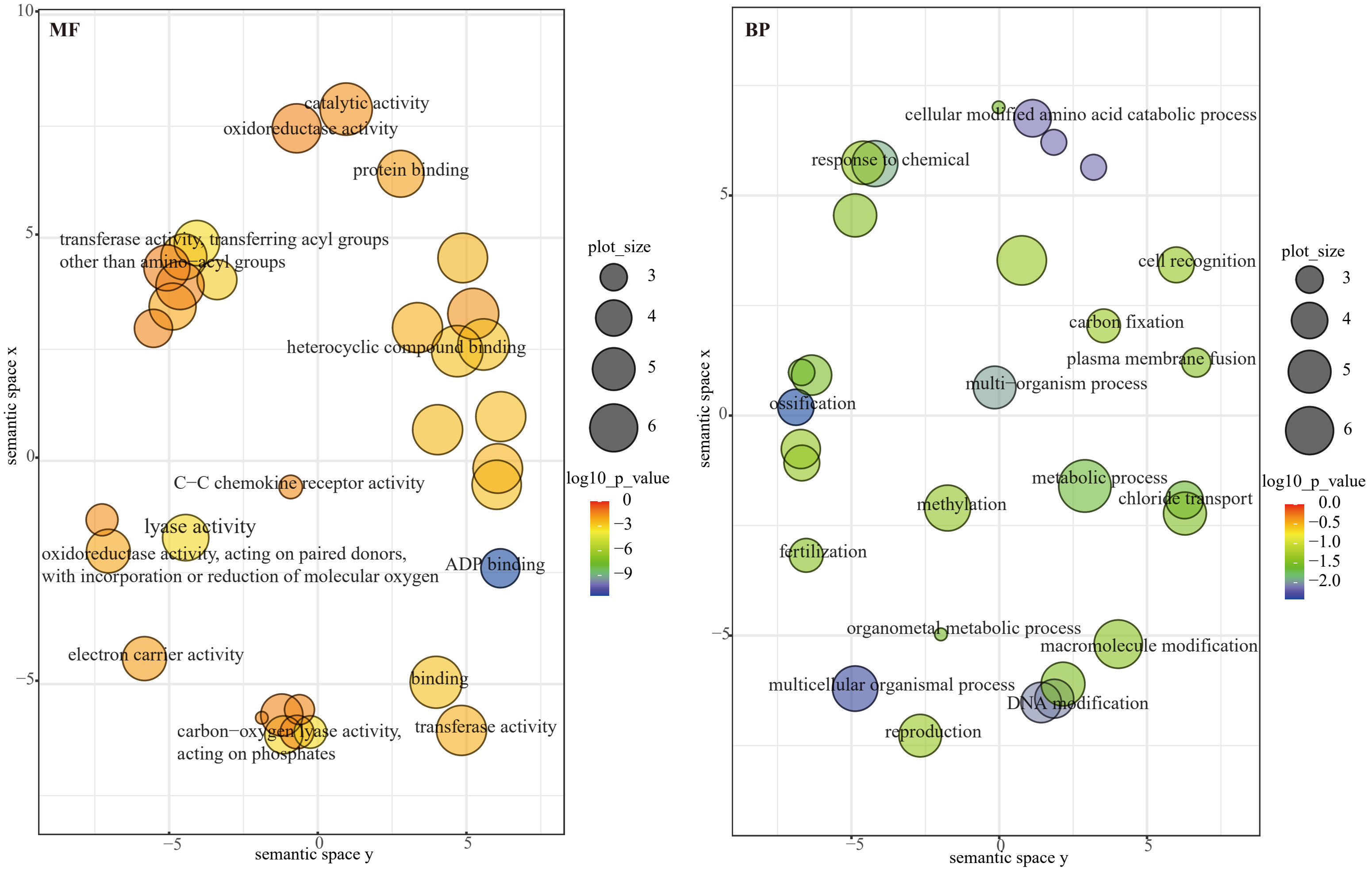

Figure S12. Scatterplot of enriched GO terms in the nuclear horizontal gene transfer (HGT) dataset in *Sapria himalayana*. MF, molecular function. BP, biological process. Bubble color indicates the *P*-value of GO term; bubble size indicates the frequency of the GO term in the underlying GOA database.

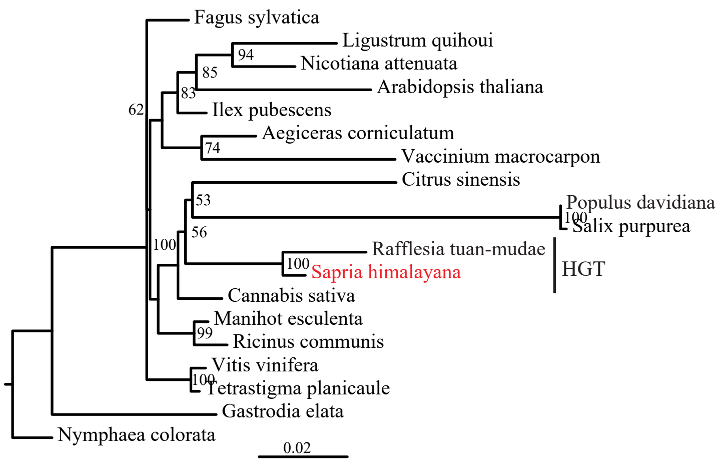

(atp1)

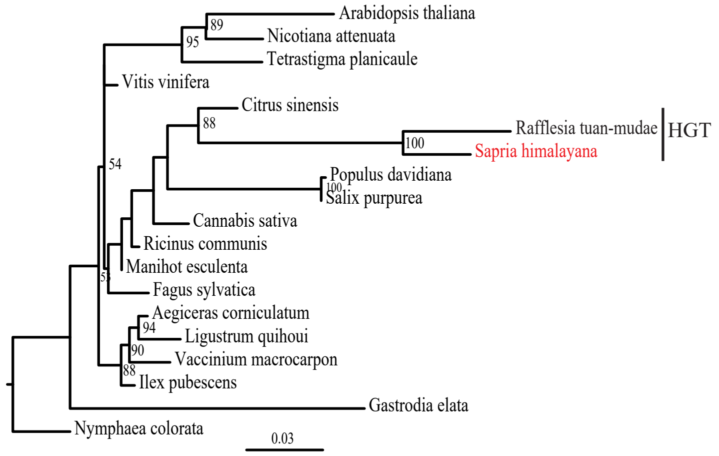

(atp4)

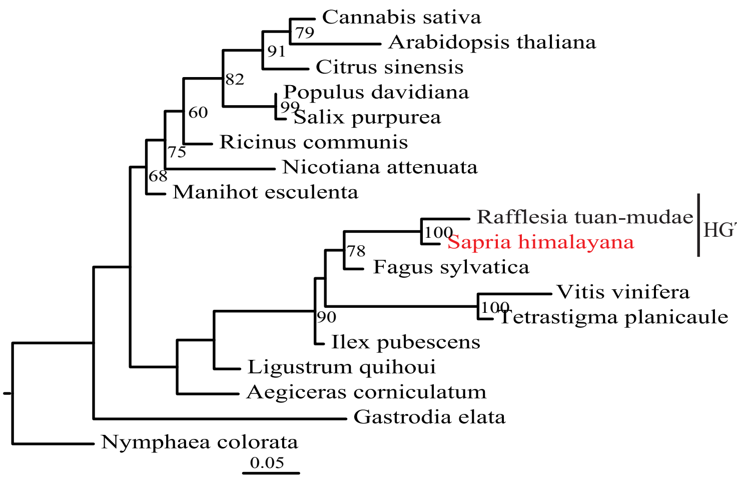

(atp6)

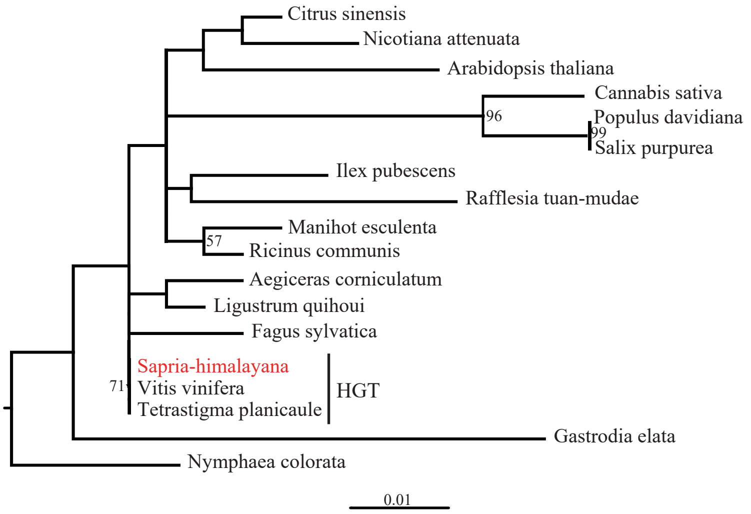

(nad4L)

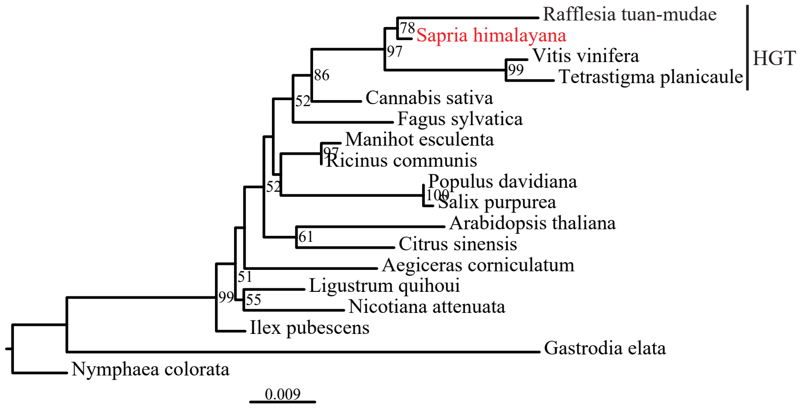

(ccmC)

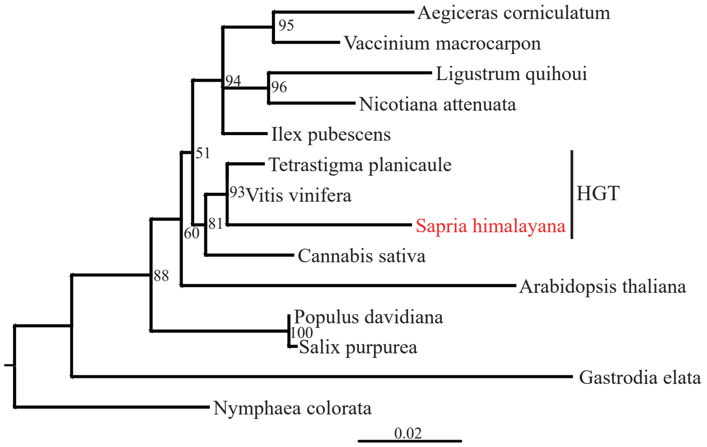

(rpl2)

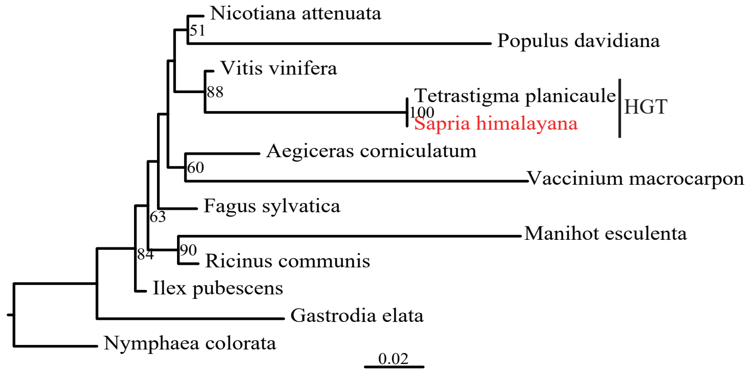

(rps1)

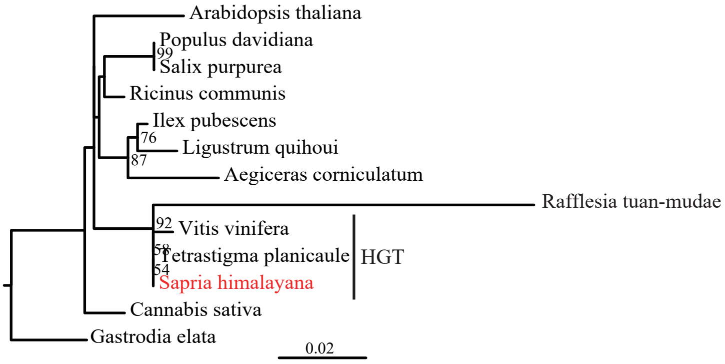

(rps7)

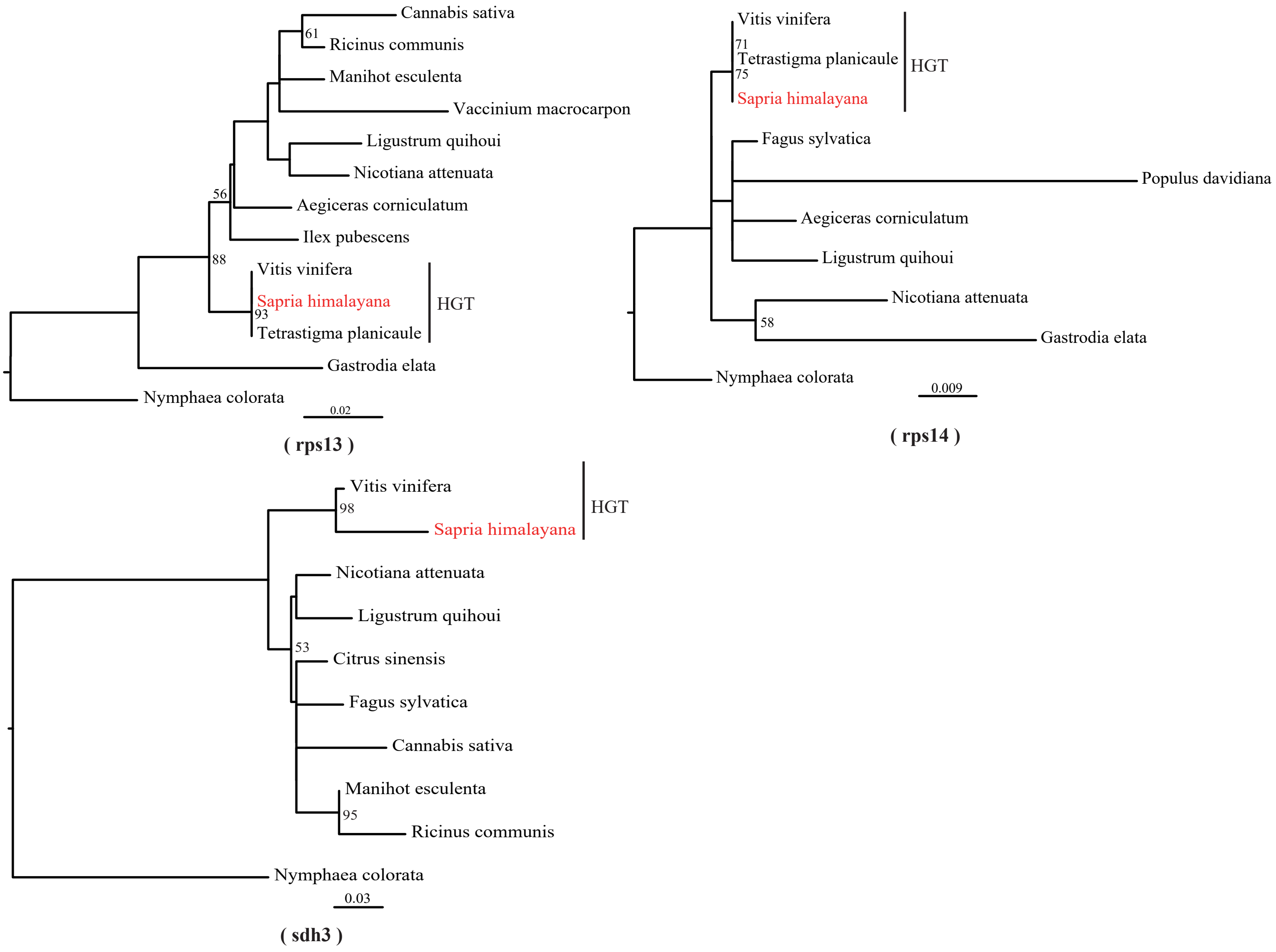

Figure S13. **Mitochondrial horizontal gene transfer (HGT) in *Sapria himalayana***. HGT events are highlighted with red.

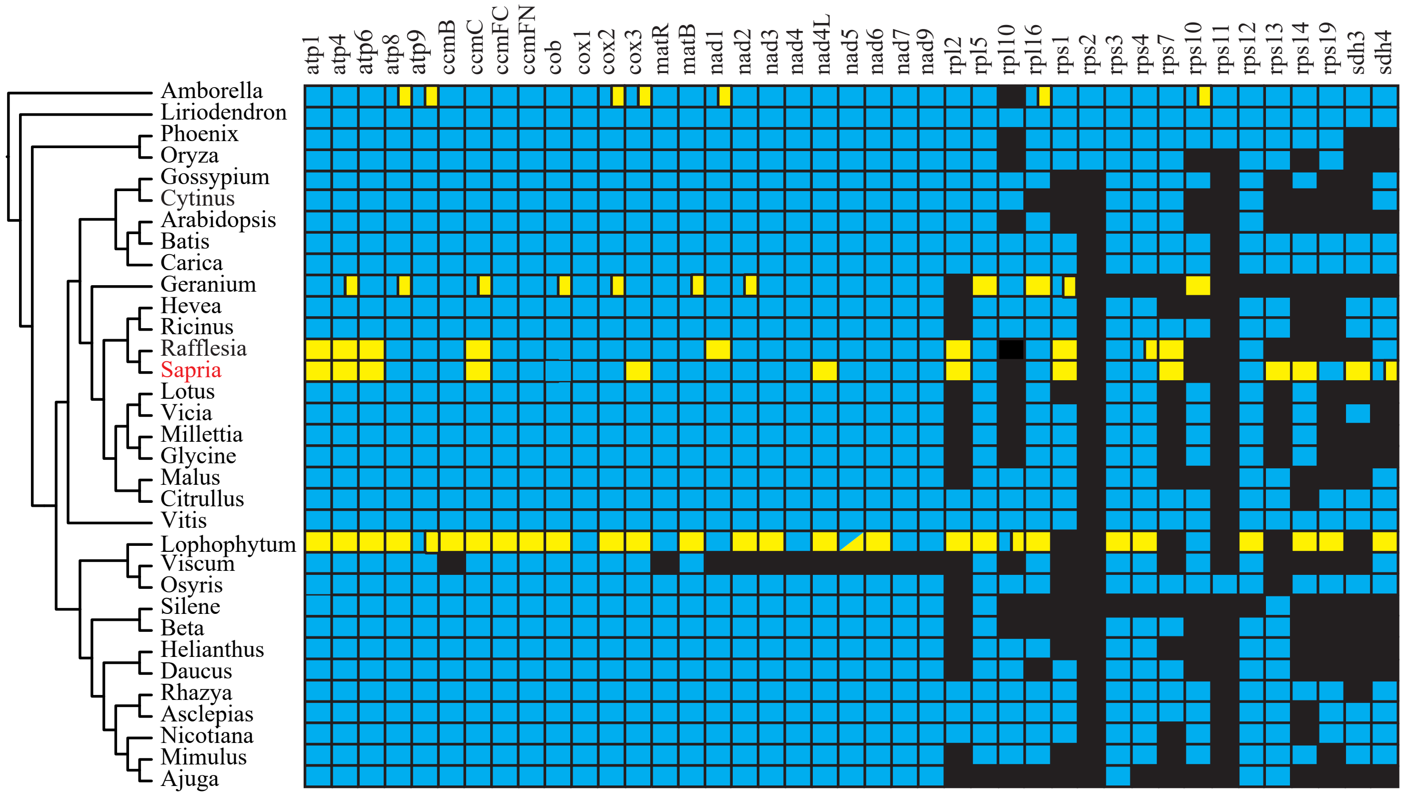

Figure S14. **Comparison of the mitochondrial protein-coding gene content between *Sapria himalayana* and representative angiosperms**. Colors indicate native (blue), foreign (yellow), and absent (black) genes. The presence of both native and foreign copies of a gene is depicted by a subdivided rectangle. The *nad5* gene of *Lophophyphytum* mirabile is a chimeric gene (Sanchez-Puerta et al. 2017). The tree depicts the best estimate of relationships among the plant species. Only intact genes are shown, pseudogenes are not included.

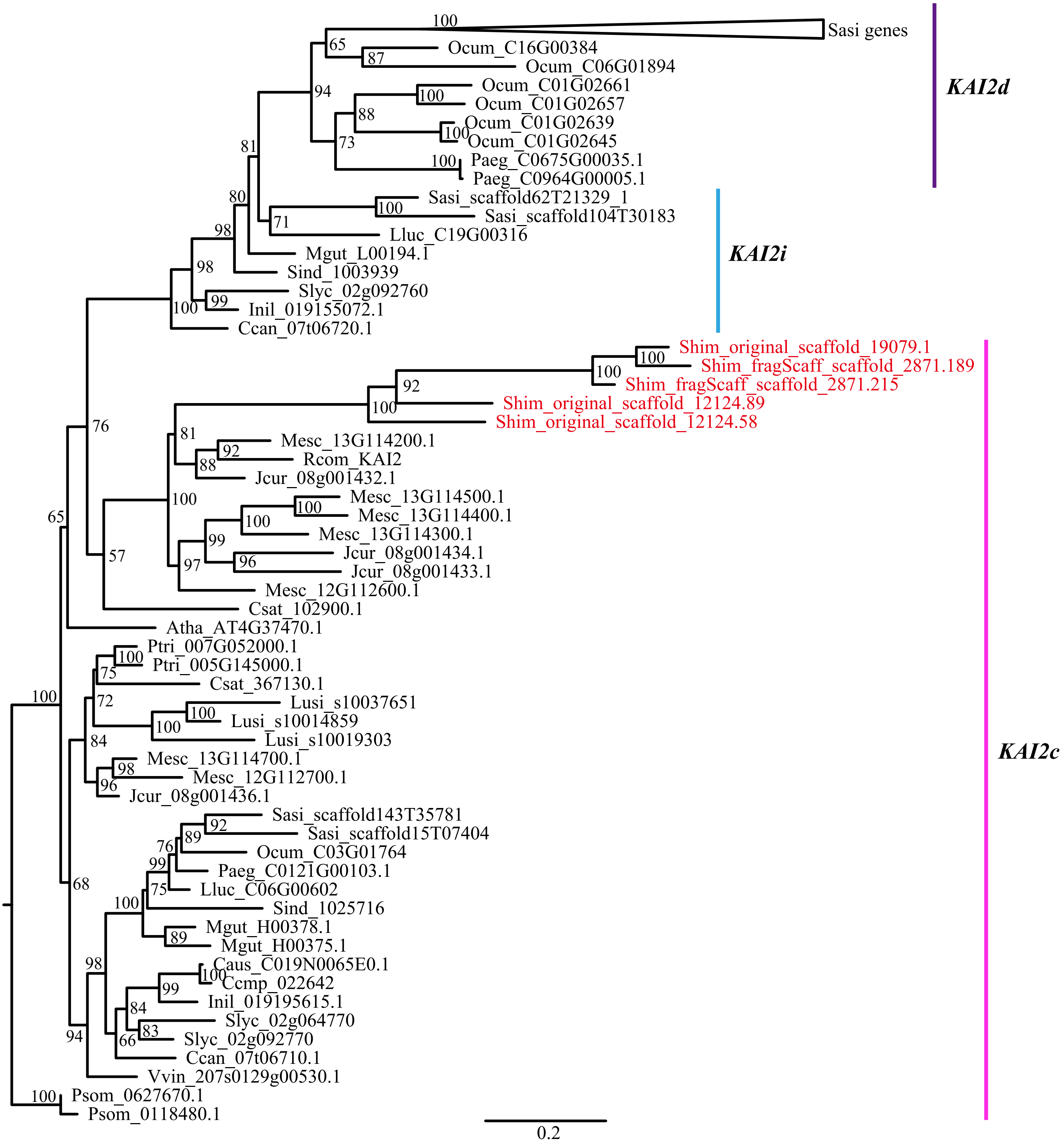

Figure S15. Evolution of *KARRIKIN INSENSITIVE2* (*KAI2*) genes in *Sapria himalayana*. The phylogenetic tree indicates the relationship among the *KAI2* genes in represent species. Conserved (*KAI2c*), intermediate (*KAI2i*), and divergent (*KAI2d*) clades are shown in rose, indigo, and purple, respectively. Species names are abbreviated as follows: *Arabidopsis thaliana* (Atha), *Cuscuta australis* (Caus), *Cuscuta campestris* (Ccam), *Coffea canephora* (Ccan), *Cucumis sativus* (Csat), *Ipomoea nil* (Inil), *Jatropha curcas* (Jcur), *Lindenbergia luchunensis* (Lluc), *Linum usitatissimum* (Lusi), *Mimulus guttatus* (Mgut), *Manihot esculenta* (Mesc), *Mimulus guttatus* (Mgut), *Orobanche cumana* (Ocum), *Phelipanche aegyptiaca* (Paeg), *Papaver somniferum* (Psom), *Populus trichocarpa* (Ptri), *Ricinus communis* (Rcom), *Striga asiatica* (Sasi), *S. himalayana* (Shim), *Sesamum indicum* (Sind), *Solanum lycopersicum* (Slyc), and *Vitis vinifera* (Vvin). Genes in *S. himalayana* are highlighted in red.

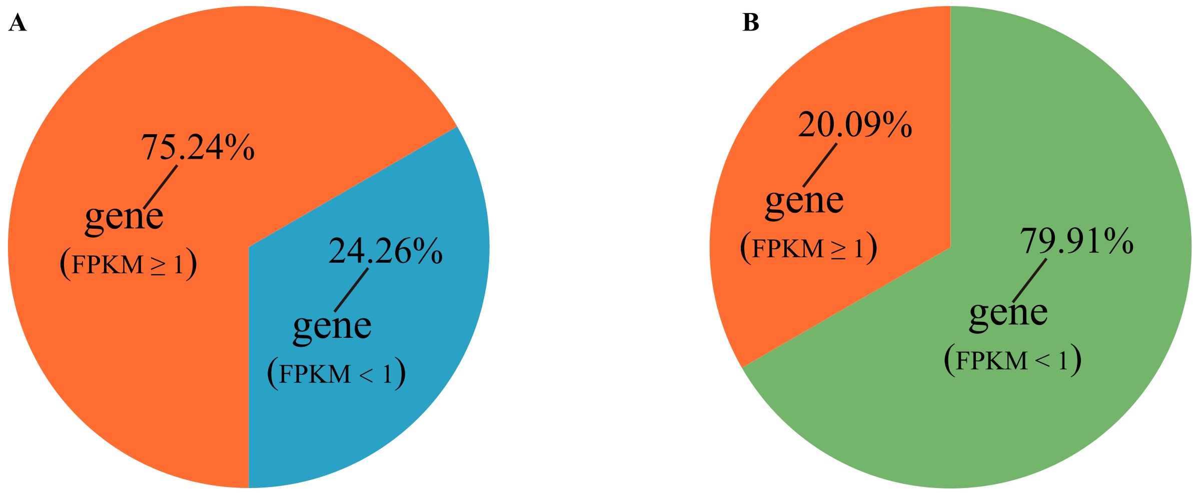

Figure S16. **Overview of gene expression.** The transcriptome data of bract, sepal, petal, and stamen produced in our study, were used to quantify the expression level of gene in our study and Cai et al. [6], respectively. (A) The overview of gene expression in our study. Gene with expression and without expression was highlighted in red and blue, respectively. (B) The overview of gene expression in Cai et al. [6]. Gene with expression and without expression was highlighted in red and green, respectively.

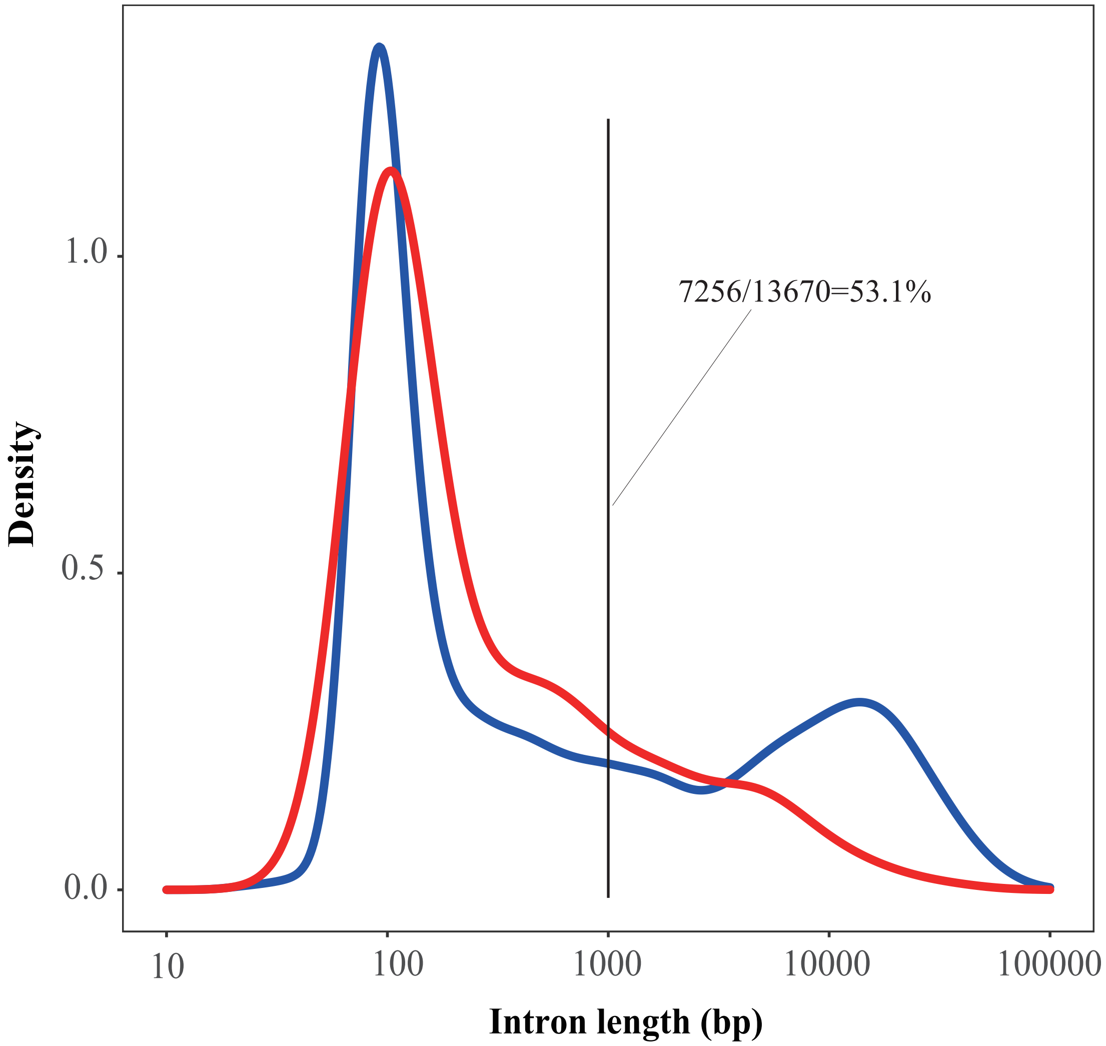

Figure S17. **Intron length disparity**. Intron length distribution of genes (blue line) and HGTs (red line) in *Sapria himalayana* genome. 7256 (53.1%) genes had at least one long intron (>1Kb).

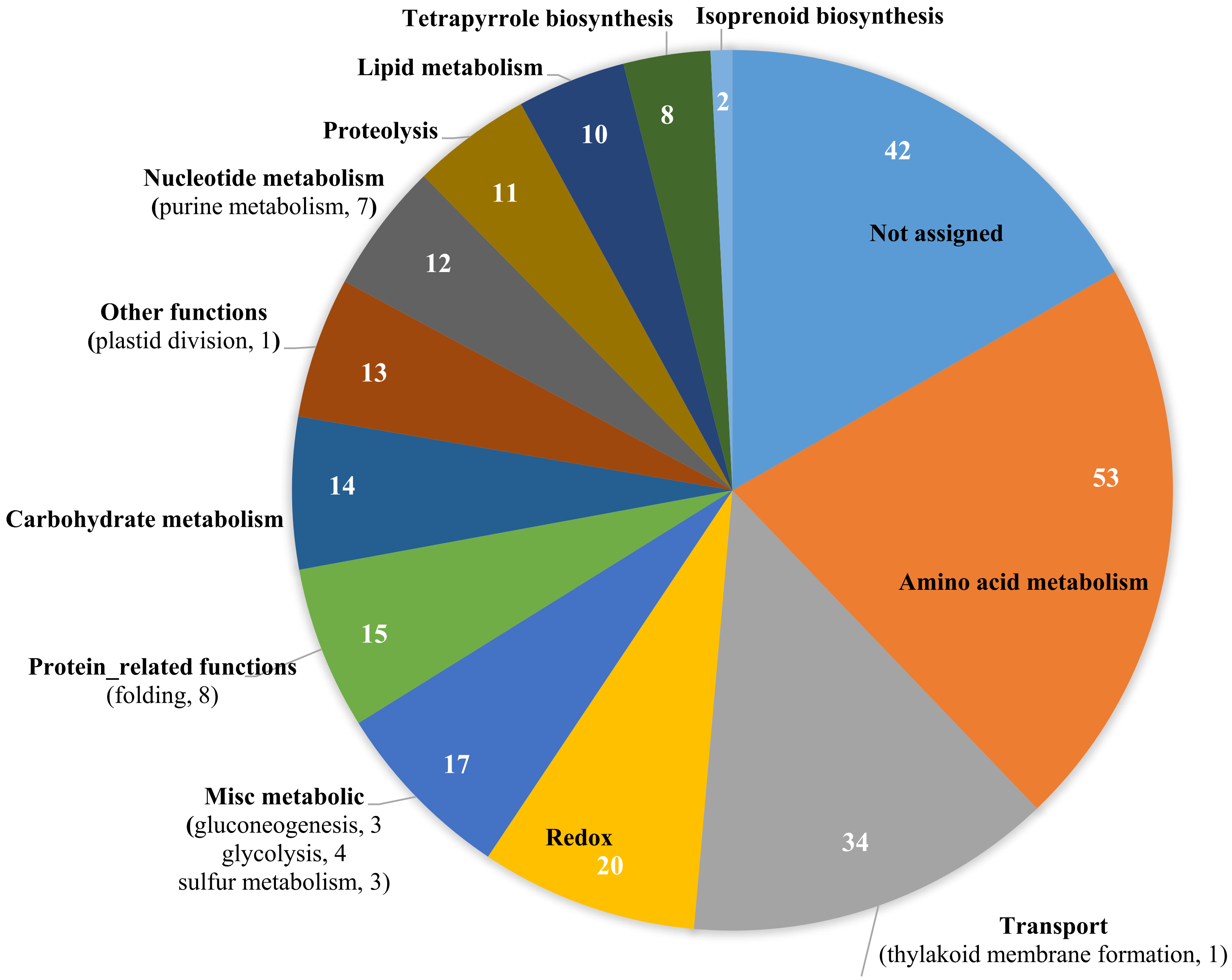

Figure S18. Number of nuclear-encoded plastid-targeting genes in different categories from *Sapria himalayana* genome.

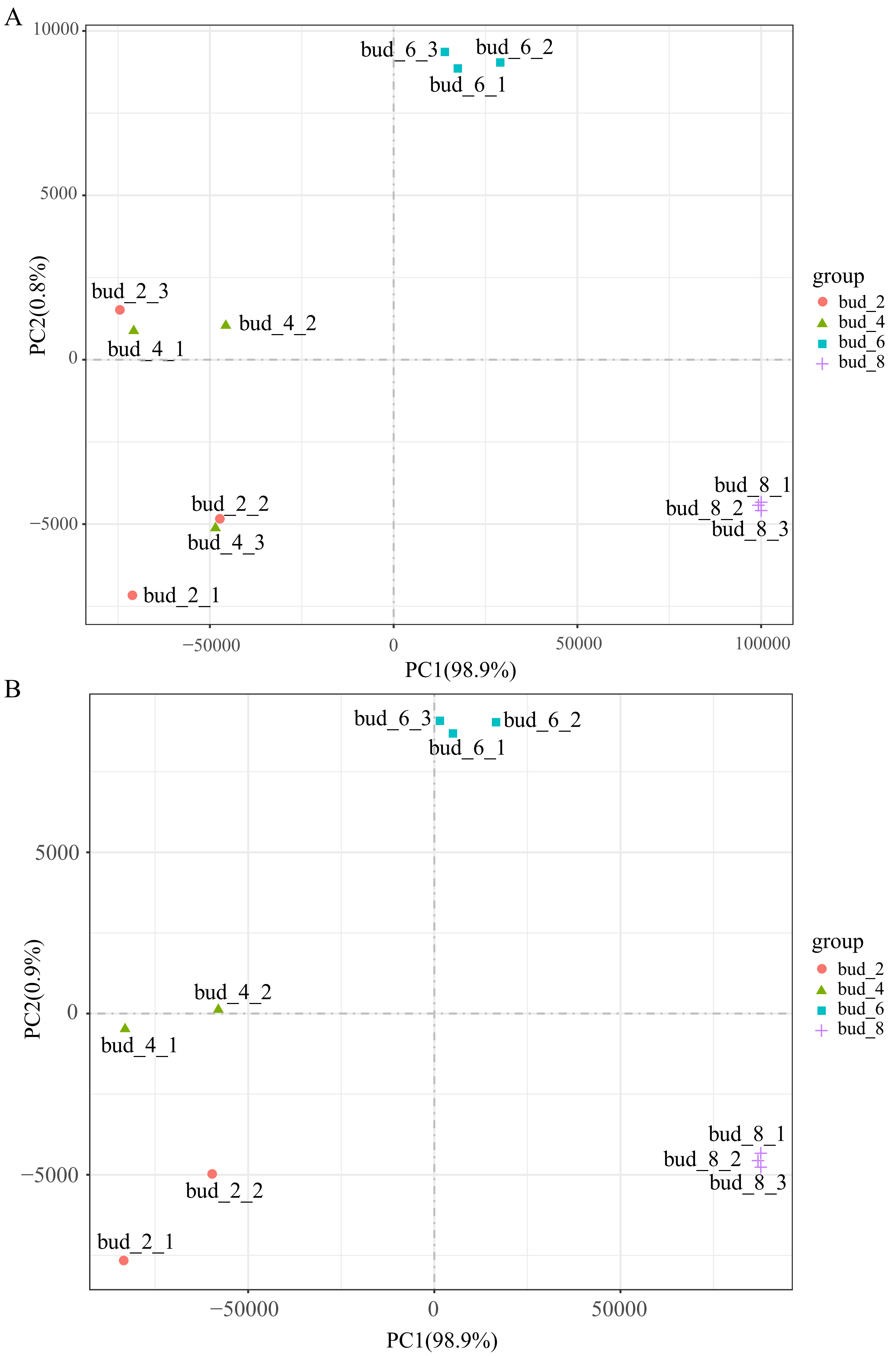

Figure S19. **Principal component analysis among samples from unopened flower bud of *Sapria himalayana*.** (A) All samples for transcriptome; (B) Removal of abnormal samples for transcriptome.

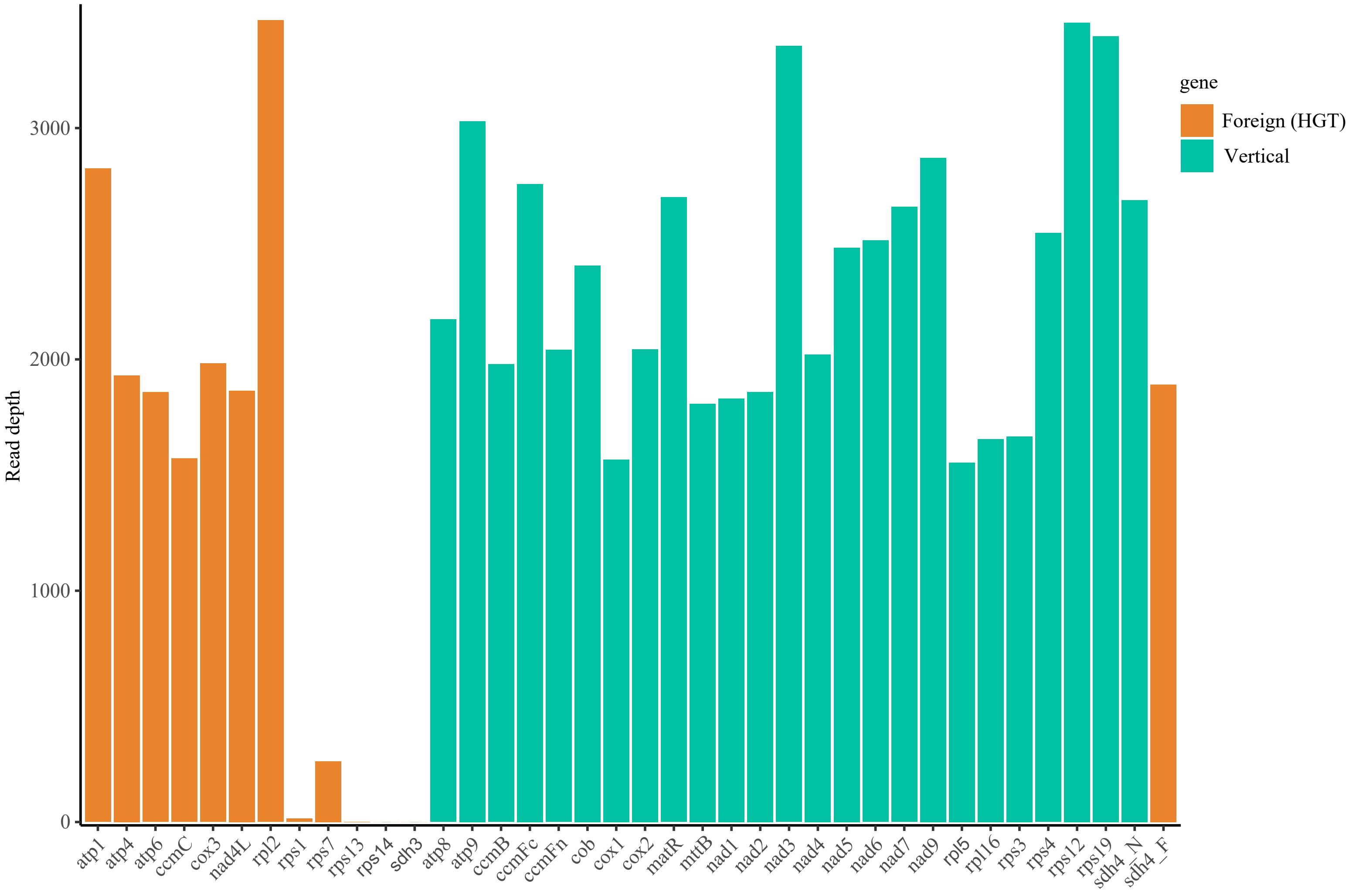

Figure S20. **Sequencing depths of intact mitochondrial protein-coding genes in *Sapria himalayana***. *Sdh4_N* represents the vertical copy of *sdh4*, and *sdh4-F* represents the foreign copy.
